# Catalyst and solvent-free solid–solid melt synthesis of multi-colour emissive 2-functionalized quinoxaline-based fluorophores

**DOI:** 10.1039/d6ra01059h

**Published:** 2026-04-24

**Authors:** Sai Teja Talari, Rama Mohana Reddy Sirigireddy, Siva Dakshayani Vadanapalli, Mohan Das Dasappa, Sultana Shaik, Venkatramu Vemula, Eethamukkala Ubba, Ramamohana Reddy Maddike, Haranath Divi, Chinna Gangi Reddy Nallagondu

**Affiliations:** a Green & Sustainable Synthetic Organic Chemistry and Optoelectronics Laboratory, Department of Chemistry, Yogi Vemana University Kadapa-516005 Andhra Pradesh India ncgreddy@yogivemanauniversity.ac.in; b Department of Physics, Yogi Vemana University Kadapa-516 005 Andhra Pradesh India; c Department of Chemistry, Andhra Kesari University Ongole-523 001 Andhra Pradesh India; d Lincoln University College Petaling Jaya 47301 Selangor Darul Ehsan Malaysia; e Luminescent Materials and Devices Group, Department of Physics, National Institute of Technology Warangal-506004 Telangana India

## Abstract

A rapid, highly efficient and practically viable solid–solid melt reaction (SSMR) protocol has been developed for the synthesis of multi-colour-emissive quinoxaline-based small organic fluorophores (QBSOFs, 3) from readily available *o*-phenylenediamines (1) and α-bromoketones (2) or arylglyoxals/glyoxylic acids (4) under solvent- and catalyst-free conditions. This environmentally benign methodology features operational simplicity, avoidance of cost-intensive and scale-restrictive techniques such as microwave or ultrasonic irradiation, broad substrate compatibility with excellent functional-group tolerance, and a straightforward work-up affording products in high purity. Notably, the reactions proceed rapidly to deliver excellent to near-quantitative yields (95–99%). The successful gram-scale synthesis of 2-(4-chlorophenyl)quinoxaline (3a) further underscores the economic feasibility and industrial applicability of this approach for large-scale production of 2-arylquinoxalines. The solid-state photophysical properties of the synthesized quinoxaline fluorophores were systematically investigated. The compounds exhibit tunable solid-state emission spanning from purplish blue to the yellow light region, primarily governed by the nature of substituents at the 2-position of the quinoxaline core. Remarkably, compounds 4-(quinoxalin-2-yl)benzonitrile (3g) and 4-(6,7-dimethylquinoxalin-2-yl)benzonitrile (3u) display cold-white and warm-white light emission, respectively. Furthermore, the HOMO and LUMO energy levels are comparable to those of reported hole-transporting materials (HTMs), highlighting the dual luminescent and hole-transporting characteristics of these fluorophores. Consequently, these multi-colour-emissive QBSOFs, with intrinsic hole-transporting characteristics could be suitable for application in organic optoelectronic devices.

## Abstract 2

A rapid, highly efficient and scalable solid–solid melt reaction (SSMR) strategy has been developed for synthesizing multi colour-emissive and hole-transporting quinoxaline-based small organic fluorophores under solvent- and catalyst-free conditions.

## Graphical abstract

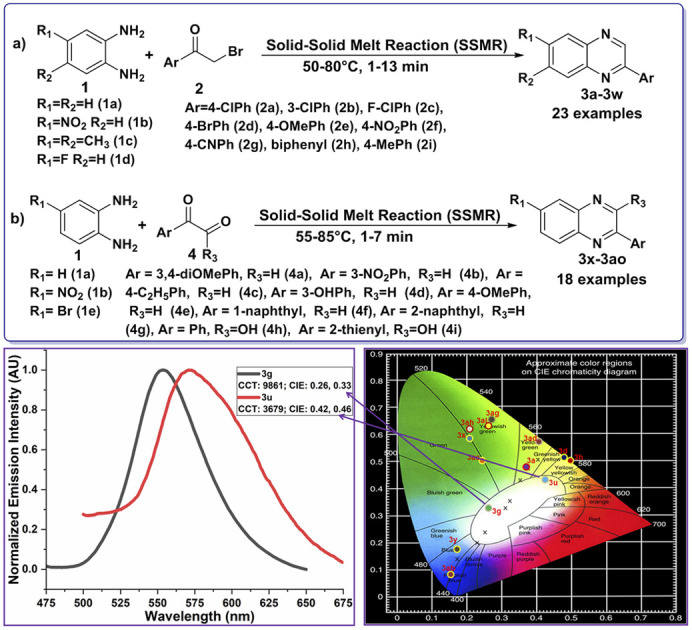

## Introduction

**Fig. 1:**
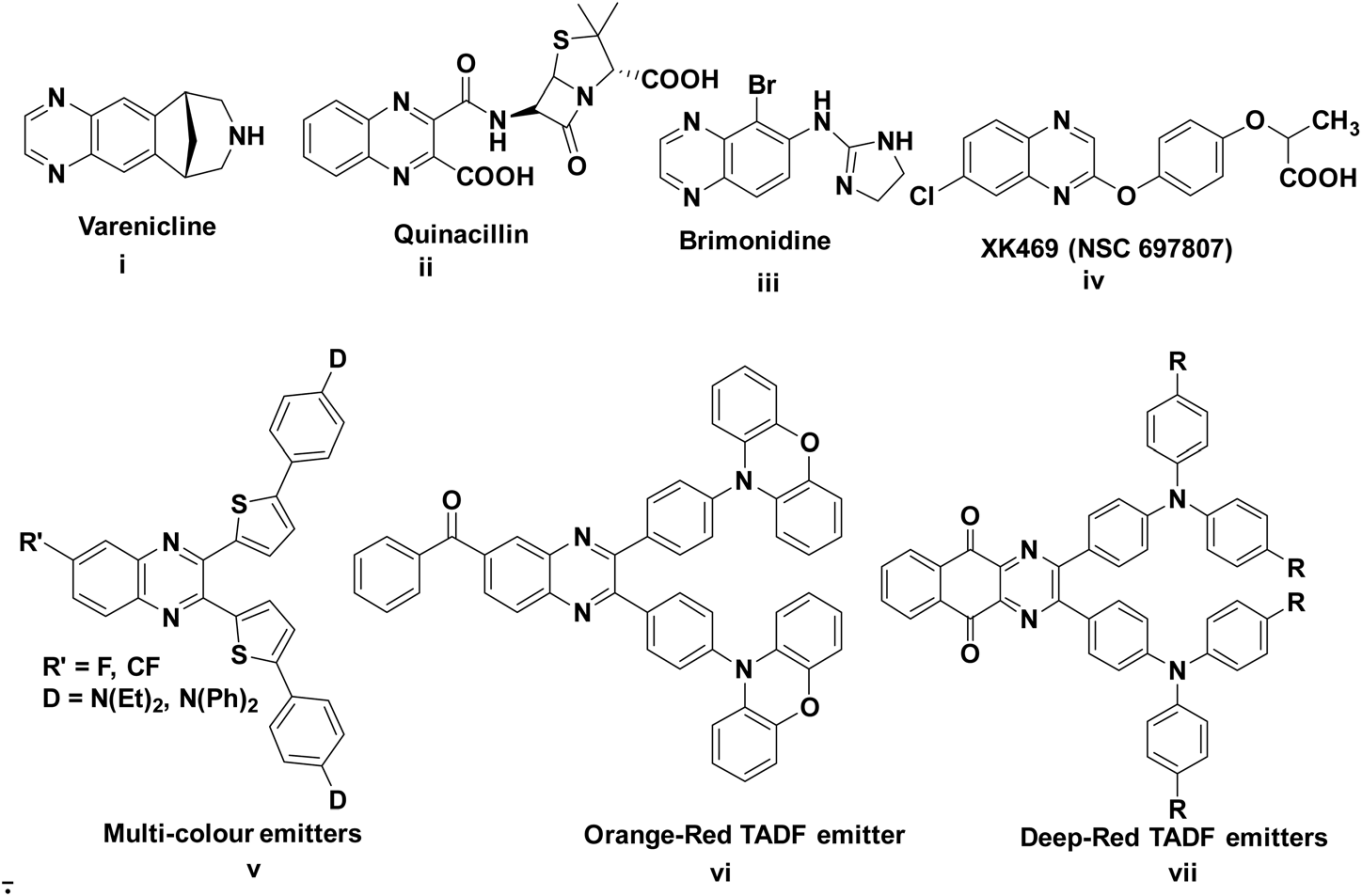
Representative examples of quinoxaline-based pharmaceutical drug molecules (i–iv) and fluorescent materials (v–vii).

Organic optoelectronic materials have received great attention owing to their potential applications in electronic and optoelectronic devices such as organic light-emitting diodes (OLEDs), organic solar cells (OSCs), organic field-effect transistors (OFETs), organic fluorescent sensors, *etc*.^1,2^ Particularly, nitrogen-based heterocycles play a vital role in both medicinal chemistry and material sciences.^3–8^ Amongst various nitrogen heterocycles, the quinoxaline-based compounds have received substantial attention in the area of organic electronics because of their promising electronic properties, structural flexibility, colour tunability, high thermal and photochemical stability, *etc.* Hence, quinoxaline-based materials have wide range of applications in organic photovoltaic devices, organic semiconductors, electroluminescent materials, fluorescent probes, OLEDs, OSCs, sensitizers for dye-sensitized solar cells, polymer light-emitting diodes (PLEDs) and fuel cells.^9–24^ Quinoxalines have not only a broad range of applications in optoelectronics but also have potential applications in biology and agricultural fields.^25–32^ Some of the quinoxaline based pharmaceutical drug molecules (i–iv) and fluorescent materials (v–vii) are presented in Fig. 1.

On account of their widespread applications in both biology and optoelectronics, several synthetic methods have been developed for the preparation of quinoxaline derivatives.^33–38^ In general, quinoxalines have traditionally been synthesized using classical methodologies such as the Körner method^39^ and the Hinsberg method.^40^ These approaches typically involve the condensation of *o*-phenylenediamines (*o*-PDs) with 1,2-dicarbonyl compounds to yield 2,3-disubstituted quinoxalines. In recent years, considerable efforts have been devoted for developing greener and more sustainable protocols, including the use of heterogeneous eco-friendly catalysts, environmentally benign solvents, and alternative green technologies for this transformation.^33,38,41–64^

Furthermore, 2-substituted quinoxalines are commonly synthesized *via* the reaction of *o*-phenylenediamines (*o*-PDs) with α-bromoketones. This transformation has been achieved using a variety of catalysts and solvents to enhance reaction efficiency.^65–84^ Notably, some effective catalyst-free methodologies have also been reported.^85,86^

Though the above reported methods are efficient to provide quinoxaline derivatives, they suffer from one or more shortcomings such as longer reaction times, high catalyst loading, narrow substrate scope, requirement of expensive techniques, use of toxic and expensive catalysts, reagents & solvents, tedious workup process required to obtain pure product, lot of waste generation, unsuitability for industrial applications, *etc.* Therefore, the development of environmentally benign and industrially viable synthetic routes that overcome the aforementioned disadvantages for the synthesis of 2-substituted quinoxalines remains an active area of research and a challenging task.

Solid–solid melt reaction (SSMR) strategy is considered as one of the nature friendly synthetic routes and has been successfully employed in the synthesis of bio active organic compounds.^87–93^ In this strategy, the mixture of solid reactants is melted at its eutectic temperature. Easy to setup, eco-friendly, highly efficient, rapid reaction rates, more selective, needlessness of solvents, quantitative yields of products in shorter reaction times, pure products obtained without need of chromatographic techniques, no tedious workup procedure, reduced waste and scalability are the noteworthy advantages of the SSMR strategies.^88,94–96^ The mentioned merits of SSMR strategies are highly suitable for industrial applications. Further, to the best of our knowledge, there are no reports on SSMR strategy for the synthesis of 2-arylquinoxalines from *o*-phenylenediamines (1) and α-bromoketones (2) or arylglyoxals/glyoxylic acids (4).

**Scheme 1:**
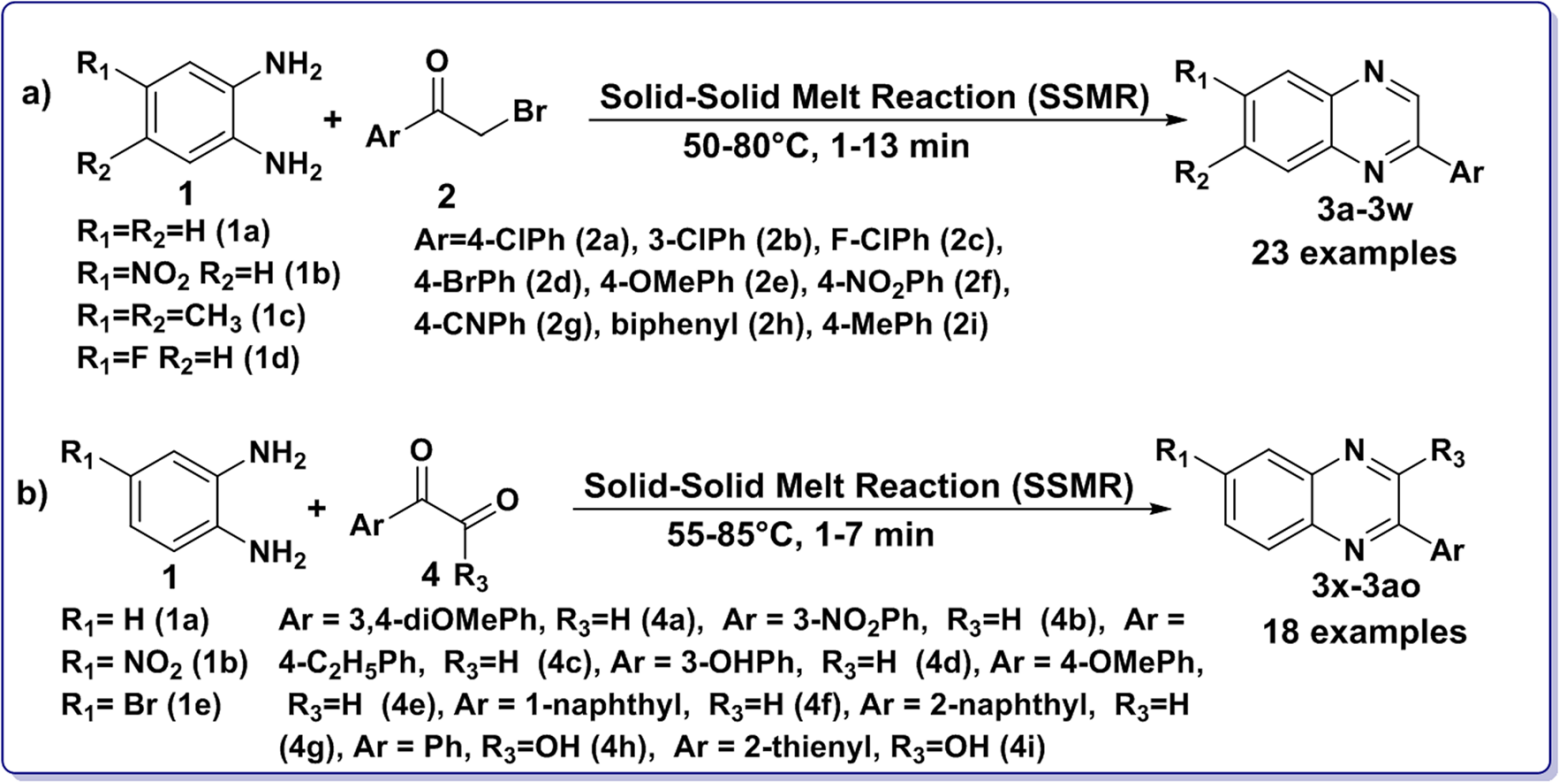
Catalyst and solvent free solid–solid melt reaction (SSMR) strategy for the synthesis of 2-arylquinoxalines (3) from (a) *o*-phenylenediamines (1) and α-bromoketones (2) and (b) *o*-phenylenediamines (1) and arylglyoxals/glyoxylic acids (4).

In continuation of our interest in the development of green and sustainable methodologies for nitrogen- and sulfur-based heterocyclic scaffolds,^97–99^ we herein demonstrate an SSMR strategy for the synthesis of 2-arylquinoxalines (3). This approach involves (a) various *o*-phenylenediamines (1) and different α-bromoketones (2), as well as (b) *o*-phenylenediamines (1) and a diverse set of arylglyoxals/glyoxylic acids (4). The reactions proceed under catalyst- and solvent-free conditions within a short time span (1–13 min), as illustrated in Scheme 1. Further, the photophysical and electrochemical properties of the synthesized 2-arylquinoxalines (3) have been investigated.

## Results and discussion

### Optimization and substrate scope

Considering the increasing interest in the development of green synthetic strategies for the synthesis of quinoxaline derivatives, it is aimed to develop a simple, rapid, highly efficient, scalable, economic and environmentally benign method for the synthesis of 2-arylquinoxalines (3). To achieve the goal, various green strategies have been applied on the model substrates, *o*-phenylenediamine (1a) (2.0 mmol) and 4-chlorophenacyl bromide (2a) (2.0 mmol) for the synthesis of 2-(4-chlorophenyl)quinoxaline (3a). For this purpose, initially, the model substrates, 1a and 2a were ground together in the presence of TiO_2_ nanotubes (TNTs) under solvent-free reaction conditions at RT for 10 min using grindstone chemistry (GSC). This resulted in a low yield (30%) of 2-(4-chlorophenyl)quinoxaline (3a) (Table 1, entry 1). Further, the same reaction was repeated by solvent-drop grinding (SDG) method using TNTs as catalyst in a variety of solvents (two drops) like water, ethanol, isopropanol and acetone at RT for 15 min. From this study, it was observed that the reaction proceeded partially and afforded very low to moderate yields of 3a that ranged from 15% to 45% (Table 1, entries 2–5). After that, the same reaction was performed under ultrasound irradiation (USI) in the presence of TNTs in different solvents (2.0 mL) like water, ethanol, isopropanol and acetone at 45 °C for 40–60 min. The study reveals that reaction proceeded smoothly in ethanol and afforded 75% yield of 3a (Table 1, entry 7). The other solvents, water, isopropanol and acetone provided low to moderate yields of product 3a (Table 1, entries 6, 8 & 9). Further, to improve the yield of 3a, the mixture of model substrates, 1a and 2a was melted at 55–60 °C by using SSMR strategy under solvent-free reaction conditions. To our delight, the target product 3a was obtained in almost quantitative yield (99%) within a very short period of time (1.0 min) (Table 1, entry 10). From the optimization study of the reaction, it was concluded that the SSMR strategy was the best green method as compared with other green methodologies such as GSC, SDG and USI in giving maximum yield (99%) of product 3a in 1.0 min at 55–60 °C.

**Table 1:** Optimization of reaction conditions^a^

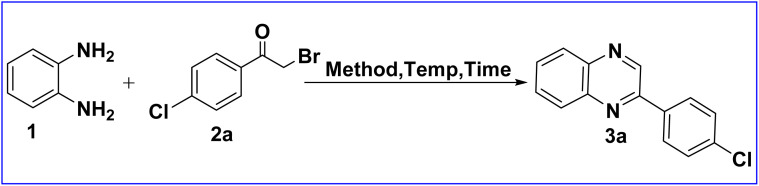						
Entry	Conditions			Temp.	Time (min)	Isolated yield^f^ (%)
	Solvent	Catalyst (10 mg)	Method			
1	Solvent-free	TNTs	GSC^b^	RT	10	30
2	Water (2 drops)	TNTs	SDG^c^	RT	15	15
3	Ethanol (2 drops)	TNTs	SDG^c^	RT	15	45
4	Isopropanol (2 drops)	TNTs	SDG^c^	RT	15	30
5	Acetone (2 drops)	TNTs	SDG^c^	RT	15	25
6	Water (2.0 mL)	TNTs	USI^d^	45 °C	60	20
7	Ethanol (2.0 mL)	TNTs	USI^d^	45 °C	40	75
8	Isopropanol (2.0 mL)	TNTs	USI^d^	45 °C	40	55
9	Acetone (2.0 mL)	TNTs	USI^d^	45 °C	40	30
**10**	**—**	**—**	**SSMR** e	**55–60 °C**	**1**	**99**

a Reaction conditions: *o*-phenylenediamine (1a) (2.0 mmol) and 4-chlorophenacyl bromide (2a) (2.0 mmol), reaction performed using green methods.

b GSC at RT in the presence of TNTs under solvent-free conditions.

c SDG in the presence of TNTs at RT.

d USI in the presence of TNTs at 45 °C in different solvents.

e SSMR at 55–60 °C under catalyst and solvent-free conditions.

f Isolated yields.

Having above well optimized reaction conditions, the scope and generality of the developed SSMR strategy was applied for the synthesis of a series of 2-arylquinoxaline derivatives (3) by using different *o*-phenylenediamines (1) and various α-bromoketones (2). The obtained results are summarized in Table 2. *o*-Phenylenediamine (1a) underwent the reaction with simple phenacyl bromide (2a) to obtain the desired product, 3a in excellent yield (99%). *o*-Phenylenediamine (1a) showed excellent reactivity with α-bromoketones bearing deactivating groups, 4-Cl (2a), 3-Cl (2b), 4-F (2c), 4-Br (2d), 4-NO_2_ (2f) and 4-CN (2g) at different positions of phenyl ring afforded the desired products, 3a, 3b, 3c, 3d, 3f and 3g in all most quantitative yields. *o*-Phenylenediamine (1a) also exhibited excellent reactivity with α-bromoketones containing activating methoxy group (4-OCH_3_ (2e)) at 4^th^ position of the phenyl ring gave 99% yield of 3e in less than 1 min. *o*-Phenylenediamine (1a) reacted well with 2-bromo-4′-phenylacetophenone (2h) to give 3h in excellent yield (97%). Similarly, 4-nitrobenzene-1,2-diamine (1b) displayed good reactivity with α-bromoketones bearing deactivating groups, 4-Cl (2a), 3-Cl (2b), 4-F (2c), 4-Br (2d), 4-NO_2_ (2f) and 4-CN (2g) at different positions of phenyl ring provided the corresponding products, 3i, 3j, 3k, 3l, 3n and 3o in excellent yields. 4-Nitrobenzene-1,2-diamine (1b) also showed excellent reactivity with α-bromoketones containing activating methyl group (4-CH_3_ (2i)) at 4^th^ position of the phenyl ring gave 98% yield of 3m. 4-Nitrobenzene-1,2-diamine (1b) underwent the reaction with 2-bromo-4′-phenylacetophenone (2h) to obtain 3p in good yield (96%). Further, 4,5-dimethylbenzene-1,2-diamine (1c) reacted well with α-bromoketones bearing deactivating groups, 4-F (2c), 4-Br (2d), 4-NO_2_ (2f) and 4-CN (2g) at different positions of phenyl ring gave the desired products, 3q, 3r, 3t and 3u in very good yields. 4,5-Dimethylbenzene-1,2-diamine (1c) underwent the reaction with 4-methoxyphenacylbromide (2e) to afford 3s in 98% yield. 4,5-dimethylbenzene-1,2-diamine (1c) also exhibited good reactivity with 2-bromo-4′-phenylacetophenone (2h) provided 3v in excellent yield (97%) in 12 min. 4-Fluorobenzene-1,2-diamine (1d) also reacted well with 4-methoxyphenacylbromide (2e) to provide the corresponding product, 3w in excellent yield (98%). From this study, it was concluded that the substrates with activating and deactivating groups on aromatic ring of both 1,2-diamines (1) and α-bromoketones (2) proceeded well to afford the desired products in excellent to quantitative yields under the optimized reaction conditions. The results indicate that the nature of substituents, steric hindrance and molecular dimension of the reactants play a very minimal role in the rate determining and yields of the products obtained in the established SSMR strategy.

**Table 2:** Synthesis of a series of 2-arylquinoxalines (3) from 1,2-diamines (1) and α-bromoketones (2)^a^

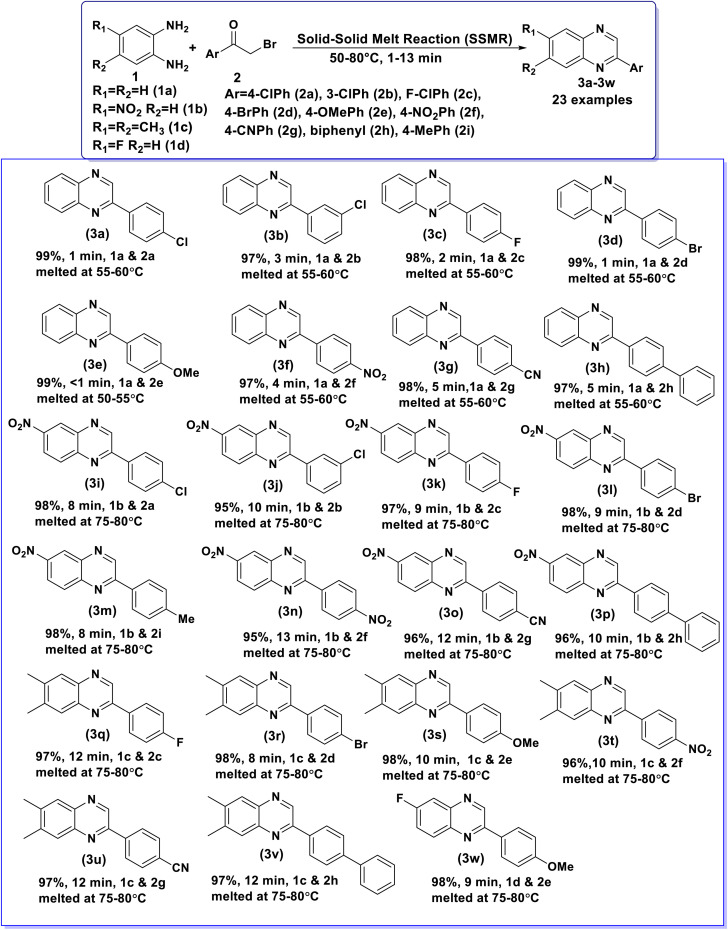

a Reaction conditions: 1 (2.0 mmol) and 2 (2.0 mmol), SSMR under catalyst and solvent-free conditions, 50–80 °C, 1–13 min.

Encouraged by the excellent preliminary results, the developed SSMR strategy was further extended to *o*-phenylenediamines (1a,1b and 1e and a diverse series of aryl glyoxals (4a–f), along with phenylglyoxylic acid (4g), and 2-thiopheneglyoxylic acid (4h). To optimize the reaction conditions for the synthesis of 2-(3,4-dimethoxyphenyl)quinoxaline (3x), a control experiment was conducted using *o*-phenylenediamine (1a, 2.0 mmol) and 3,4-dimethoxyphenylglyoxal (4a, 2.0 mmol) as model substrates. Remarkably, under solvent-free conditions, the mixture of 1a and 4a melted at 60–65 °C and furnished the target product 3x in an almost quantitative yield (99%) within 1.0 minute. This optimized procedure was subsequently applied to the synthesis of a wide range of 2-substituted quinoxalines (3y–3aj) derived from *o*-phenylenediamines (1a,b and 1e) and the aforementioned arylglyoxals (4a–f), phenylglyoxylic acid (4g), and 2-thiopheneglyoxylic acid (4h). The summarized results are presented in Table 3.

**Table 3:** Synthesis of a library of 2-arylquinoxalines (3) from 1,2-diamines (1) and arylglyoxals/glyoxylic acids (4)^a^

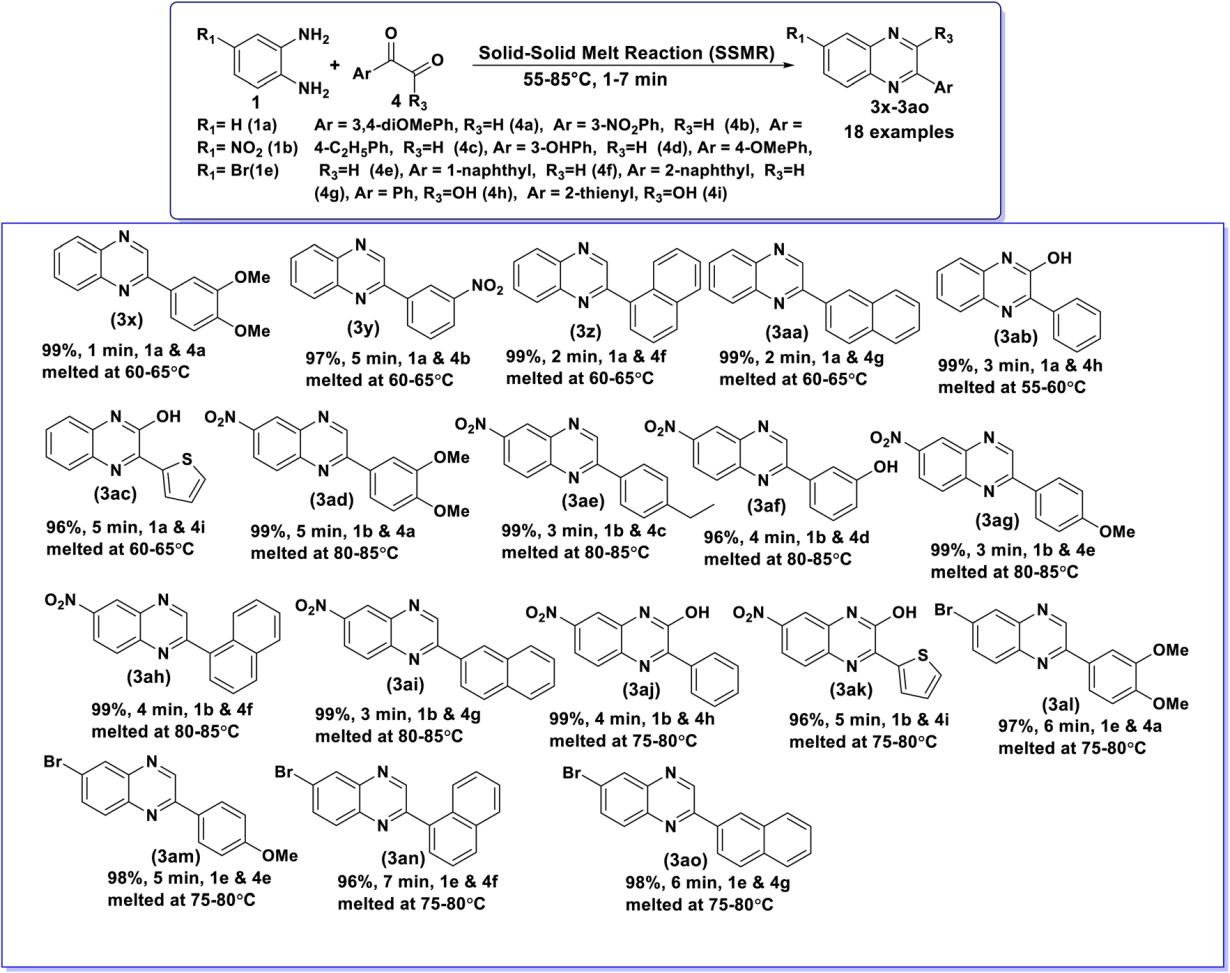

a Reaction conditions: 1 (2.0 mmol) and 4 (2.0 mmol), SSMR under catalyst and solvent-free conditions, 55–85 °C, 1–7 min.

The reaction of *o*-phenylenediamine (1a) with phenylglyoxals bearing both electron-donating (3,4-diOMe, 4a) and electron-withdrawing (3-NO_2_, 4b) substituents afforded the corresponding quinoxalines 3x and 3y in excellent yields (99% and 98%, respectively). Furthermore, *o*-phenylenediamine (1a) reacted smoothly with 1-naphthylglyoxal (4e) and 2-naphthylglyoxal (4f) to give the desired products 3z and 3aa in good yields. Notably, 1a also exhibited high reactivity with phenylglyoxylic acid (4g) and 2-thiopheneglyoxylic acid (4h), providing the corresponding quinoxalines 3ab and 3ac in excellent yields. Similarly, 4-nitro-1,2-phenylenediamine (1b) showed good reactivity with phenylglyoxals containing various substituents such as 3,4-diOMe (4a), 4-Et (4c), 3-OH (4d), and 4-OMe (4e), affording the respective quinoxalines 3ad, 3ae, 3af and 3ag in excellent yields. The reactions of 1b with 1-naphthyl glyoxal (4f) and 2-naphthyl glyoxal (4g) proceeded efficiently to produce 3ah and 3ai in good yields. Moreover, 1b reacted well with phenylglyoxylic acid (4h) and 2-thiopheneglyoxylic acid (4i) to afford 3aj and 3ak, also in good yields. 4-Bromo-1,2-phenylenediamine (1e) also underwent the reaction with phenylglyoxals containing 3,4-diOMe (4a) and 4-OMe (4e) groups on the phenyl ring provided the respective quinoxalines 3al and 3am in excellent yields. Further, 1e reacts with 1-naphthylglyoxal (4f) and 2-naphthyl glyoxal (4g) to provide 3an and 3ao in good yields. The study reveals that, regardless of the substituents on the phenyl ring of arylglyoxals/glyoxylic acids (4) and 1,2-diamines (1), the SSMR reaction strategy afforded excellent yields of the desired products (3).

### Probable mechanism

**Scheme 2:**
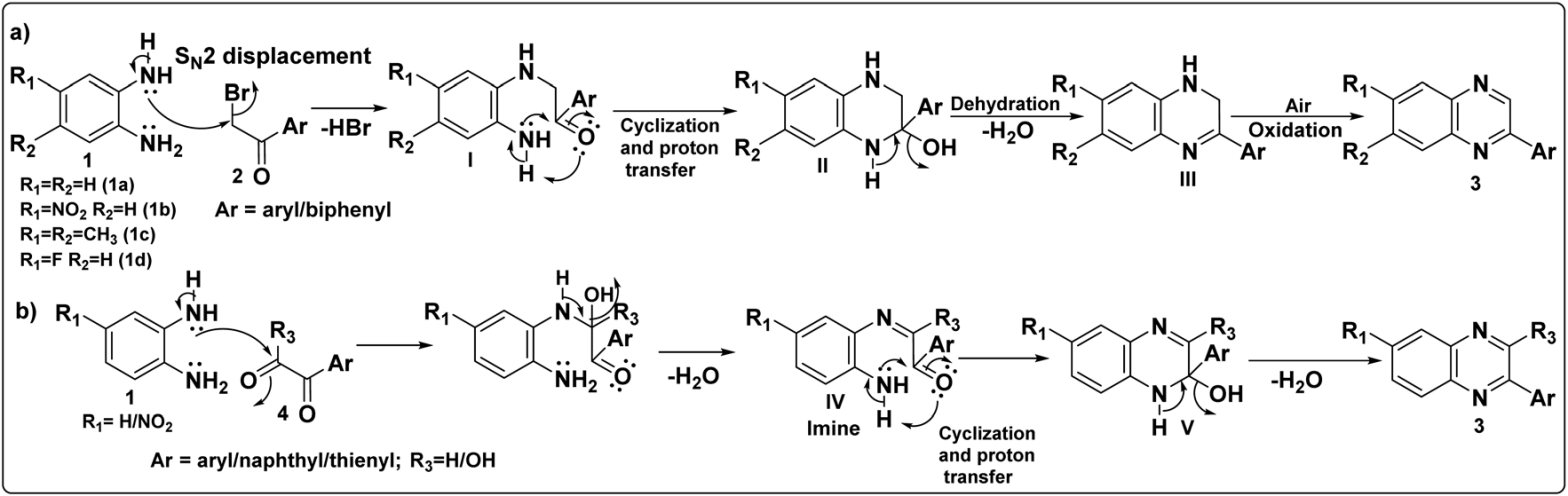
A probable mechanism for the synthesis of 2-arylquinoxalines (3) from (a) *o*-phenylenediamines (1) and α-bromoketones (2) and (b) *o*-phenylenediamines (1) and arylglyoxals/glyoxylic acids (4).

The plausible mechanisms for the formation of 2-arylquinoxalines (3) from (a) *o*-phenylenediamines (1) and α-bromoketones (2) and (b) *o*-phenylenediamines (1) andarylglyoxals/glyoxylic acids (4) are illustrated in Scheme 2. The mechanistic pathway of these transformations have also been reported in the literature.^35,75,85^

As exemplified in Scheme 2a, the mechanism is proposed to initiate *via* an S_N_2 attack by one of the amino groups of *o*-phenylenediamines (1) on the α-carbon of α-bromoketones (2) leading to the formation of an intermediate, 2-((2-aminophenyl)amino)-1-arylethan-1-one (I). Subsequently, this intermediate undergoes intramolecular cyclization through a nucleophilic addition of the second amino group to the carbonyl carbon, followed by a proton transfer to form an intermediate, 2-aryl-1,2,3,4-tetrahydroquinoxalin-2-ol (II). Finally, intermediate (II) undergoes dehydration to form 2-aryl-1,2-dihydroquinoxaline (III) which undergoes rapid oxidative aromatization under ambient air to afford the desired 2-arylquinoxalines (3).

To further support the mechanistic pathway, a control experiment was conducted using *o*-phenylenediamine (1a) and 4-bromophenacyl bromide (2d) under a nitrogen atmosphere at 55–60 °C. The reaction was carefully monitored to detect the formation of the proposed intermediates, I and III. The samples of the crude reaction mixture were collected at very short time intervals (25–30 s, sample 1; 40–45 s, sample 2) and immediately analyzed by mass spectrometry. The corresponding mass spectra for samples 1 and 2 are provided in the SI (Fig. S36 and S37).

The mass analysis of sample 1 showed a prominent peak at *m*/*z* 305 ([M + H]^+^), corresponding to intermediate I, along with a minor peak at *m*/*z* 289.3 ([M + H + 2]^+^), indicating the formation of intermediate III. In sample 2, the relative intensity of the peak at *m*/*z* 289.1 ([M + H + 2]^+^) increased significantly, while the peak at *m*/*z* 305.1 ([M + H]^+^) decreased, inferring the progression of the reaction from intermediate I to III. Additionally, a low-intensity peak at *m*/*z* 287.1 ([M + H + 2]^+^) was attributable to the final product (3d). These observations provide experimental support for the formation of key intermediates I & III and are consistent with the proposed reaction pathway, including the rapid oxidative aromatization step that occurs likely upon exposure to ambient air.

As illustrated in Scheme 2b, the reaction is assumed to proceed through a simple condensation between the aldehyde/carboxylic acid group of the arylglyoxals/glyoxylic acids (4) and one of the amino groups of *o*-phenylenediamine (1), forming the corresponding imine intermediate (IV). This imine then undergoes intramolecular cyclization *via* nucleophilic attack of the second amino group on the carbonyl carbon, followed by proton transfer to obtain the intermediate (V). Finally, dehydration of this intermediate affords the target 2-arylquinoxaline derivatives (3).

### Scalability

**Fig. 2:**
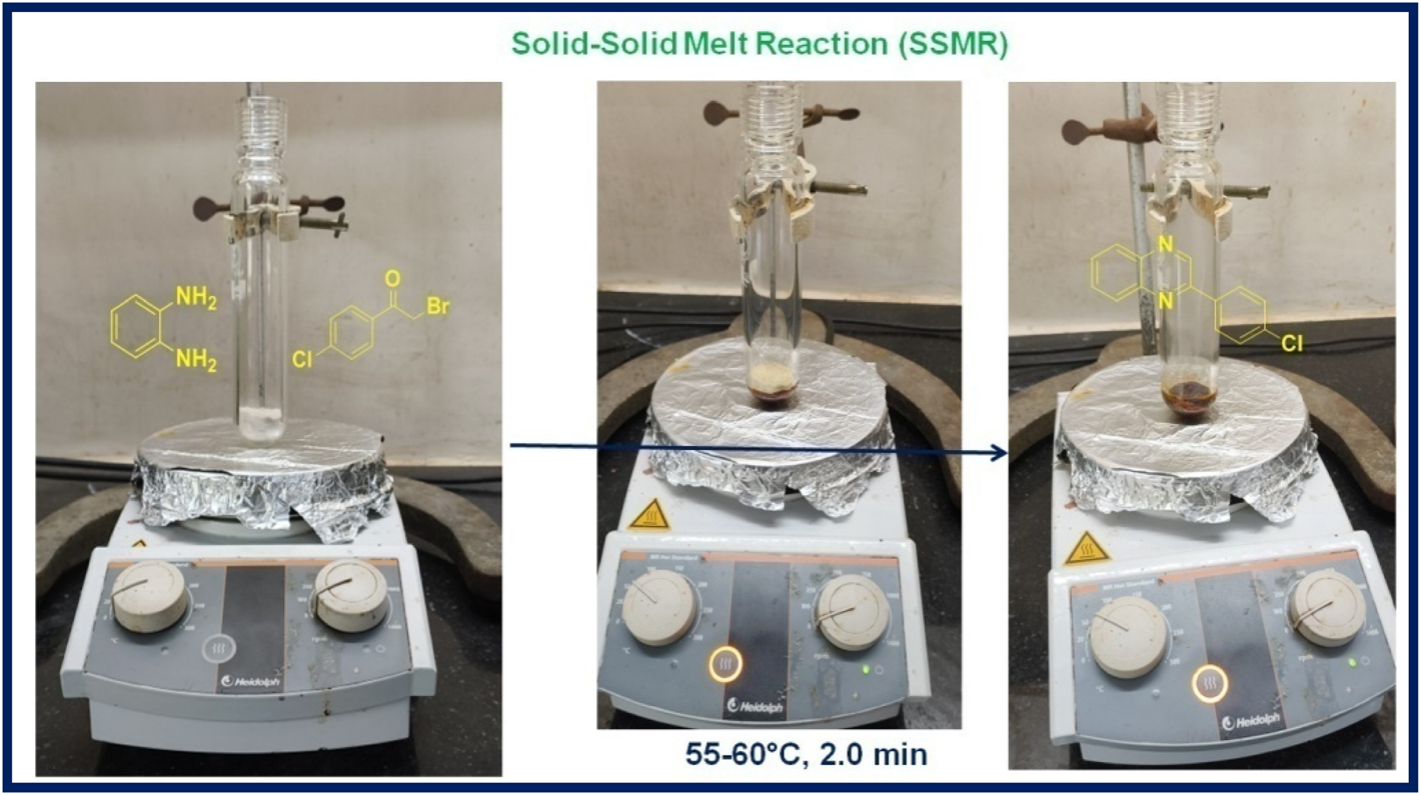
Gram scale synthesis of 2-(4-chlorophenyl)quinoxaline (3a).

Further, to check the possibility of scalability of the developed SSMR strategy, a gram-scale reaction has been performed between the model substrates, *o*-phenylenediamine (1a) (10.0 mmol, 1.08 g) and 4-chlorophenacyl bromide (2a) (10.0 mmol, 2.32 g), the corresponding product, 2-(4-chlorophenyl)quinoxaline (3a) was formed in 98% yield (2.35 g) (Fig. 2). From this study, it has been concluded that the established method is economic and most viable for industrial applications.

#### Green chemistry metrics

The green chemistry parameters of compounds 3a and 3x were evaluated using established metrics reported in the literature.^100,101^ The calculated values are presented in Table 4. Key green chemistry indicators including the E-factor, process mass intensity (PMI) and carbon efficiency were found to be close to their ideal values. Detailed calculations are provided in the SI. These results indicate that the developed methodologies are highly consistent with the principles of green chemistry.

**Table 4:** Green chemistry metrics for the compounds 3a and 3x

Entry	Parameters	Calculated values for compounds		Ideal value
		3a	3x	
1	Atom economy^a^ (AE) (%)	70.6	88.1	100
2	Carbon efficiency^b^ (CE) (%)	100	100	100
3	E-factor^c^ (*E*)	0.92	0.59	0
4	Process Mass Intensity^d^ (PMI)	1.92	1.59	1
5	Curzon's Reaction Mass Efficiency^e^ (Curzon's RME) (%)	70	87.2	100
6	Generalized Reaction Mass Efficiency^f^ (gRME) (%)	52	62.9	100

a AE (%) = 100 (GMW of product/sum of GMWs of reactants).

b CE (%) = [amount of carbon in product/total carbon present in reactants] × 100 = [no. of moles of product × no. of carbons in product/(moles of 1 × carbons in 1 + moles of 2 × carbons in 2)] × 100.

c *E* = total input mass (^m^inputs) − mass of target product (^m^3) − mass of recovered materials/mass of target product (^m^3).

d PMI = (^m^inputs − mass of recovered materials)/^m^3 (or) 1 + *E*.

e Curzon's RME (%) = 100 (mass of 3/mass of 1 + mass of 2) (or) 100 (yield × atom economy × 1/stoichiometric factor) (Stoichiometric Factor (SF) = 1).

f gRME (%) = 100 [^m^3/(^m^inputs − mass of recovered materials)] (or) 100[1/(1 + *E*)].

### Photophysical properties of 2-arylquinoxalines (3)

**Fig. 3:**
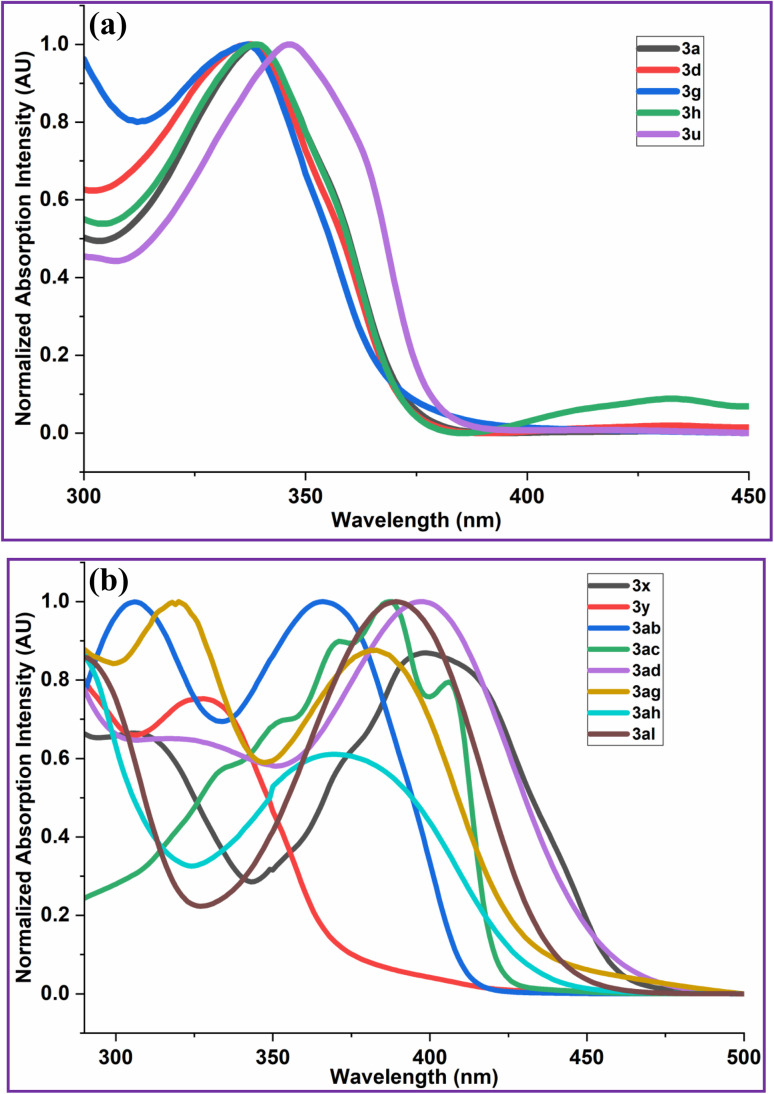
(a) Absorption spectra of 2-arylquinoxalines (3a, 3d, 3g, 3h & 3u) in DMSO (1.0 × 10^−10^ M). (b) Absorption spectra of 2-arylquinoxalines (3x, 3y, 3ab, 3ac, 3ad, 3ag, 3ah & 3al) in DMSO (1.0 × 10^−10^ M).

**Fig. 4:**
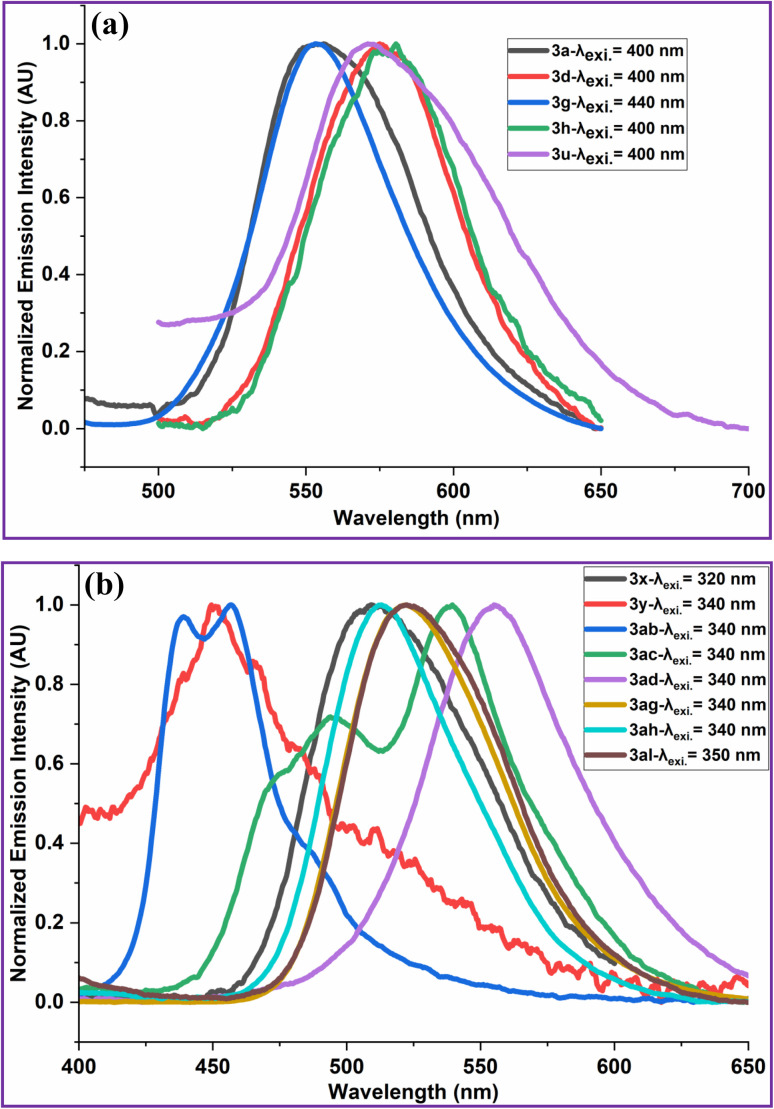
(a) Emission spectra of 2-arylquinoxalines (3a, 3d, 3g, 3h & 3u). Compounds 3a, 3d, 3h and 3u were excited at 400 nm and compound 3g was excited at 440 nm. (b) Emission spectra of 2-arylquinoxalines (3x, 3y, 3ab, 3ac, 3ad, 3ag, 3ah & 3al). Compound 3x was excited at 320 nm, compounds 3y, 3ab, 3ac, 3ad, 3ag and 3ah were excited at 340 nm and compound 3al was excited at 350 nm.

The photophysical properties of 2-arylquinoxalines (3) have been investigated from their absorption (Fig. 3) and emission (Fig. 4) spectra. The CIE colour coordinates and correlated colour temperature (CCT) values of the synthesized 2-arylquinoxalines (3) were determined from their respective in emission spectra (Fig. 4a and b).

**Fig. 5:**
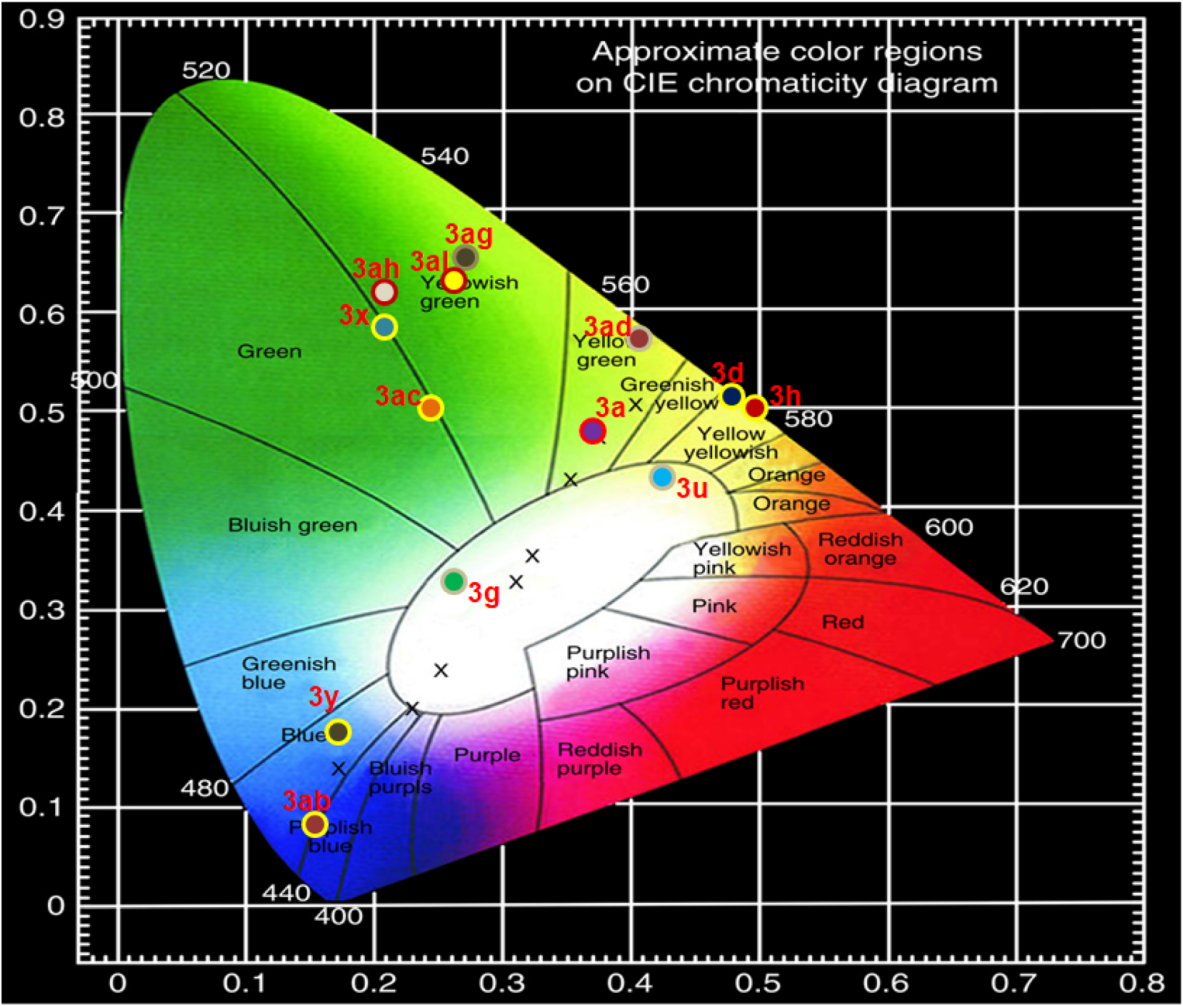
Chromaticity diagram of 2-arylquinoxalines (3).

The Commission Internationale de l'Éclairage (CIE) chromaticity coordinates (*x*, *y*) of compounds 3a, 3d, 3g, 3h, 3u, 3x, 3y, 3ab, 3ac, 3ad, 3ag, 3ah and 3al have been calculated from their emission spectra recorded at different excitation wavelengths using a well-established method.^102^ The results are summarized in Table 5 and Fig. 5.

**Table 5:** Photophysical properties of 2-arylquinoxalines (3)

Compd.	*λ*_abs_ a (nm)	*λ*_emi._ b (nm)	CIE coordinates		CCT (K)
			*x*	*y*	
3a	339	556	0.37	0.49	—
3d	337	578	0.48	0.51	—
3g	337	554	0.26	0.33	9861
3h	339	580	0.50	0.50	—
3u	347	575	0.42	0.46	3679
3x	308 & 401	508	0.21	0.59	—
3y	328	450	0.17	0.18	—
3ab	306 & 367	439 & 457	0.15	0.08	—
3ac	388	495 & 539	0.25	0.5	—
3ad	398	555	0.40	0.57	—
3ag	319 & 383	522	0.26	0.65	—
3ah	372	513	0.21	0.62	—
3al	390	523	0.26	0.63	—

a Absorption measured in DMSO (1.0 × 10^−10^ M).

b Emission measured in the solid state.

Analysis of the CIE chromaticity coordinates (*x*, *y*) revealed that the emission colour of the investigated compounds (3) could be effectively tuned from white to yellow, primarily governed by the nature of the substituents at the 2nd position of the quinoxaline core. Compound 3a, bearing a 4-chlorophenyl group at the 2-position, exhibited yellow-green light emission upon excitation at 400 nm. Similarly, compounds 3d (4-bromophenyl-substituted) and 3h (biphenyl-substituted) emitted yellow light when excited at 400 nm. Interestingly, the cyano-substituted derivatives 3g (4-cyanophenyl at the 2nd position of quinoxaline) and 3u (4-cyanophenyl at the 2nd position of 6,7-dimethylquinoxaline) displayed distinct white-light emissions. Upon excitation at 440 nm and 400 nm, respectively, compound 3g exhibited cold white light emission, whereas compound 3u produced warm white light emission. Compounds 3x (3,4-dimethoxyphenyl-substituted quinoxaline; *λ*_ex_ = 320 nm), 3ac (3-(thiophen-2-yl)quinoxalin-2-ol; *λ*_ex_ = 340 nm) and 3ah (1-naphthyl-substituted 6-nitroquinoxaline; *λ*_ex_ = 340 nm) showed green emission. In contrast, compounds 3y (3-nitrophenyl-substitutedquinoxaline) and 3ab (3-phenylquinoxalin-2-ol) emitted blue and purplish-blue light, respectively, upon excitation at 340 nm. Furthermore, compound 3ad, containing a 3,4-dimethoxyphenyl group at the 2-position of the 6-nitroquinoxaline ring, exhibited yellow-green emission when excited at 340 nm. Compounds 3ag (4-methoxyphenyl-substituted 6-nitroquinoxaline) and 3al (3,4-dimethoxyphenyl-substituted 6-bromoquinoxaline), excited at 340 nm and 350 nm, respectively, displayed yellowish-green emission.

These results were further supported by the correlated colour temperature (CCT) analysis.

The CCT values have been determined by using the McCamy empirical formula (eqn (1)) [ref. 103] for the characterization of the colour emission and its temperature.

1 CCT = −449*n*^3^ + 3525*n*^2^ − 6823*n* + 5520.33

where *n* = (*x* − *x*_e_)/(*y* − *y*_e_) and the chromaticity epicenter is at *x*_e_ = 0.3320 and *y*_e_ = 0.1858.

The high CCT value of 3g (9861 K) confirming the cold white light emission region and the lower CCT value of 3u (3679 K) indicating the warm white light emission region (Table 3).

The external quantum efficiencies (EQEs) of the multi-colour emissive title compounds 3d, 3g, 3u, 3x, and 3ab in the solid-state were determined using a commercially available cerium-doped yttrium aluminum garnet (YAG:Ce^3+^) phosphor manufactured by CREE as the standard reference. To authentically match their prospective integration into white-light-emitting diode (WLED) architectures, the EQEs of both the reference phosphor and the quinoxaline derivatives were rigorously evaluated as finely dispersed powders uniformly coated on quartz slides. Strict adherence to identical experimental protocols encompassing excitation conditions, detection geometry, and instrumental parameters confirmed exceptional reproducibility and analytical reliability.

In the solid-state, the EQEs (%) for compounds 3d, 3g, 3u, 3x and 3ab were determined to be 0.99, 2.13, 1.43, 0.40 and 0.52, respectively. Complementary solid-state photophysical investigations of the synthesized quinoxalines revealed highly tunable emission profiles spanning purplish-blue to vibrant yellow wavelengths, with the emission colour profoundly modulated by the electronic nature and steric demands of substituents at the 2-position of the quinoxaline core. Strikingly, compounds 3g and 3u exhibited cold-white and warm-white light emissions, respectively, highlighting their exceptional capacity to generate high-quality, broadband white light directly in the condensed phase without the need for additional dopants or complex host matrices. Moreover, the frontier molecular orbital (HOMO–LUMO) energy levels of these luminophores closely mirror to those of established hole-transporting materials (HTMs), imparting them with dual functionality as both efficient emitters and charge transporters.

The present quinoxaline derivatives offer a compelling organic alternative to conventional rare-earth phosphors in phosphor-converted WLEDs due to their multi-colour solid-state emission with respectable quantum efficiencies and inherent hole-transporting capabilities. This work could significantly enhance luminous efficacy and tunable correlated colour temperature (CCT) while simultaneously lowering material costs and improving long-term operational stability under high-flux excitation. In the realm of next-generation displays, the tunable emission spectra and favorable charge-injection properties position these luminophores as ideal candidates for advanced emissive layers in organic light-emitting diodes (OLEDs) and micro-LED arrays, promising wider colour gamuts, superior energy efficiency, reduced power consumption, and enhanced device longevity attributes that are indispensable for high-fidelity visual technologies ranging from portable consumer electronics to large-format, high-resolution displays.

Collectively, the rationally engineered quinoxaline-based luminophores seamlessly integrate versatile multi-colour solid-state luminescence with effective hole-transport characteristics, establishing them as multifunctional materials poised to drive innovation across the broad landscape of organic optoelectronics.

### Electrochemical properties of 2-arylquinoxalines (3)

Cyclic voltammetry (CV) was utilized to investigate the charge-transport characteristics of the synthesized compounds (3), including hole transport, electron transport and ambipolar behavior. Electrochemical measurements were performed in DMSO at a concentration of 1.0 × 10^−5^ M, using tetrabutylammonium hexafluorophosphate (Bu_4_NPF_6_, 0.1 M) as the supporting electrolyte. A standard three-electrode configuration was employed, consisting of an Ag/Ag^+^ (saturated KCl) reference electrode, a platinum disk working electrode and a platinum wire as the counter electrode. All voltammograms were recorded at a scan rate of 100 mV s^−1^ over a potential window ranging from −0.2 to 1.6 V.

**Fig. 6:**
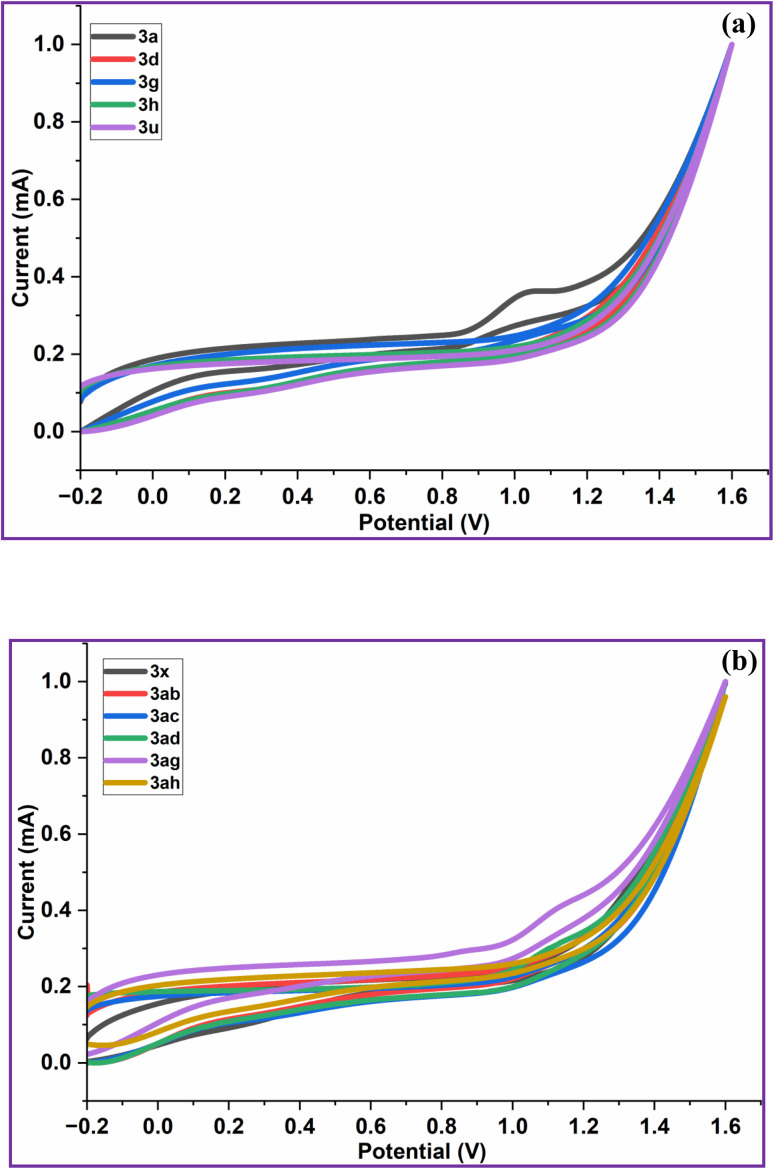
(a) Cyclic voltammograms of 2-arylquinoxalines (3a, 3d, 3g, 3h & 3u) in DMSO (1.0 × 10^−5^ M) at a scan rate of 100 mV s^−1^. (b) Cyclic voltammograms of 2-arylquinoxalines (3x, 3ab, 3ac, 3ad, 3ag & 3ah) in DMSO (1.0 × 10^−5^ M) at a scan rate of 100 mV s^−1^.

The highest occupied molecular orbital (HOMO) energy levels were estimated from the oxidation onset potentials according to the relation, *E*_HOMO_ = −[*E*_ox (onset)_ − *E*_FOC_ + 4.8; (*E*_FOC_ = 0.38 eV)] and the lowest unoccupied molecular orbital (LUMO) energies were derived using the optical band gap (*E*^opt^_g_), calculated from the absorption spectra (Fig. 3a and b; *E*^opt^_g_ = 1240/*λ*_edge_), following the expression, *E*_LUMO_ = (*E*_HOMO_ + *E*^opt^_g_). All examined compounds (3a, 3d, 3g, 3h, 3u, 3x, 3ab, 3ac, 3ad, 3ag & 3ah) displayed quasi-reversible redox features in their cyclic voltammograms (Fig. 6a and b) and the extracted electrochemical parameters are compiled in Table 6. As summarized therein, the HOMO and LUMO energy levels of the synthesized quinoxalines (3) fall within the ranges of −5.63 to −5.52 eV and −2.92 to −2.23 eV, respectively. These values are in close agreement with those reported hole-transport materials (HTMs), such as *N*,*N*′-bis(metatolyl)-*N*,*N*′-diphenylbenzidine (TPD, HOMO/LUMO −5.5/−2.3 eV), *N*,*N*′-di-(naphthalen-1-yl)-*N*,*N*′-diphenylbenzidine (NPB; HOMO/LUMO -5.5/-2.4 eV), poly(9,9-dioctylfluorene-*alt*-bithiophene) (F8T2; HOMO/LUMO −5.5/−3.1 eV) and 4,4′-[bis-((4-di-*n*-hexylamino)benzylideneamino)]stilbene (DHABS; HOMO/LUMO −5.6/−2.7 eV)^104,105^ (Table 6). Among the literature-reported HTMs, TPD, NPB, and F8T2 are commonly used as hole-transporting layers (HTLs) in OLED devices. Notably, the HOMO levels of compounds (3) are lower than the air-oxidation threshold levels (≈−5.2 eV), indicating enhanced resistance to oxidative degradation under ambient conditions.^106,107^

**Table 6:** Electrochemical properties of 2-arylquinoxalines (3) and their HOMO–LUMO energy levels compared with literature-reported HTMs

Compd.	*λ*_edge_ (nm)	*E* ^opt^_g_ (eV)	*E* ^ox^_(onset)_	*E*_HOMO_ (eV)	*E*_LUMO_ (eV)	Commercial name of the HTM	*E*_HOMO_ (eV)	*E*_LUMO_ (eV)	Ref.
3a	379	3.27	1.142	−5.56	−2.29	TPD/NPB	−5.5/−5.5	−2.3/−2.4	105
3d	377	3.28	1.142	−5.56	−2.28	TPD/NPB	−5.5/−5.5	−2.3/−2.4	105
3g	376	3.29	1.107	−5.52	−2.23	TPD/NPB	−5.5/−5.5	−2.3/−2.4	105
3h	376	3.29	1.110	−5.53	−2.24	TPD/NPB	−5.5/−5.5	−2.3/−2.4	105
3u	384	3.23	1.164	−5.58	−2.35	TPD/NPB	−5.5/−5.5	−2.3/−2.4	105
3x	463	2.67	1.139	−5.56	−2.89	F8T2	−5.5	−3.1	105
3ab	416	2.98	1.208	−5.63	−2.65	DHABS	−5.6	−2.7	105
3ac	425	2.91	1.195	−5.61	−2.70	DHABS	−5.6	−2.7	105
3ad	473	2.62	1.120	−5.54	−2.92	F8T2	−5.5	−3.1	105
3ag	439	2.82	1.104	−5.52	−2.70	DHABS	−5.6	−2.7	105
3ah	440	2.81	1.189	−5.61	−2.80	DHABS	−5.6	−2.7	105

### Computational study

The computational studies were carried out by using the Gaussian 09W suite at the B3LYP/6-311+G(d,p) level of theory,^108^ to predict the electronic structure of the studied compounds. To gain deeper insight into the photophysical behavior, electronic transitions particularly the frontier molecular orbitals (HOMO and LUMO) were analyzed for representative compounds, 3ad, 3ag, and 3ah in the solution phase employing the time-dependent density functional theory (TD-DFT) approach.

**Fig. 7:**
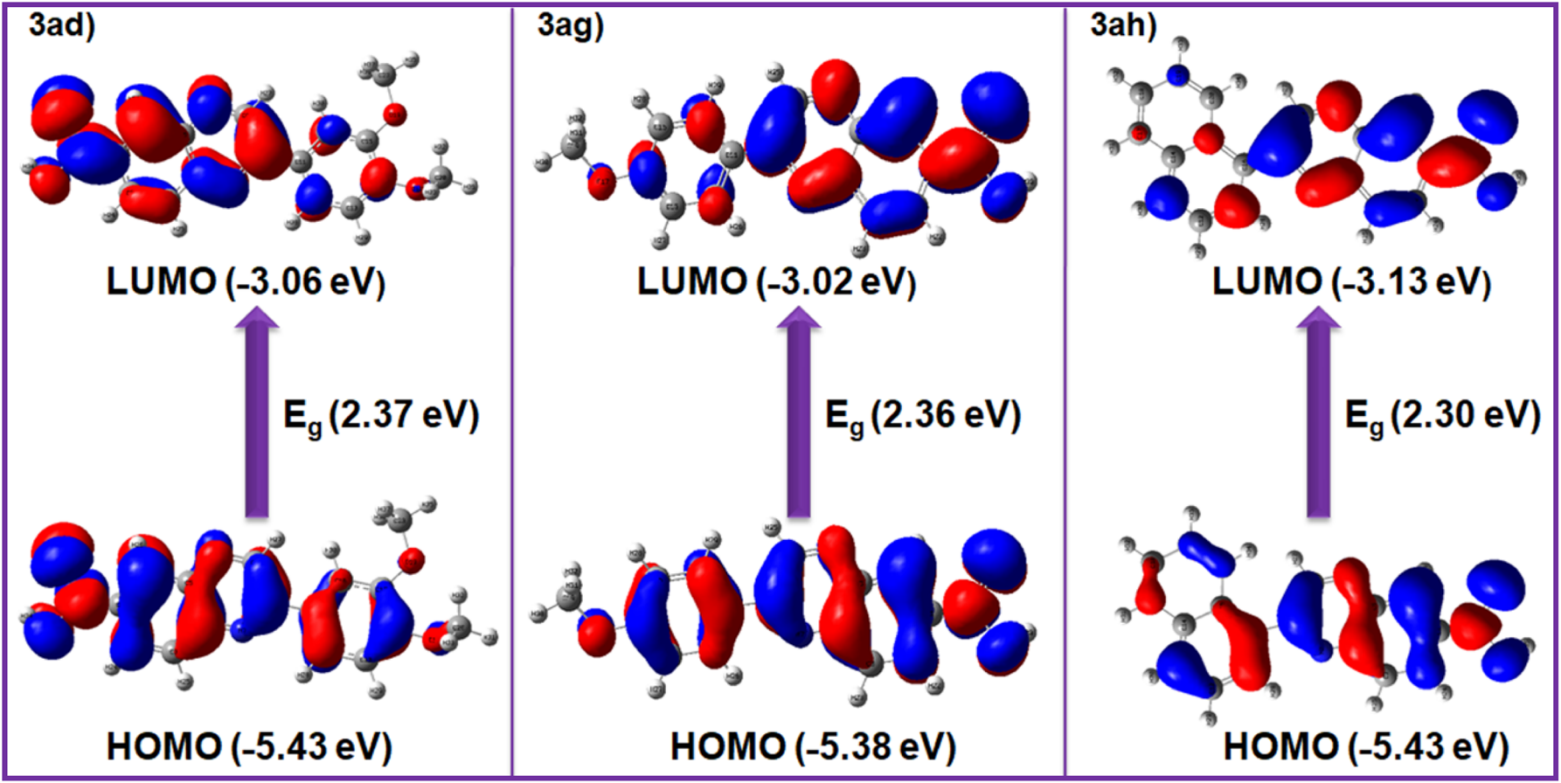
The HOMO/LUMO distributions of 3ad, 3ag and 3ah.

The spatial distribution of the HOMO and LUMO orbitals for these model systems is illustrated in Fig. 7, providing a clear visualization of electron density migration upon excitation. Quantitatively, the calculated HOMO/LUMO energy levels were found to be −5.43/−3.06 eV for 3ad, −5.38/−3.02 eV for 3ag, and −5.43/−3.13 eV for 3ah, corresponding to band gap (*E*_g_) values of 2.37, 2.36, and 2.30 eV, respectively. These relatively narrow band gaps suggest favorable electronic delocalization and potential applicability in optoelectronic systems.

Importantly, these theoretical results exhibit good agreement with the experimentally derived values obtained from cyclic voltammetry measurements. The experimental HOMO/LUMO energies for 3ad (−5.54/−2.92 eV), 3ag (−5.52/−2.70 eV) and 3ah (−5.61/−2.80 eV) correspond to energy gaps (*E*_g_) of 2.62, 2.82, and 2.81 eV, respectively (Table 6). Although slight deviations are observed arising probably from solvent effects, methodological approximations and experimental conditions the overall consistency validates the robustness of the computational model. The strong correlation between theoretical predictions and electrochemical data reinforces the reliability of the adopted DFT framework for probing electronic properties of such molecular systems.

### Comparative study of quinoxaline synthesis using selected reported methodologies

Table 7 summarizes a comparison between the present method and reported protocols for the synthesis of quinoxalines. Reported green energy-assisted methods, including mechanochemical, microwave, ultrasound, and photochemical approaches (Table 7, entries 1–14, 20–27) are efficient to provide moderate to excellent yields but they often require specialized equipment, longer reaction times, elevated temperatures, expensive catalysts, hazardous solvents, chromatographic purification, external energy sources. In addition, their scalability remains limited.^41–54,65–71^

**Table 7:** Comparative study of selected methods for the synthesis of quinoxalines

Entry	Method	Catalyst/irradiation	Solvent	Temp. (°C)	Time (min)	Purification	Yield (%)	Scalability	Ref.
**(I) Synthesis of 2,3-disubstitutedquinoxalines from 1,2-diamines and 1,2-dicarbonylcompounds**									
*(A) Green energy assisted methods*									
1	Homogenization (2 mm SS balls & 4000 rpm)	—	—	RT	3	Some products require column chromatography	91–99	Yes	41
2	Grinding	[EMIM]AlCl_4_	EtOH	RT	5–8	Column chromatography	45–83	—	42
3	Mechanochemical ball milling (600 rpm)	La(DS)_3_ and NaCl	—	RT	30	Column chromatography	72–99	—	43
4	Liquid assisted hand-grinding	—	EtOH	RT	10–30	Liquid products need column chromatography	80–98	—	44
5	Grinding	Nano-kaoline/BF_3_/Fe_3_O_4_	—	RT	10–75	Recrystallization (EtOH)	69–98	—	45
6	Grinding	Basic Al_2_O_3_	—	RT	10–25	Column chromatography	87–99	—	46
7	Microwave	Microwave irradiation	H_2_O (A)/AcOH (B)	230	60/10	Column chromatography	86–95/80–97	—	47
8^a^	Microwave	Microwave irradiation	Xylene	220	60	Column chromatography	70–96	Yes	48
9	Microwave	Microwave irradiation (100–220 W)	Glycerol	80–100	2–8	Column chromatography	83–95	—	49
10	Microwave	Microwave irradiation (400 W)	—	—	0.5–3	Crystallization (EtOAc and hexane (7 : 3))	80–90	—	50
11	Ultrasound	Ultrasound irradiation (150 W & 55 kHz)	EtOH and AcOH	RT	45–90	Column chromatography	90–98	—	51
12	Ultrasound	Ultrasound irradiation (750 W, 2000 J & 20% amplitude	H_2_O	RT	0.58–4.2	Recrystallization (ethanol/toluene)	86–99	—	52
13	Visible-light	Rose Bengal/CFL bulb irradiation (23 W)	CH_3_CN	RT	30–180	Column chromatography	75–93	—	53
14^b^	Visible-light	Irradiation with blue LED (30 W, 448 nm)	1,2-DCE	80	720	Column chromatography	27–99	Yes	54

*(B) Conventional methods*									
15	Magnetic stirring	—	Choline chloride/water	RT	5	Extraction with EtOAc	46–97	—	55
16	Magnetic stirring	Ca(IO_3_)_2_	EtOH	RT	3–20	Recrystallization (EtOH or MeOH/AcOH)	89–97	—	56
17	Magnetic stirring	Ni@Co_3_O_4_ nanocage	EtOH	30	15–20	—	85–100	—	57
18	Heating	CuO@g-C_3_N_4_	—	100	5–60	Column chromatography	71–90	Yes	58
19	Magnetic stirring	Gum Arabic	H_2_O : EtOH (1 : 4)	RT	36–1080	Recrystallization (EtOH)	55–98	—	59

**(II) Synthesis of 2-substitutedquinoxalines from 1,2-diamines and α-haloketones/methylketones**									
*(A) Green energy assisted methods*									
20	Mechanochemical ball milling (20 Hz & 6 balls) (3.0 mm)	TCCA, *p*-TSA & K_2_CO_3_	—	RT	600	Column chromatography	75–82	—	65
21	Mechanochemical ball milling (600 rpm)	La(DS)_3_ and NaCl	—	RT	30	Column chromatography	72–99	—	43
22	Microwave	Microwave irradiation	*t*-BuOH/CH_2_Cl_2_	100	15	Column chromatography	31–62	—	66
23	Microwave	Silica gel/Microwave irradiation	—	70–150	10–20	Column chromatography	70–85	—	67
24	Microwave	AgI, I_2_/microwave irradiation (300–350 W)	H_2_O–PEG-400 (1 : 2)	90–100	3–5	Preparative TLC	93–97	—	68
25	Microwave	Microwave irradiation		100–120	8	Recrystallization (95% EtOH)	37–95	—	69
26	Ultrasound	TMSCl/ultrasound irradiation (150 W & 55 kHz)	Glycerol–water	70–75	2.7–3	Recrystallization (aq. EtOH)	90–94	—	70
27	Visible light	K_2_CO_3_/CFL bulb irradiation (9 W)	DMSO & O_2_ pressure (2 atm)	RT	1440	Column chromatography	56–85	Yes	71

*(B) Conventional methods*									
28	Magnetic stirring	Cu(TfO)_2_	EtOH	50	480	Column chromatography	74–91	Yes	72
29	Magnetic stirring	Cu_0.5_Ni_0.5_ Fe_2_O_4_	EtOH	Reflux	30–55	Recrystallization (hot EtOH)	91–98	—	73
30	Magnetic stirring	Al_2_O_3_–ZrO_2_	DMF	RT	120	Recrystallization	51–92	—	74
31	Magnetic stirring	S_8_, oxalic acid	DMSO	80–110	960	Column chromatography	10–83	Yes	75
32	Heating	Nano-γ-Fe_2_O_3_–SO_3_H	—	120	60	Column chromatography	58–97	—	76
33	Magnetic stirring	Cu(0)/Al_2_O_3_	H_2_O	RT	7–80	Column chromatography	81–92	—	77
34	Magnetic stirring	—	H_2_O	80	120–180	Column chromatography	72–85	—	85
**35**	**SSMR**	**—**	**—**	**5**0**–85**	**1–13**	**Washing with EtOH**	**95–99**	**Yes**	**Present work**

a The reactants were (2-(3-oxoindolin-2-yl)-2-arylacetonitriles and 1,2-diamines.

b The reactants were benzoquinone, acetylene and 1,2-diamines.

Conventional methods (Table 7, entries 15–19, 28–34), on the other hand, frequently employ expensive metal-based nanocatalysts and provide low to good yields. These methods are associated with several limitations, including prolonged reaction times, use of toxic solvents, dependence on column chromatography for purification, limited scalability and environmental concerns.^55–59,72–77,85^

The present SSMR strategy (Table 7, entry 35) proceeds under catalyst-free and solvent-free conditions, affording excellent yields (95–99%) within a short time (1–13 min) at relatively low temperatures. The method avoids the need for specialized or costly equipment, rendering it both economically attractive and readily adaptable to routine laboratory practice. It further demonstrates gram-scale feasibility and a broad substrate scope, accommodating diverse α-bromoketones/arylglyoxals/glyoxylic acids and substituted *o*-phenylenediamines. Notably, the products are obtained in high purity without chromatographic purification. These features underscore the superiority of this protocol over existing methodologies.

## Conclusions

A solvent- and catalyst-free solid–solid melt reaction (SSMR) strategy has been developed as a green and sustainable approach for the synthesis of multi-colour emissive quinoxaline-based small organic fluorophores (QBSOFs, 3). The methodology enables the efficient construction of 2-arylquinoxalines (3) through reactions of *o*-phenylenediamines (1) with α-bromoketones (2) or arylglyoxals/glyoxylic acids (4). The protocol offers several key advantages, including (i) an environmentally benign and operationally simple process, (ii) elimination of cost-intensive and scale-limiting techniques such as microwave or ultrasonic irradiation, (iii) broad substrate scope with excellent functional-group tolerance, (iv) straightforward work-up procedure affording products in high purity and (v) excellent to near-quantitative yields (95–99%) within short reaction times. The feasibility of gram-scale synthesis further underscores the economic and industrial potential of this approach for the preparation of 2-arylquinoxalines (3). From a green chemistry perspective, key metrics such as E-factor, process mass intensity (PMI), and carbon efficiency approach ideal values, confirming that the developed methodology aligns with the principles of “benign by design.” The solid-state photophysical investigation of the synthesized quinoxalines revealed tunable emission spanning purplish blue to yellow light, which is strongly influenced by the nature of substituents at the 2-position of the quinoxaline core. Notably, compounds 3g and 3u exhibited cold white and warm white light emission, respectively. Additionally, the HOMO–LUMO energy levels of these luminophores are comparable to those of reported hole-transport materials (HTMs). These findings demonstrate that the synthesized quinoxaline-based luminophores combine multi-colour solid-state emission with effective hole-transporting characteristics, making them suitable candidates for a variety of applications in the field of organic optoelectronics.

## Experimental

### General information

All chemicals and solvents were commercially available and used without any further purification. The ^1^H NMR spectra were recorded on Bruker NMR spectrometer (400 MHz). Mass spectra were recorded on Shimadzu-LCMS-2010A mass spectrometer. The absorbance spectra were taken using a Shimadzu model UV-3100 UV-visible spectrophotometer. Emission spectra were recorded on Hitachi fluorescence spectrophotometer (F-2710). Reactions were monitored by thin layer chromatography (TLC) performed on 0.25 mm Merck silica gel plates and the developed plates were visualized under UV light. Millipore double distilled water was used for the work up process. α-Bromoketones are prepared according to the reported method.^109^

#### General procedure for the preparation of 2-arylquinoxalines (3)

A mixture of *o*-phenylenediamines (1, 2.0 mmol) and either α-bromoketones (2, 2.0 mmol) or arylglyoxals/glyoxylic acids (4, 2.0 mmol) was heated under melt conditions at 50–85 °C for 1–13 min. The progress of the reaction was monitored by TLC. Upon completion, the reaction mixture was allowed to cool to room temperature and the crude product was purified by washing with ethanol (1.5 mL) to afford the corresponding 2-arylquinoxaline (3) in pure form. Most of the synthesized 2-arylquinoxalines (3) are previously reported and have been structurally characterized.^65–85^

#### Gram-scale synthesis of 2-(4-chlorophenyl)quinoxaline (3a)

A mixture of *o*-phenylenediamine (1a, 10.0 mmol) and 4-chlorophenacyl bromide (2a, 10.0 mmol) was heated under melt conditions at 55–60 °C for 2 min. Reaction progress was monitored by TLC. After completion, the reaction mixture was cooled to room temperature, and the crude product was purified by washing with ethanol (5.0 mL) to yield pure 2-(4-chlorophenyl)quinoxaline (3a).

### Procedure adapted for quantum yield measurements

The standard reference (YAG:Ce^3+^) and the target multi-colour-emissive samples (3d, 3g, 3u, 3x, and 3ab) were finely ground to ensure homogeneity and uniformly deposited onto quartz slides, with coating thickness carefully optimized to afford low optical density (*A* < 0.1) at the excitation wavelength. An excitation wavelength of 460 nm was precisely aligned with the emission of blue LEDs routinely employed in commercial white-LED fabrication. Emission spectra were recorded under strictly identical conditions within an integrating-sphere setup, maintaining constant integration times, slit widths, and detector gains throughout. Absorbance values at the excitation wavelength were measured for both the reference and the samples. The fraction of absorbed light (*F*_abs,ref_ and *F*_abs,samples_) and the integrated emission intensities (*I*_ref_ and *I*_samples_) were precisely calculated from the respective spectra. Quantum yields (*Φ*_samples_) were then derived relative to the known value of the reference (*Φ*_ref_ = 0.76) using the following formula:
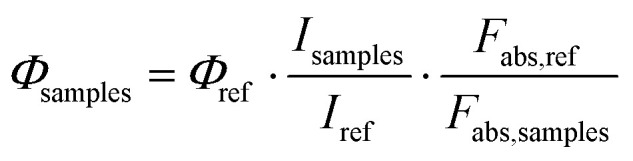
where, *I*_samples_ and *I*_ref_: integrated emission intensities of the samples (3d, 3g, 3u, 3x and 3ab) and reference, respectively. *F*_abs,samples_ and *F*_abs,ref_: fractions of light absorbed by the of the samples (3d, 3g, 3u, 3x and 3ab) and reference, respectively.

Because all measurements were performed in the solid-state configuration, the refractive index (*η*) was reasonably assumed to be equivalent across specimens, thereby eliminating the need for refractive-index correction terms and simplifying the comparative analysis.

## Conflicts of interest

The authors declare that they have no known competing financial interests or personal relationships that could have appeared to influence the work reported in this paper.

## Supplementary Material

RA-016-D6RA01059H-s001

## Data Availability

The data that supports the findings of this study are available in the supplementary information (SI) of this article. Supplementary information: physical & spectral data and copies of ^1^H-NMR, ^13^C-NMR and mass spectra of some of the 2-arylquinoxalines (3). See DOI: https://doi.org/10.1039/d6ra01059h.
