# Supplementary material for: Catalyst and solvent-free solid–solid melt synthesis of multi-colour emissive 2-functionalized quinoxaline-based fluorophores
Source: RSC Adv. 2026 Apr 24;16(24):21676–92. doi: 10.1039/d6ra01059h (PMC13107942; doi:10.1039/d6ra01059h)
Supplement: RA-016-D6RA01059H-s001 [file RA-016-D6RA01059H-s001.pdf]

Supplementary Information (SI) for RSC Advances.
This journal is © The Royal Society of Chemistry 2026

……. Supplementary Information…….

Catalyst and solvent-free solid-solid melt synthesis of multi-colour emissive 2-functionalized quinoxaline-based fluorophores

Sai TejaTalari,[a†] Rama Mohana Reddy Sirigireddy, [a†] Siva Dakshayani Vadanapalli,[a] Mohan Das Dasappa,[a] Sultana Shaik,[a] Venkatramu Vemula,[b] Eethamukkala Ubba,[c] Ramamohana Reddy Maddike,[d] Haranath Divi,[e] Chinna Gangi Reddy Nallagondu*[a]

[a]Green & Sustainable Synthetic Organic Chemistry and Optoelectronics Laboratory, Department of Chemistry, Yogi Vemana University, Kadapa-516005, Andhra Pradesh, India.

[b]Department of Physics, Yogi Vemana University, Kadapa-516 005, Andhra Pradesh, India.

[c]Department of Chemistry, Andhra Kesari University, Ongole-523 001, Andhra Pradesh, India.

[d] Lincoln University College, Petaling Jaya 47301, Selangor Darul Ehsan, Malaysia.

[e]Luminescent Materials and Devices Group, Department of Physics, National Institute of Technology, Warangal-506004, Telangana, INDIA

Corresponding author: ncgreddy@yogivemanauniversity.ac.in

# Equal contributors

Table of contents

	**Page No.**
**SI-1** Detailed calculations of green chemistry metrics of the model reactions for the synthesis of **3a** and **3x**	2-4
**SI-2:** Spectral data (^{1}H NMR, ^{13}C NMR and MS) for selected synthesized 2-arylquinoxalines (**3**).	4-7
**SI-3:** Copies of ^{1}H NMR, ^{13}C NMR and MS spectra of selected 2-arylquinoxalines (**3**)	8-25
SI-4: Mass spectral Evidence for the formation of intermediates I and III in the synthesis of 2-(4-bromophenyl)quinoxaline (**3d**) *via* the reaction between o-phenylenediamine (**1a**) and 4-bromophenacyl bromide (**2d**) under a nitrogen atmosphere at 55–60°C.	25-27

SI-1: Table S1: Detailed calculations of green chemistry metrics of the model reaction for the synthesis of **3a.**[1,2]

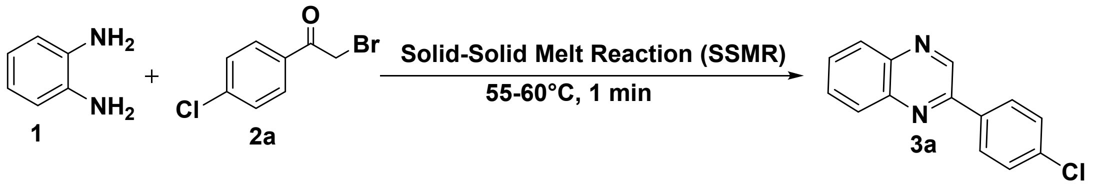

	**1**	**2a**	**3a**
**Mass**	0.216 g	0.464 g	0.475 g
**Moles**	0.002	0.002	0.00198
**GMW**	108	232	240

Reaction Solvent Mass: Nil

Mass of Purification Material: 1.1835g [Ethanol (1.5 mL]

Total input mass (^{m}inputs) = ^{m}**1** + ^{m}**2a** + ^{m}solvent (S) + ^{m}catalyst (C) + ^{m}workup materials (WPM) + ^{m}purification materials (PM) = 0.216 + 0.464+ 0+ 0 + 0 + 1.1835 = 1.8635 g

Mass of recovered materials: 0.95(1.2 mL of ethanol recovered from the 1.5 mL used in the purification of the product (3a))

Evaluation of Green Chemistry Metrics

**Atom Economy (AE) (%)**	= 100 (GMW of product / Sum of GMWs of reactants)<br>= 100 (240/340) = 70.6%
**Carbon efficiency (CE) (%)**	CE= [Amount of carbon in product/ Total carbon present in reactants] x 100 = [no. of moles of **3a** x no. of carbons in **3a** / (moles of **1** x carbons in **1** + moles of **2a** x carbons in **2a**] x 100 = [0.00198 x 14 / (0.002 x 6+ 0.002 x 8)] x 100 = [0.028/0.028)] x 100 = 100%
**E-factor (E)**	= Total input mass (^{m}inputs) - Mass of target product (^{m}**3a**) - Mass of recovered materials/Mass of target product (^{m}**3a**)<br>= 1.8635 – 0.475 – 0.95/0.475 = 0.92
**Process Mass Intensity (PMI)**	= (Total input mass (^{m}inputs) - Mass of recovered materials)/^{m}**3a**<br>= 1.8635 – 0.95/0.475 = 1.92<br>(Or)<br>= 1 + E = 1 + 0.923 = 1.92
**Curzon's Reaction Mass Efficiency (Curzon's RME) (%)**	= 100 **(**mass of **3a**/mass of **1** + mass of **2a)**<br>= 100 (0.475 /0.216+ 0.464) = 70%<br>(or)

	=100 (Yield of **3a** x Atom Economy x 1/Stoichiometric Factor)<br>= 100 (0.99 x 0.706 x 1) = 70% (∵ Stoichiometric Factor (SF) = 1)
**Generalized Reaction Mass Efficiency (gRME) (%)**	=100 [^{m}**3a**/(Total input mass (^{m}inputs) - Mass of recovered materials)]<br>= 100[0.475/1.8635 – 0.95] = 52%<br>(Or)<br>= 100[1/(1+E)] = 100[1/1 +0.923] = 52%

Table S2: Detailed calculations of green chemistry metrics of the model reaction for the synthesis of **3x.**[1,2]

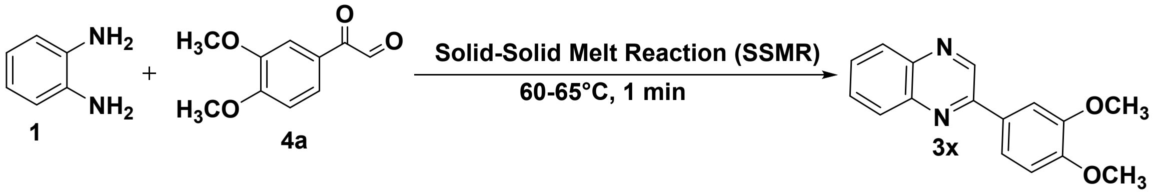

	1	2a	3a
**Mass**	0.216 g	0.388 g	0.527g
**Moles**	0.002	0.002	0.00198
**GMW**	108	194	266

Reaction Solvent Mass: Nil

Mass of Purification Material: 1.1835g [Ethanol (1.5 mL]

Total input mass (^{m}inputs) = ^{m}**1** + ^{m}**2a** + ^{m}solvent (S) + ^{m}catalyst (C) + ^{m}workup materials (WPM) + ^{m}purification materials (PM) = 0.216 + 0.388+ 0+ 0 + 0 + 1.1835 = 1.7875 g

Mass of recovered materials: 0.95 (1.2 mL of ethanol recovered from the 1.5 mL used in the purification of the product (3x))

Evaluation of Green Chemistry Metrics

**Atom Economy (AE) (%)**	= 100 (GMW of product / Sum of GMWs of reactants)<br>= 100 (266/302) = 88.1%
**Carbon efficiency (CE) (%)**	CE= [Amount of carbon in product/ Total carbon present in reactants] x 100 = [no. of moles of **3x** x no. of carbons in **3x** / (moles of **1** x carbons in **1** + moles of **4a** x carbons in **4a**] x 100 = [0.00198 x 16 / (0.002 x 6+ 0.002 x 10)] x 100 = [0.032/0.032)] x 100 = 100%
**E-factor (E)**	= Total input mass (^{m}inputs) - Mass of target product (^{m}**3x**) - Mass of recovered materials/Mass of target product (^{m}**3x**)<br>= 1.7875 – 0.527 – 0.95/0.527 = 0.59

**Process Mass Intensity (PMI)**	= (Total input mass (^{m}inputs) - Mass of recovered materials)/^{m}**3x**<br>= 1.7875 – 0.95/0.527 = 1.59<br>(Or)<br>= 1 + E = 1 + 0.59 = 1.59
**Curzon's Reaction Mass Efficiency (Curzon's RME) (%)**	**=** 100 **(**mass of **3x**/mass of **1** + mass of **4a)**<br>= 100 (0.527 /0.216+ 0.388) = 87.2%<br>(or)<br>=100 (Yield of **3x** x Atom Economy x 1/Stoichiometric Factor)<br>= 100 (0.99 x 0.881 x 1) = 87.2% (∵ Stoichiometric Factor (SF) = 1)
**Generalized Reaction Mass Efficiency (gRME) (%)**	=100 [^{m}**3x**/(Total input mass (^{m}inputs) - Mass of recovered materials)]<br>= 100[0.527/1.7875 – 0.95] = 62.9%<br>(Or)<br>= 100[1/(1+E)] = 100[1/1 +0.59] = 62.9%

References:

1. J. Andraos, A. Hent, Simplified application of material efficiency green metrics to synthesis plans: pedagogical case studies selected from organic syntheses, *J. Chem. Educ.,* 2015, *92*, 1820–1830.
2. A. P. Dicks, A. Hent, Green Chemistry Metrics: A Guide to Determining and Evaluating Process Greenness, First ed.*,* Springer Cham, Switzerland, 2015.

SI-2. Spectral data for selected synthesized 2-arylquinoxalines (3).

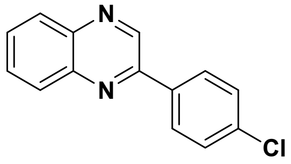<br>**2-(4-chlorophenyl)quinoxaline (3a)**	Yellow solid, ^{1}H NMR (400MHz, $CDCl_3$): δ (ppm) 9.22 (s, 1H, ArH), 8.08 (d, J = 8.4 Hz, 2H, ArH), 8.04(dd, ^{1}J = 8.4 Hz, ^{2}J = 1.2 Hz, 2H, ArH), 7.74-7.66 (m, 2H, ArH), 7.46 (d, J=8.4 Hz, 2H, ArH); ^{13}C-NMR (100 MHz, $CDCl_3$): δ 150.66, 142.67, 142.28, 141.41, 136.69, 135.11, 130.62, 129.80, 129.61, 129.46 (2C), 129.04, 128.81 (2C); MS(ESI): $[M+H]^{+}$ 241.3
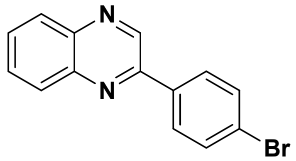	Orange yellow solid, ^{1}H NMR (400MHz, d_6-DMSO): δ (ppm) 9.61 (s, 1H, ArH), 8.31(d, J=8.8 Hz, 2H, ArH), 7.84 (td, ^{1}J=8.0 Hz,

**2-(4-bromophenyl)quinoxaline (3d)**	^{2}J=1.6 Hz, 2H, ArH), 7.93-7.85 (m, 2H, ArH), 7.82(d, J=8.4 Hz, 2H, ArH), MS(ESI): $[M+H]^+$ 285.0, $[M+H+2]^+$ 287.3.
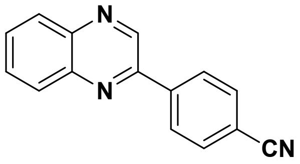 **4-(quinoxalin-2-yl)benzonitrile (3g)**	Yellow solid, ^{1}H NMR (400MHz, $CDCl_3$): δ (ppm) 9.28 (s, 1H, ArH), 8.27(d, J = 8.4 Hz, 2H, ArH), 8.04(td, ^{1}J = 8.2 Hz, ^{2}J = 1.8 Hz, 2H, ArH), 7.79 (d, J=8.4, 2H, arom H), 7.77-7.72 (m, 2H, ArH); ^{13}C-NMR (100 MHz, $CDCl_3$): δ 149.53, 142.66, 142.17, 141.96, 140.77, 132.83(2C), 130.80, 130.53,129.76, 129.19, 127.99 (2C), 118.42, 113.65. MS (ESI): $[M+H]^+$ 232.3.
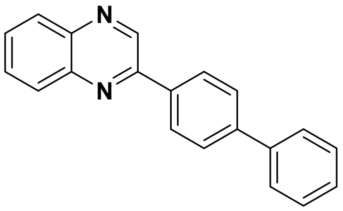 **2-([1,1'-biphenyl]-4-yl)quinoxaline (3h)**	Orange yellow solid, ^{1}H NMR (400MHz, d_6-DMSO): δ (ppm) 9.66 (s, 1H, ArH), 8.49 (d, J = 8.4 Hz, 2H, ArH), 8.16-8.13 (m, , 2H, ArH), 7.94-7.84 (m, 4H, ArH), 7.80 (d, J = 7.6 Hz, 2H, ArH), 7.53 (t, J = 7.6 Hz, 2H, ArH),), 7.43 (t, J = 7.4 Hz, 1H, ArH); MS(ESI): $[M+H]^+$ 283.4
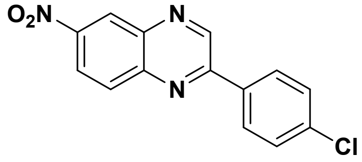 **2-(4-chlorophenyl)-6-nitroquinoxaline (3i)**	Brown solid, ^{1}H NMR (400MHz, d_6-DMSO): δ (ppm) 9.82 (s, 1H, ArH), 8.94 (d, J = 2.4 Hz, 1H, ArH), 8.59 (dd, ^{1}J = 9.2 Hz, ^{2}J = 2.8 Hz, 1H, ArH), 8.4-8.36 (m, 3H, ArH), 7.87 (d, J = 8.8 Hz, 2H, ArH).
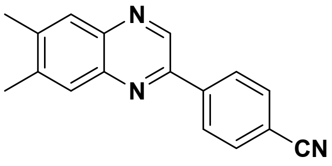 **4-(6,7-dimethylquinoxalin-2-yl)benzonitrile (3u)**	Orange yellow solid,^{1}H NMR (400MHz, d_6-DMSO): δ (ppm) 9.54 (s, 1H, ArH), 8.49 (d, J = 8.4 Hz, 2H, ArH), 8.06 (d, J = 8.4 Hz, 2H, ArH), 7.94 (s, 1H, ArH), 7.91 (s, 1H, ArH), 2.50 (s, 6H, $-2CH_3$); MS(ESI): $[M+H]^+$ 260.08

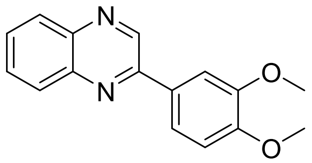<br>**2-(3,4-dimethoxyphenyl)quinoxaline (3x)**	Pale yellow solid, ^{1}H NMR (400MHz, d_6-DMSO): δ (ppm) 9.60 (s, 1H, ArH), 8.01 (td, ^{1}J = 8.8 Hz, ^{2}J = 1.6 Hz, 2H, ArH), 7.97(dd, ^{1}J = 8.4 Hz, ^{2}J = 2.4 Hz, 1H, ArH), 7.93 (d, J = 2.4 Hz, 1H, ArH), 7.88-7.78 (m, 2H, ArH), 7.17 (d, J = 8.4 Hz, 1H, ArH), 3.92 (s, 3H, -OCH_3), 3.87 (s, 3H, -OCH_3); MS(ESI): $[M+H]^+$ 267.3
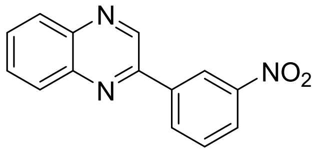<br>**2-(3-nitrophenyl)quinoxaline (3y)**	Off-white solid, ^{1}H NMR (400MHz, d_6-DMSO): δ (ppm) 9.73 (s, 1H, ArH), 9.11 (d, J = 1.6 Hz, 1H, ArH), 8.80(d, J = 7.6 Hz, 1H, ArH), 8.41 (dd, ^{1}J = 8.0 Hz, ^{2}J = 2.0 Hz,, 1H, ArH), 8.23 (dd, ^{1}J = 7.6 Hz, ^{2}J = 2.4 Hz,, 1H, ArH), 8.17 (dd, ^{1}J = 7.2 Hz, ^{2}J = 2.4 Hz,, 1H, ArH), 7.96-7.89 (m, 3H, ArH); MS(ESI): $[M+H]^+$ 252.3
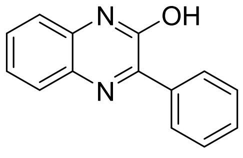<br>**3-phenylquinoxalin-2-ol (3ab)**	White solid, ^{1}H NMR (400MHz, d_6-DMSO): δ (ppm) 12.57 (s, 1H, -OH), 8.32-8.28 (m, 2H, ArH), 7.84 (d, J = 8.0 Hz, 1H, ArH), 7.56-7.49 (m, 4H, ArH), 7.35-7.30 (m, 2H, ArH); MS(ESI): $[M+H]^+$ 223.3
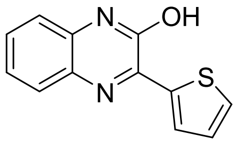<br>**3-(thiophen-2-yl)quinoxalin-2-ol (3ac)**	Green solid, ^{1}H NMR (400MHz, d_6-DMSO): δ (ppm) 12.70 (s, 1H, -OH), 8.41 (dd, ^{1}J = 4.0 Hz, ^{2}J = 1.6 Hz, 1H, ArH), 7.84 (dd, ^{1}J = 5.0 Hz, ^{2}J = 1.0 Hz, 1H, ArH), 7.78 (d, J = 8.0 Hz, 1H, ArH), 7.52 (td, ^{1}J = 7.8 Hz, ^{2}J = 1.2 Hz, 1H, ArH), 7.36-7.31 (m, 2H, ArH), 7.24 (dd, ^{1}J = 5.0 Hz, ^{2}J = 4.2 Hz, 1H, ArH); MS(ESI): $[M+H]^+$ 229.2

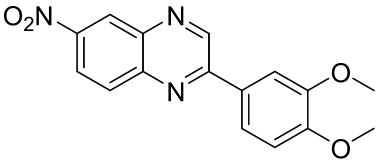 **2-(3,4-dimethoxyphenyl)-6-nitroquinoxaline (3ad)**	Yellow solid, ^{1}H NMR (400MHz, d_6-DMSO): δ (ppm) 9.81 (d, J = 5.2 Hz, 1H, ArH), 8.88 (d, J = 2.4 Hz, 1H, ArH), 8.56-8.47(m, 1H, ArH), 8.33-8.29 (m, 1H, ArH), 8.09-8.03 (m, 1H, ArH), 7.98 (d, J = 2.0 Hz, 1H, ArH), 7.20 (dd, ^1J = 8.6 Hz, ^2J = 2.6 Hz, 1H, ArH), 3.93 (s, 3H, -OCH_3), 3.89 (s, 3H, -OCH_3); MS(ESI): $[M+H]^+$ 312.4
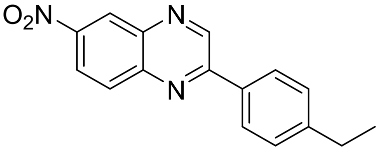 **2-(4-ethylphenyl)-6-nitroquinoxaline (3ae)**	Off-white solid, ^{1}H NMR (400MHz, d_6-DMSO): δ (ppm) 9.79(s, 1H, ArH), 8.91 (d, J = 2.4 Hz, 1H, ArH), 8.57 (dd, ^1J = 8.8 Hz, ^2J = 2.6 Hz, 1H, ArH), 8.37-8.32 (m, 3H, ArH), 7.49 (d, J = 8.4 Hz, 2H, ArH), 2.73 (q, J = 7.6 Hz, 2H, -CH_2), 1.25 (t, J = 7.6 Hz, 3H, -CH_3); MS(ESI): $[M+H]^+$ 280.4
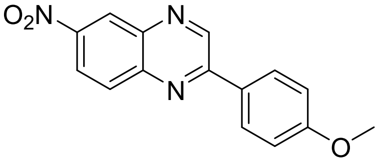 **3-(4-methoxyphenyl)-7-nitro-1,2-dihydroquinoxaline (3ag)**	Yellow solid, ^{1}H NMR (400MHz, d_6-DMSO): δ (ppm) 9.77 (d, J = 5.2 Hz, 1H, ArH), 8.88 (dd, ^1J = 6.8 Hz, ^1J = 2.4 Hz, 1H, ArH), 8.56-8.47 (m,1H, ArH), 8.44-8.39 (m, 2H, ArH), 8.33-8.28 (m, 1H, ArH), 7.19 (d, J = 8.8 Hz, 2H, ArH), 3.89 (s, 3H, -OCH_3); MS(ESI): $[M+H]^+$ 282.3
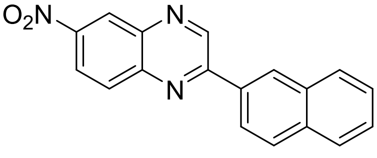 **2-(naphthalen-2-yl)-6-nitroquinoxaline (3ai)**	Yellow solid, ^{1}H NMR (400MHz, d_6-DMSO): δ (ppm) 9.50 (s, 1H, ArH), 9.02 (d, J = 2.4 Hz, 1H, ArH), 8.64 (dd, ^1J = 9.0 Hz, ^2J = 2.4 Hz, 1H, ArH), 8.42 (d, J = 9.2 Hz, 1H, ArH), 8.30 (d, J = 8.0 Hz, 1H, ArH), 8.20 (d, J = 8.4 Hz, 1H, ArH), 8.12 (dd, ^1J = 7.6 Hz, ^2J = 1.6 Hz, 1H, ArH), 7.99 (dd, ^1J = 7.0 Hz, ^2J = 1.0 Hz, 1H, ArH), 7.77-7.73 (m, 1H, ArH), 7.67-7.59 (m, 2H, ArH); MS(ESI): $[M+H]^+$ 302.3

SI-3: Copies of ¹H NMR, ¹³C NMR and MS spectra of selected 2-arylquinoxalines (3)

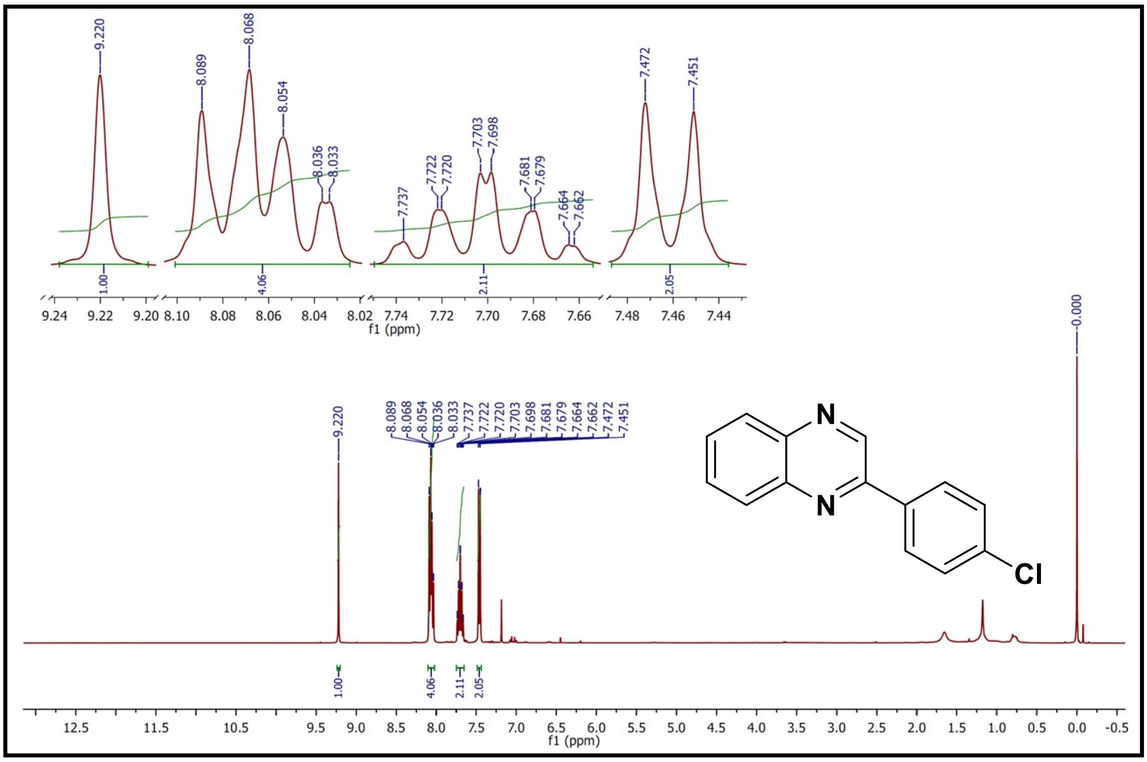

Figure S1. ^{1}H NMR spectrum of 2-(4-chlorophenyl)quinoxaline (**3a**).

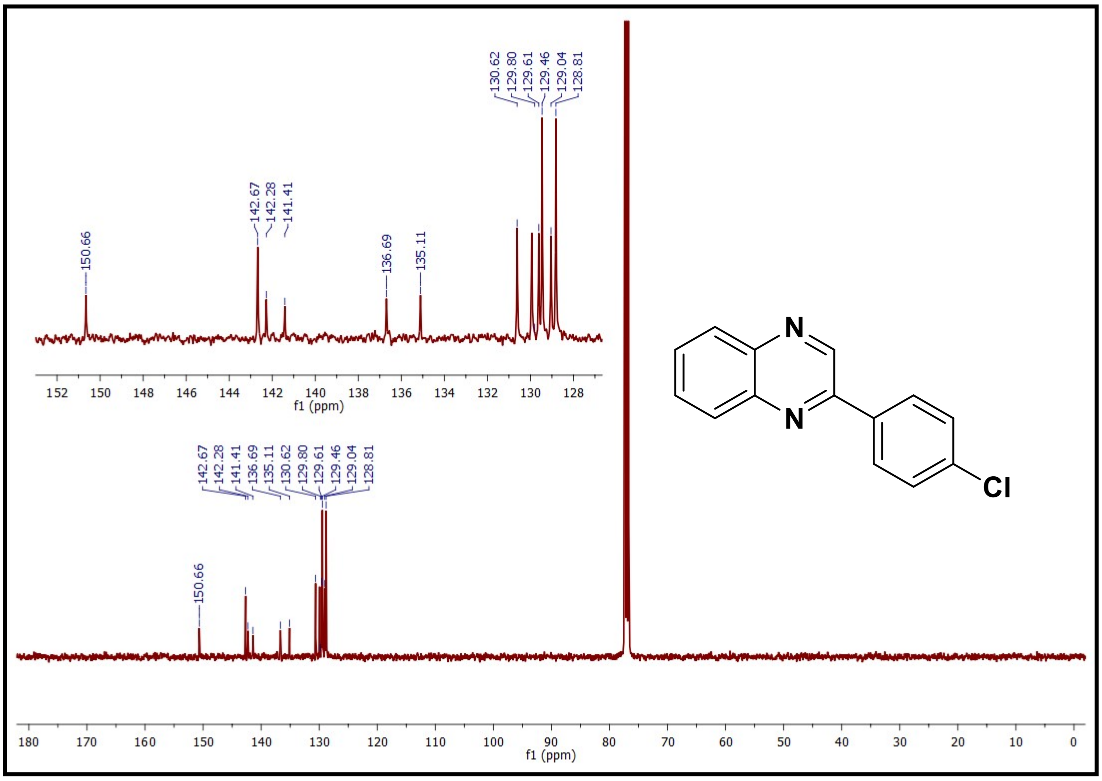

Figure S2. ^{13}C-NMR spectrum of 2-(4-chlorophenyl)quinoxaline (**3a**).

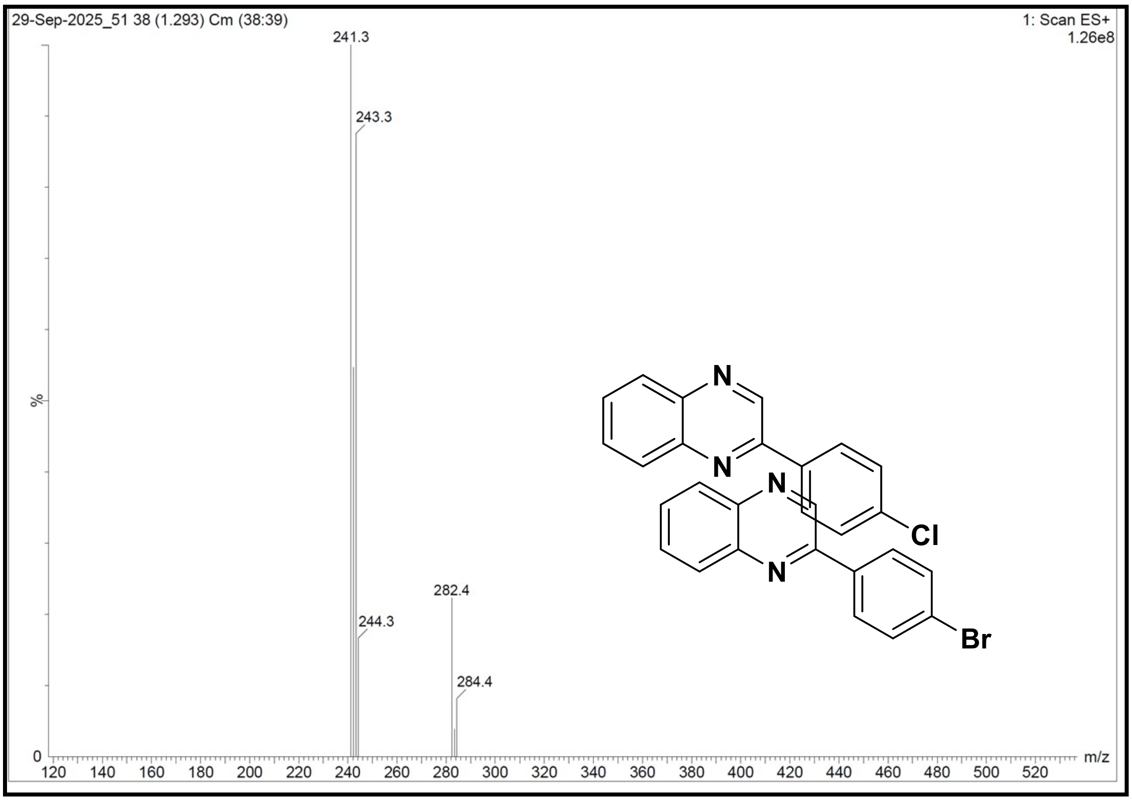

Figure S3. Mass spectrum of 2-(4-chlorophenyl)quinoxaline (**3a**).

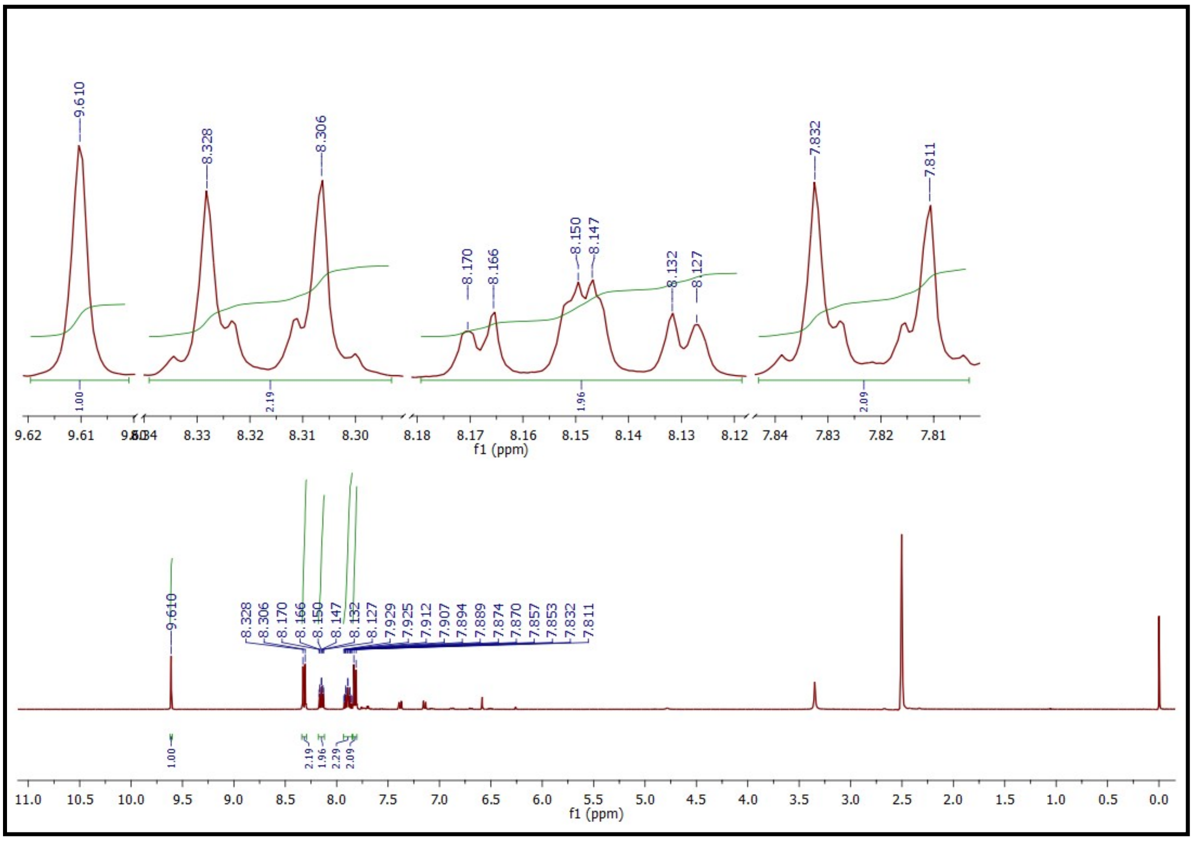

Figure S4. ^{1}H-NMR spectrum of 2-(4-bromophenyl)quinoxaline (**3d**).

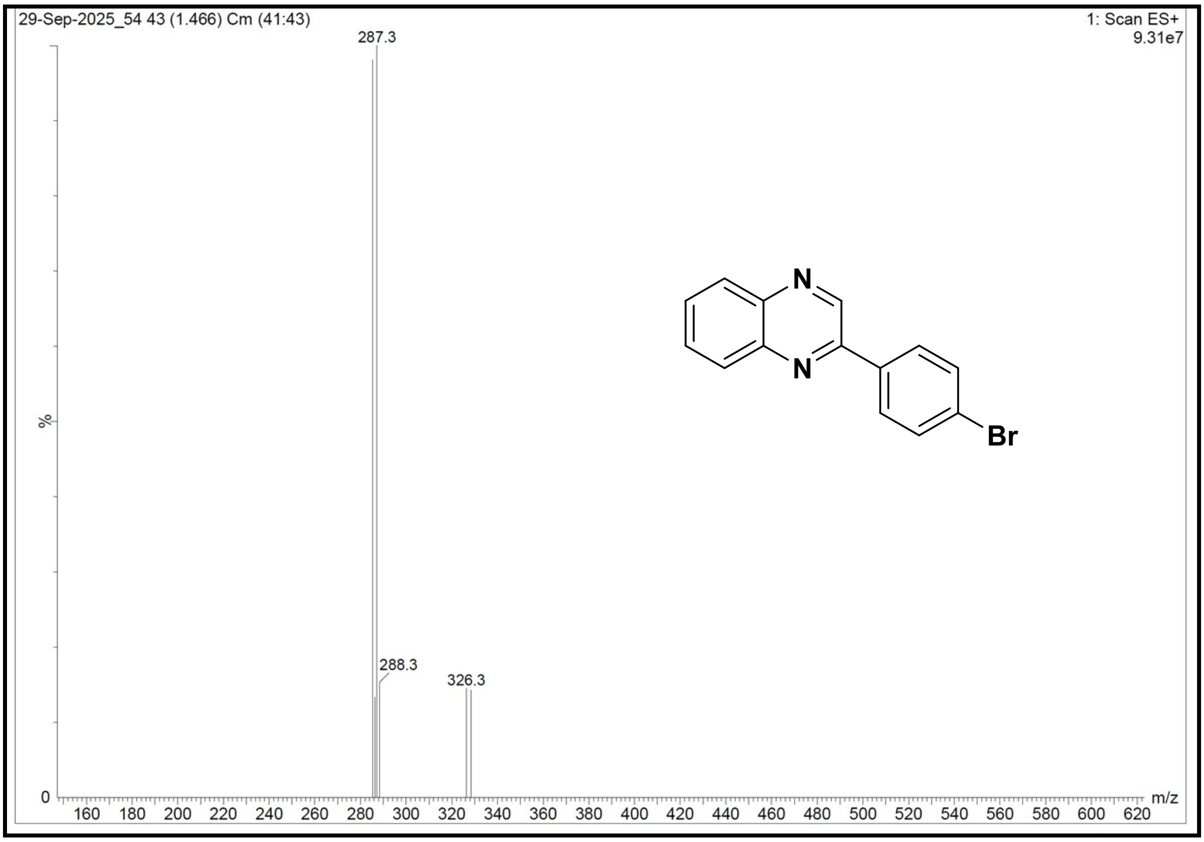

Figure S5. Mass spectrum of 2-(4-bromophenyl)quinoxaline (**3d**)

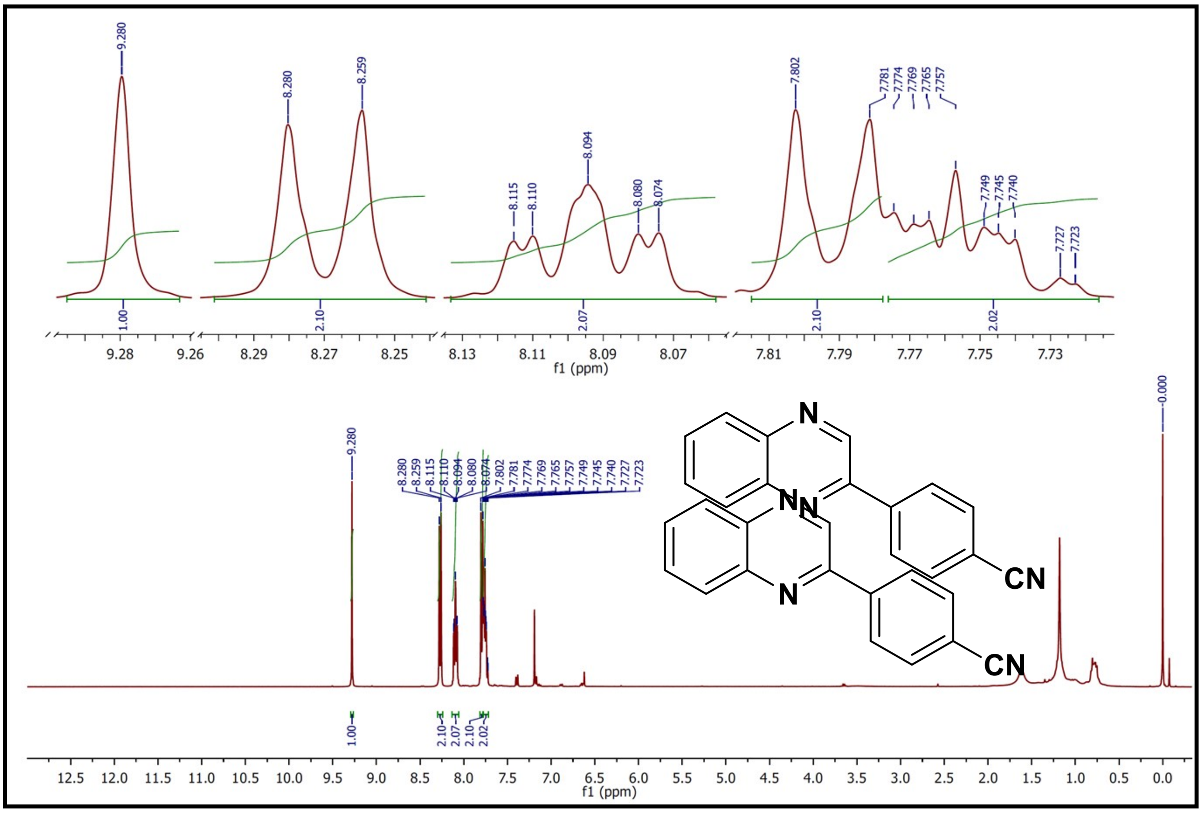

Figure S6. ^{1}H-NMR spectrum of 4-(quinoxalin-2-yl)benzonitrile (**3g**).

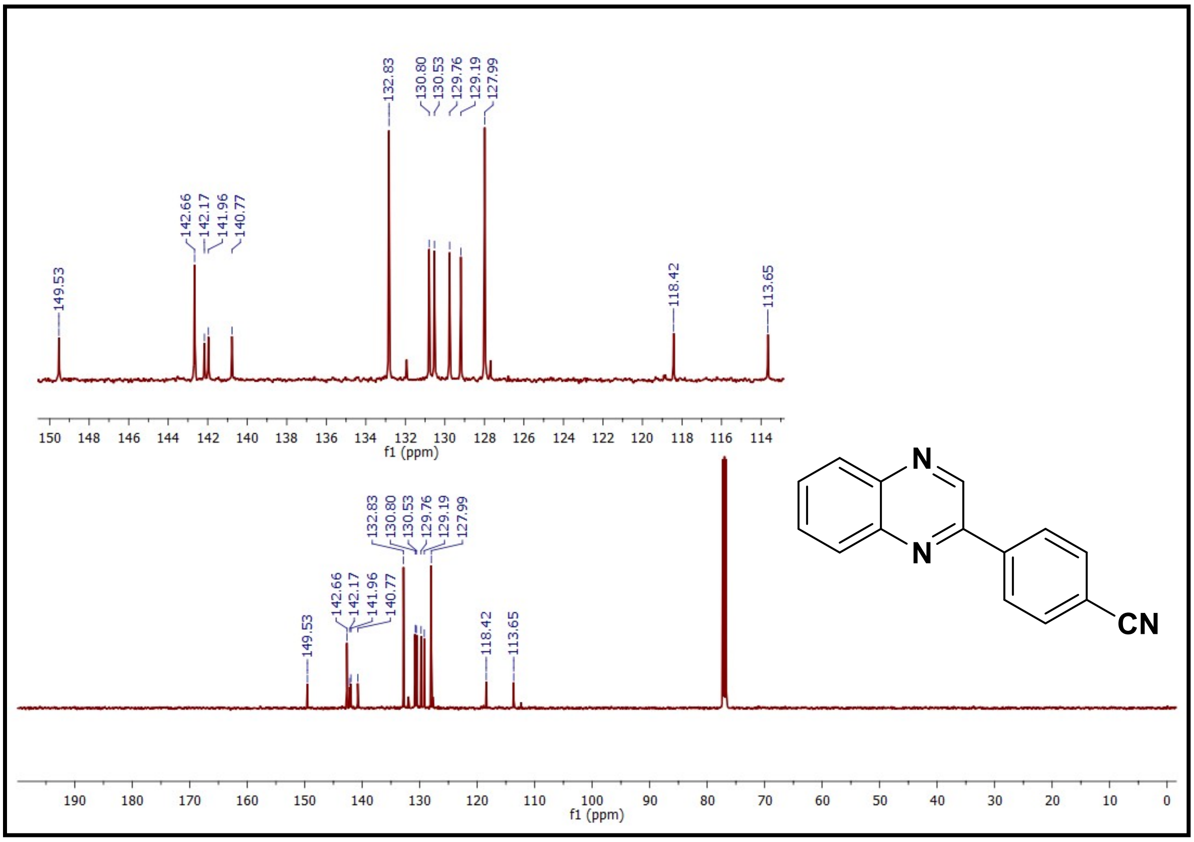

Figure S7. ^{13}C-NMR spectrum of 4-(quinoxalin-2-yl)benzonitrile (**3g**).

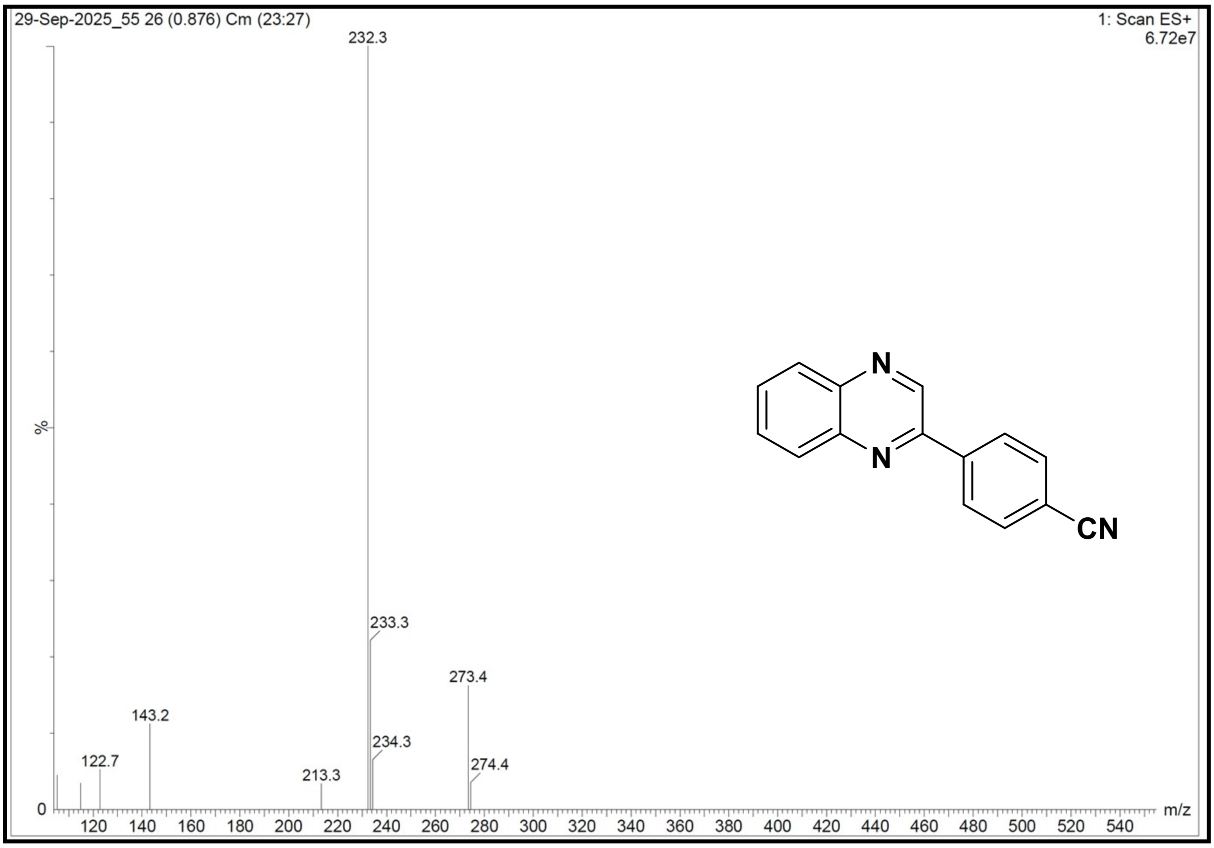

Figure S8. Mass spectrum of 4-(quinoxalin-2-yl)benzonitrile (**3g**).

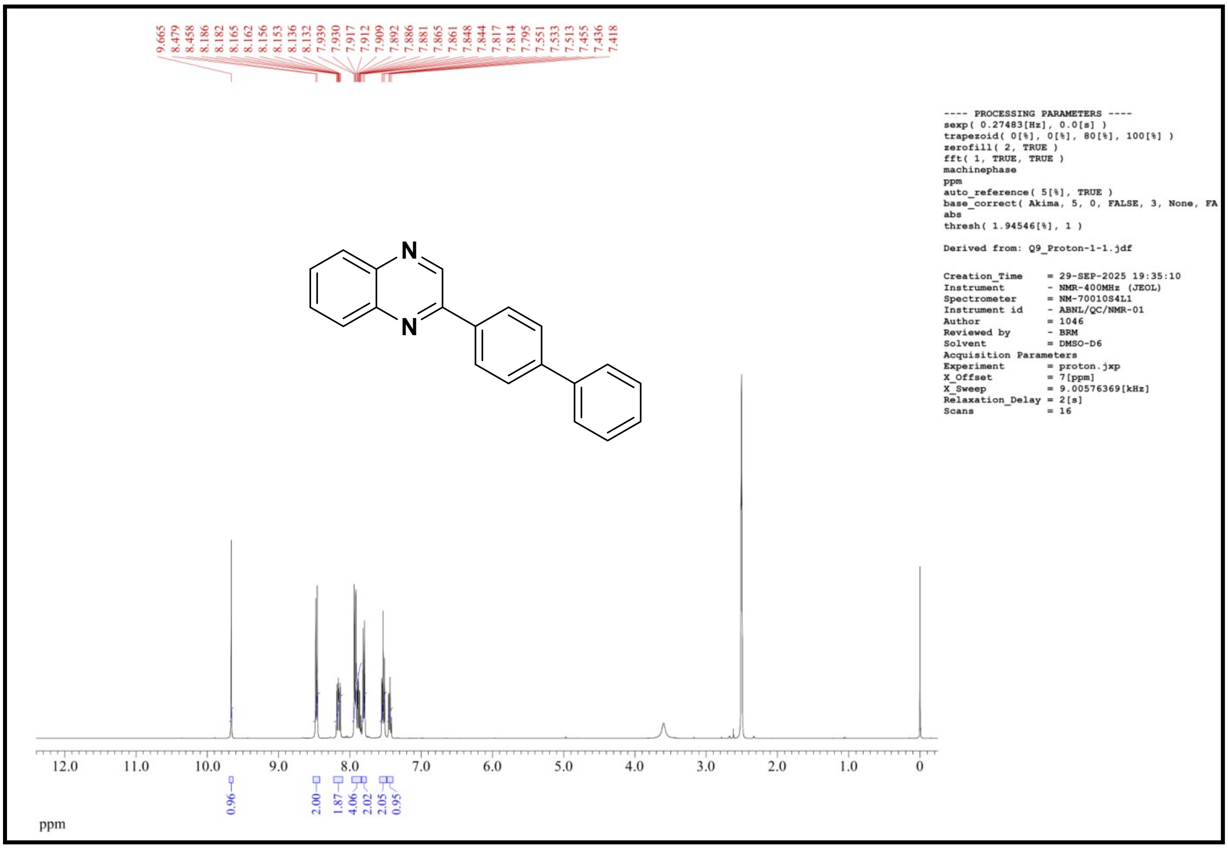

Figure S9. ^{1}H-NMR Spectrum of 2-([1,1'-biphenyl]-4-yl)quinoxaline (**3h**).

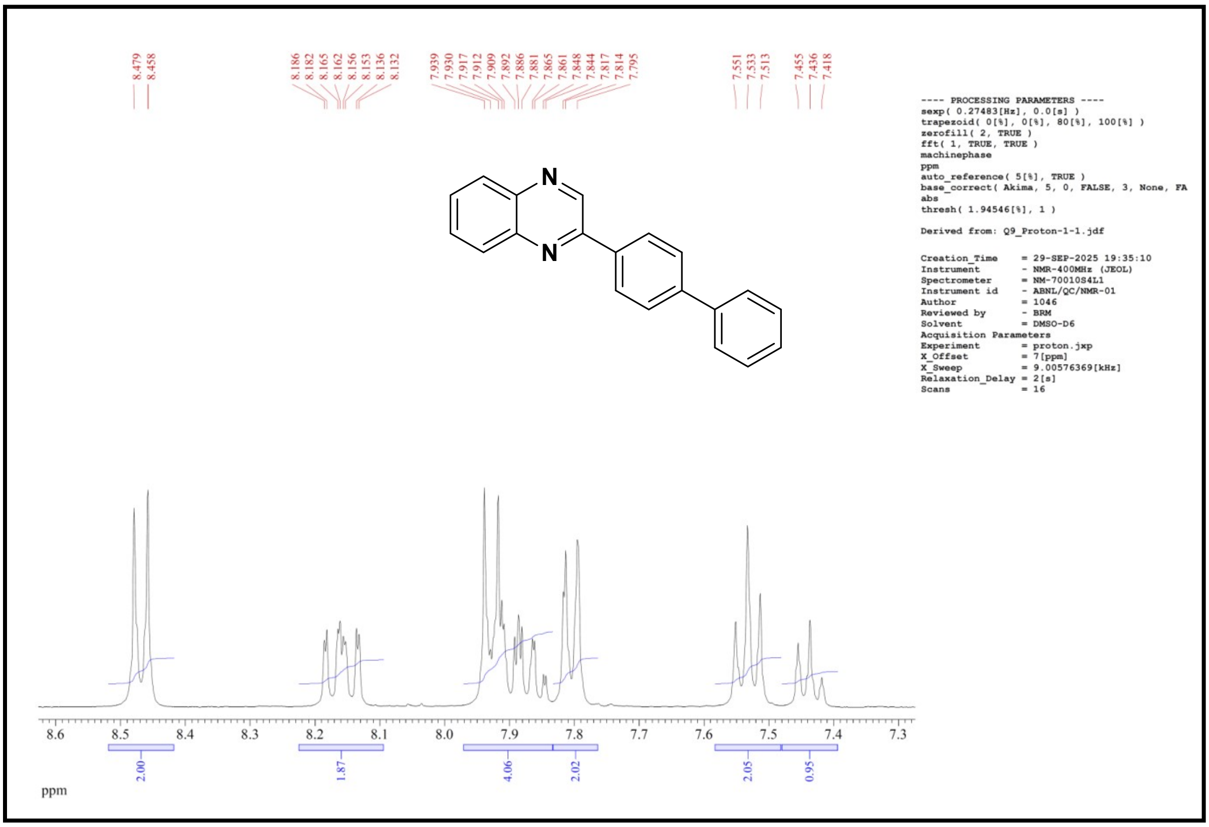

Figure S10. Expanded ^{1}H-NMR Spectrum of 2-([1,1'-biphenyl]-4-yl)quinoxaline (**3h**).

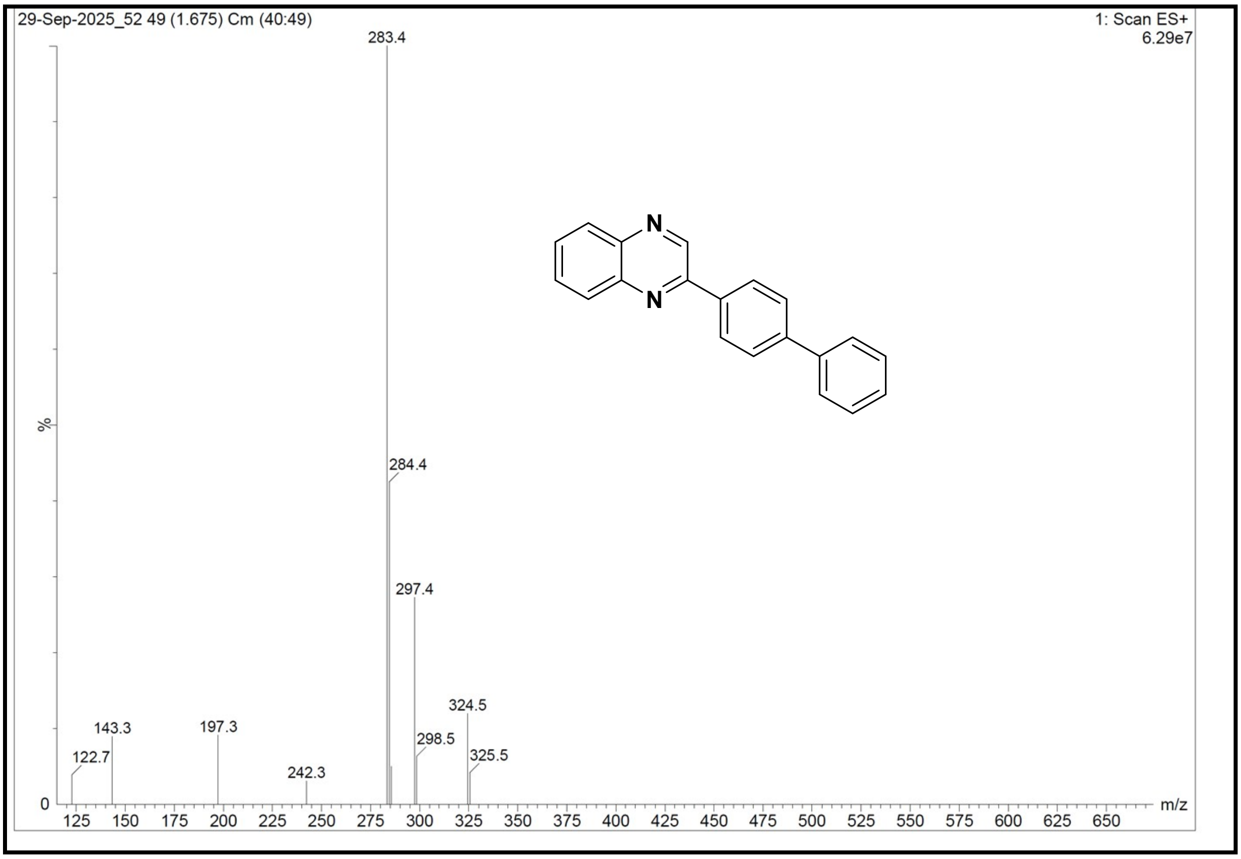

Figure S11. Mass spectrum of 2-([1,1'-biphenyl]-4-yl)quinoxaline (**3h**).

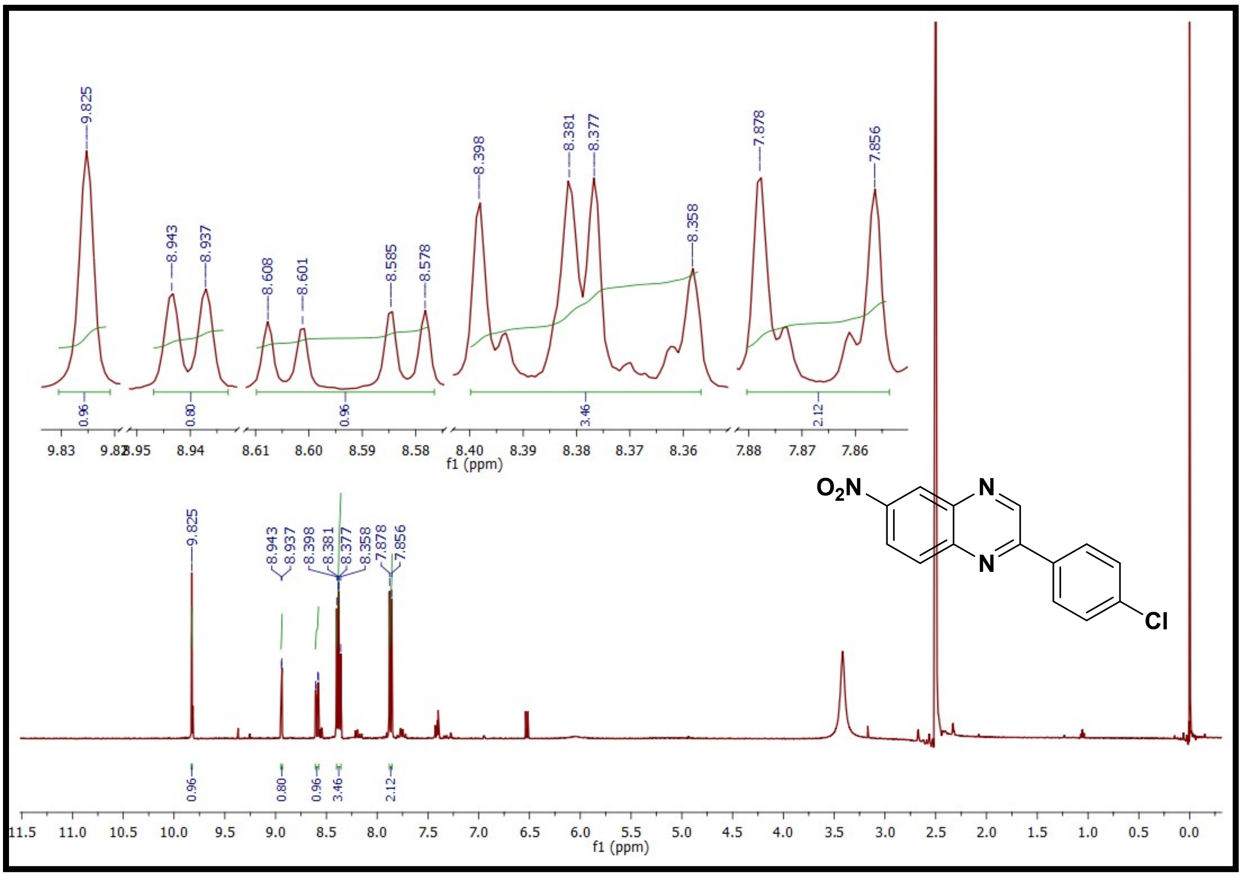

Figure S12. ^{1}H-NMR spectrum of 2-(4-chlorophenyl)-6-nitroquinoxaline (**3i**)

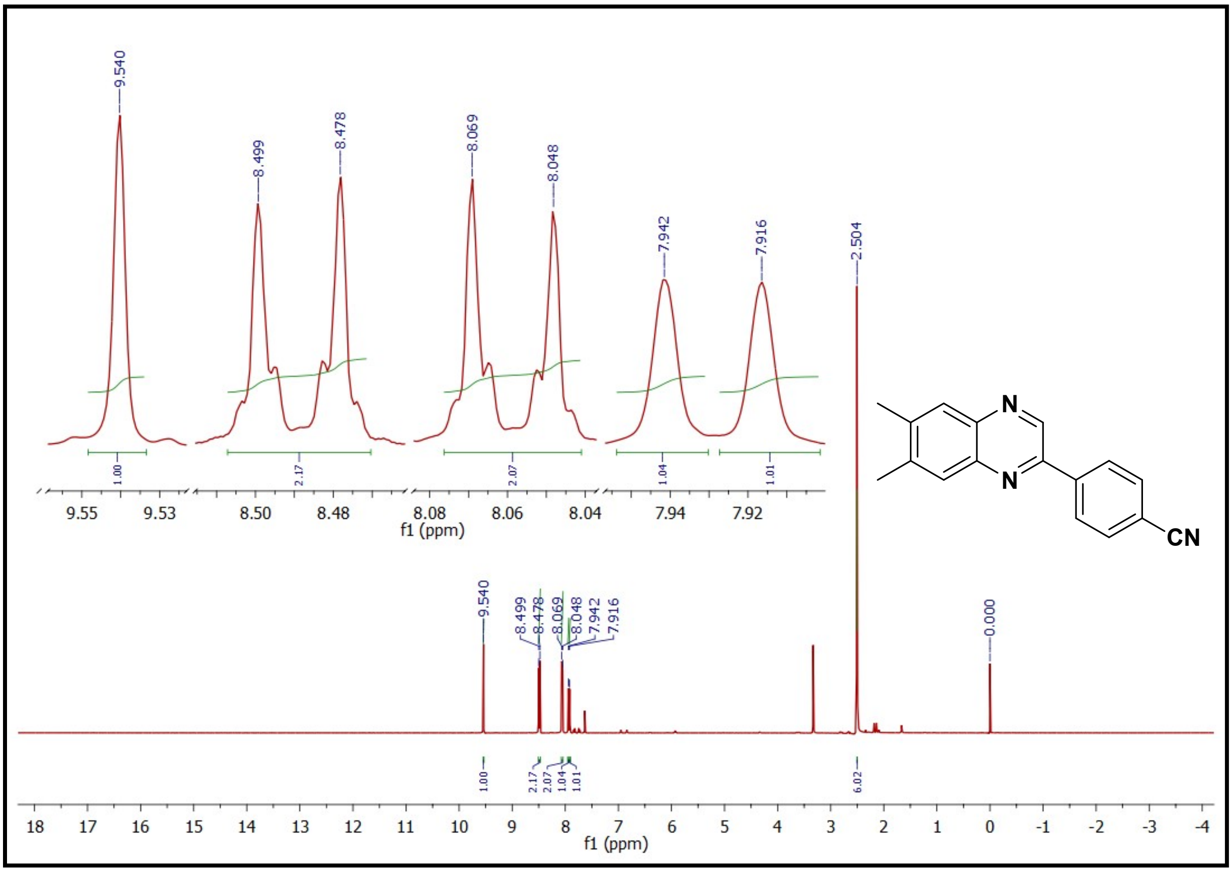

. **Figure S13.** ^{1}H-NMR spectrum of 4-(6,7-dimethylquinoxalin-2-yl)benzonitrile (**3u**).

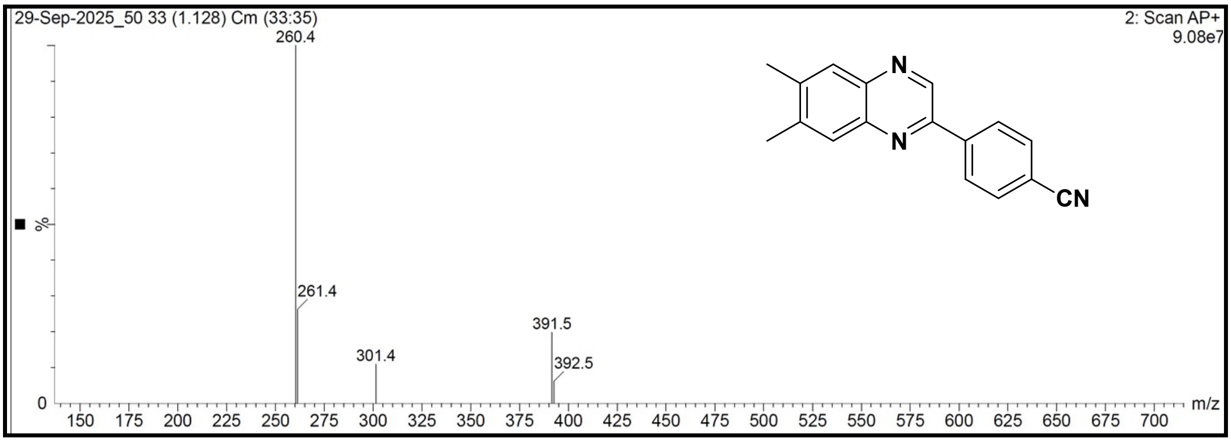

Figure S14. Mass spectrum of 4-(6,7-dimethylquinoxalin-2-yl)benzonitrile (**3u**).

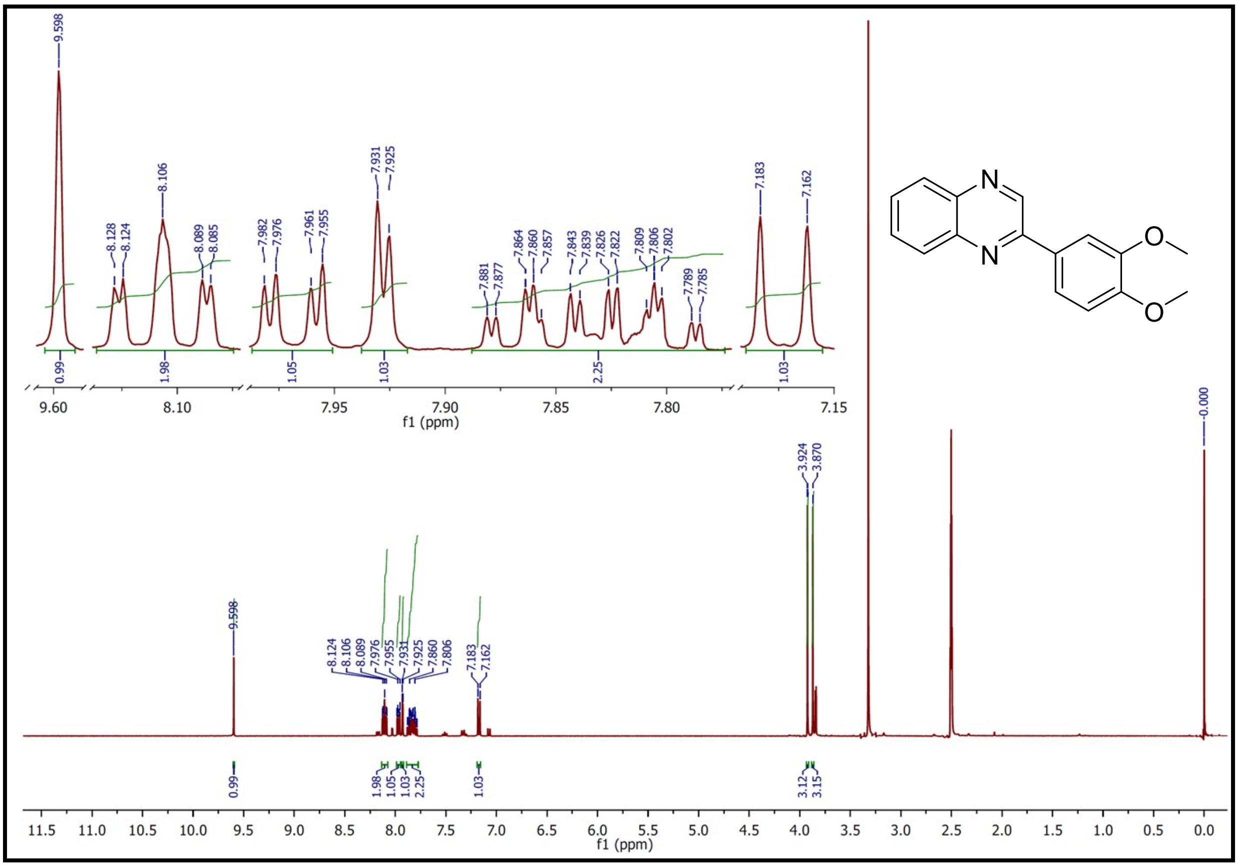

Figure S15. ^{1}H-NMR spectrum of 2-(3,4-dimethoxyphenyl)quinoxaline (**3x**).

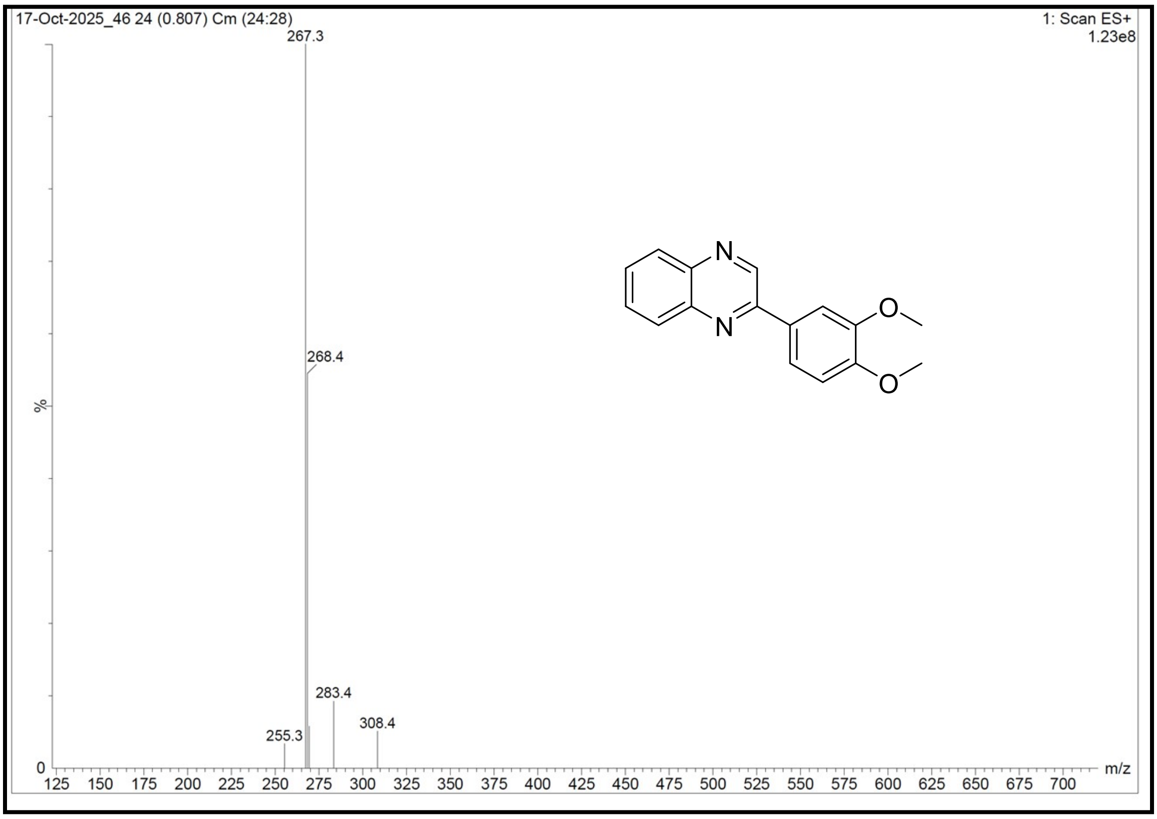

Figure S16. Mass spectrum of 2-(3,4-dimethoxyphenyl)quinoxaline (**3x**).

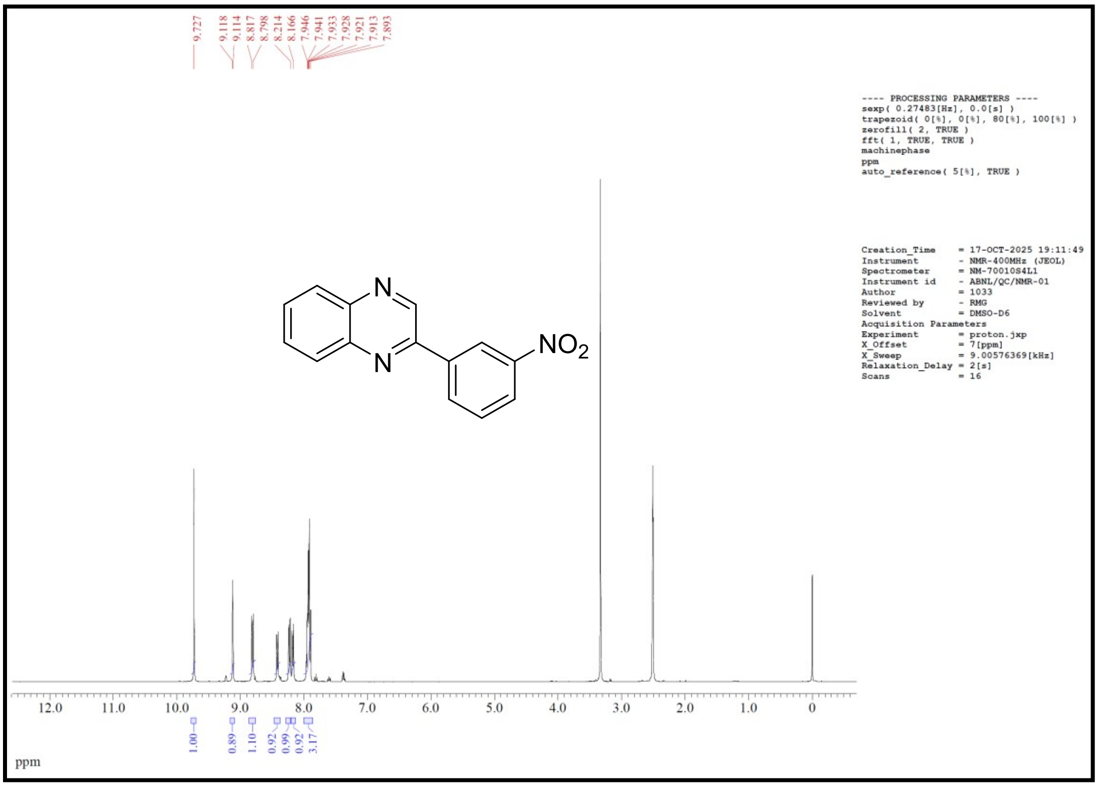

Figure S17. ^{1}H-NMR spectrum of 2-(3-nitrophenyl)quinoxaline **(3y)**.

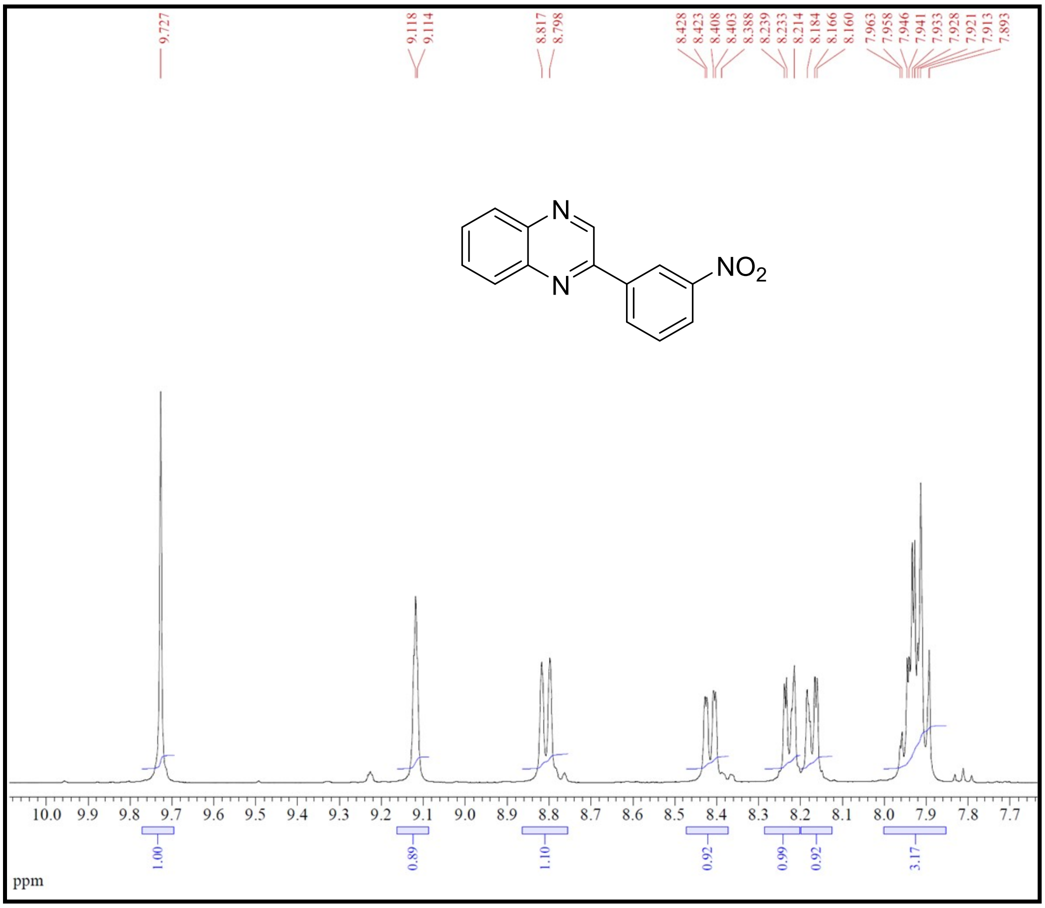

Figure S18. Expanded ^{1}H-NMR spectrum of 2-(3-nitrophenyl)quinoxaline **(3y)**.

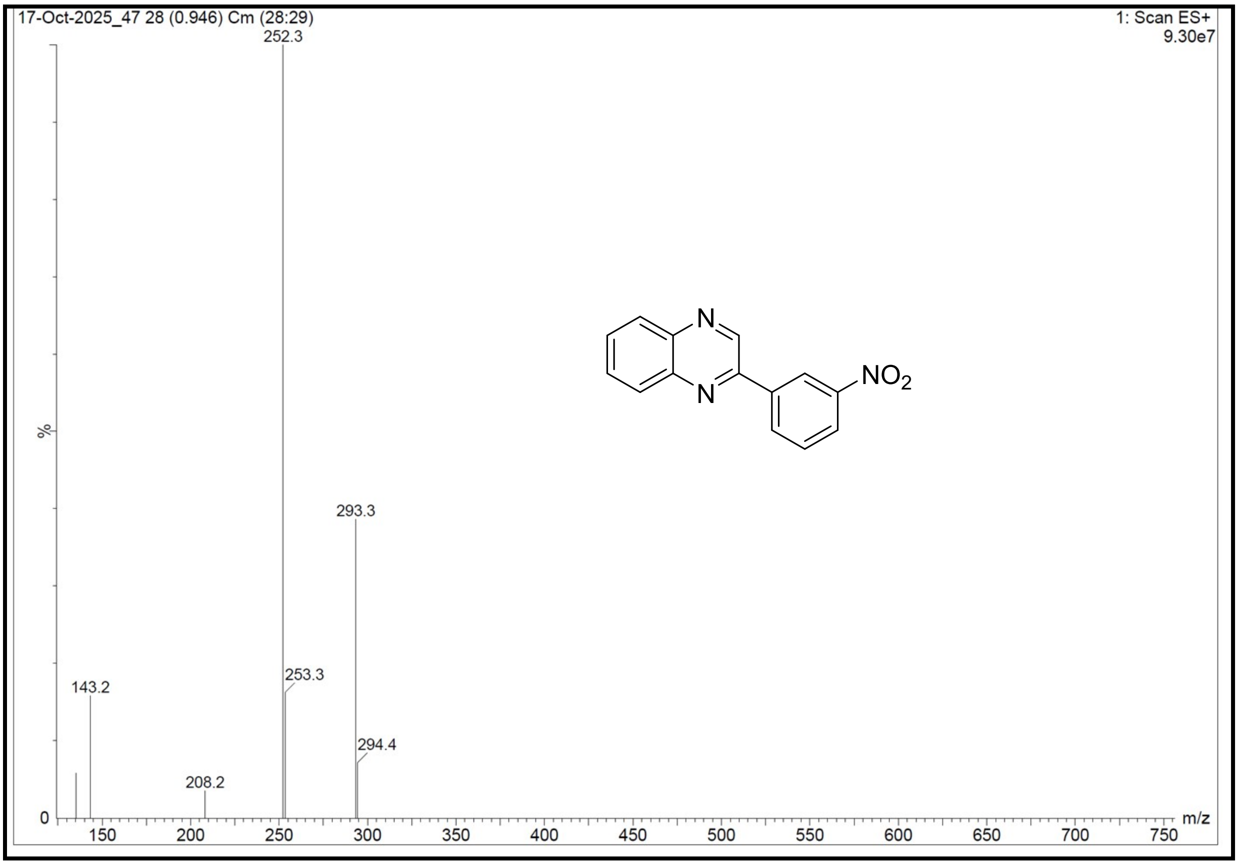

Figure S19. Mass spectrum of 2-(3-nitrophenyl)quinoxaline **(3y)**.

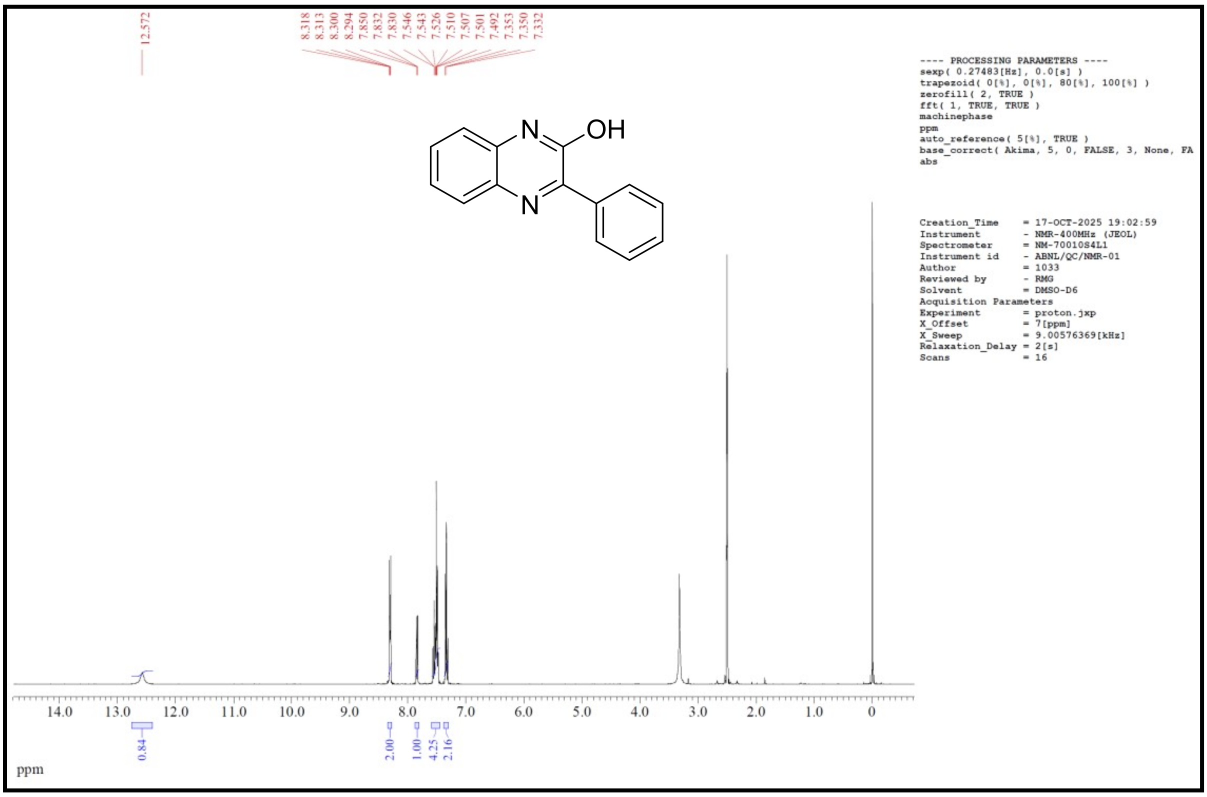

Figure S20. ^{1}H-NMR spectrum of 3-phenylquinoxalin-2-ol (**3ab**).

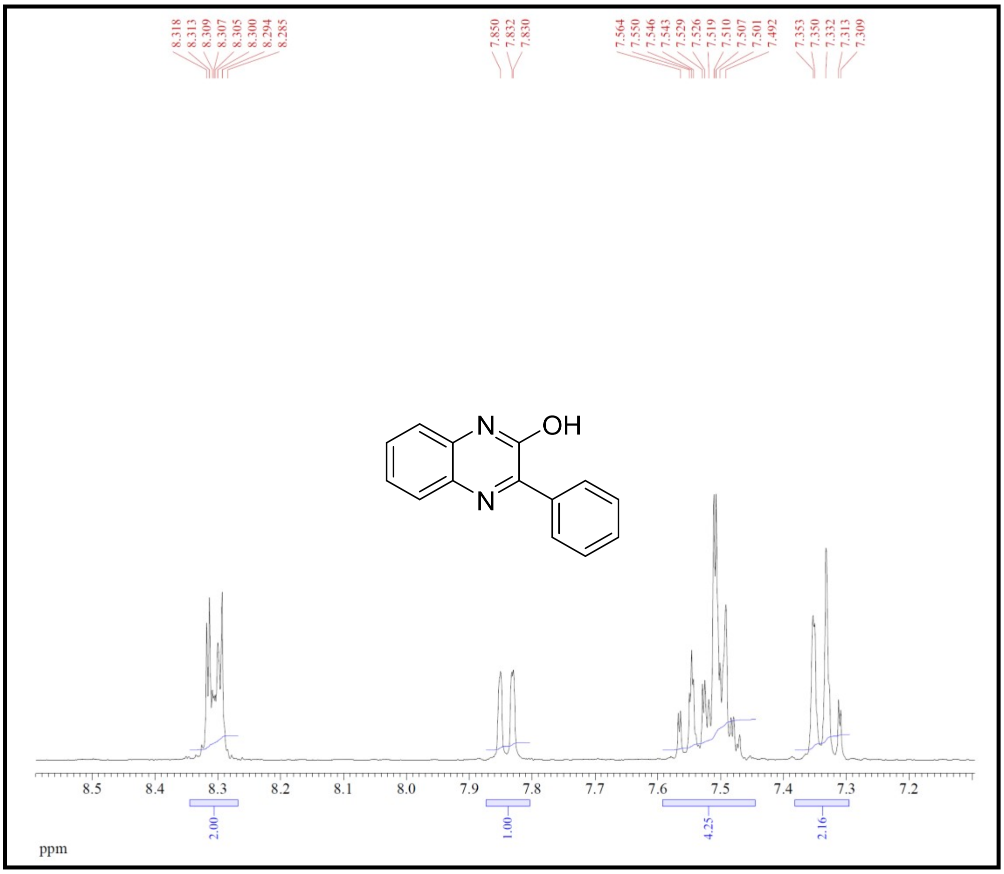

Figure S21. Expanded ^{1}H-NMR spectrum of 3-phenylquinoxalin-2-ol (**3ab**).

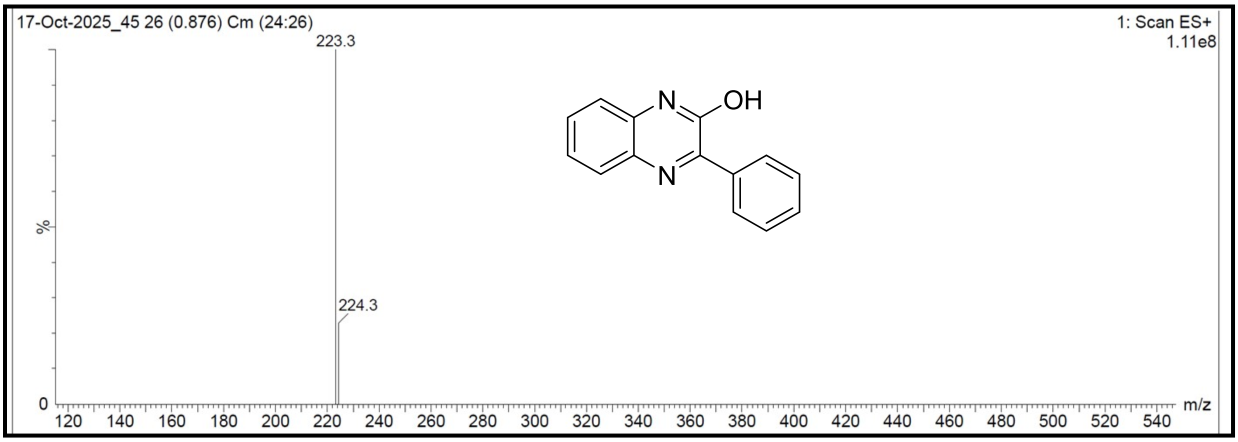

Figure S22. Mass spectrum of 3-phenylquinoxalin-2-ol (**3ab**).

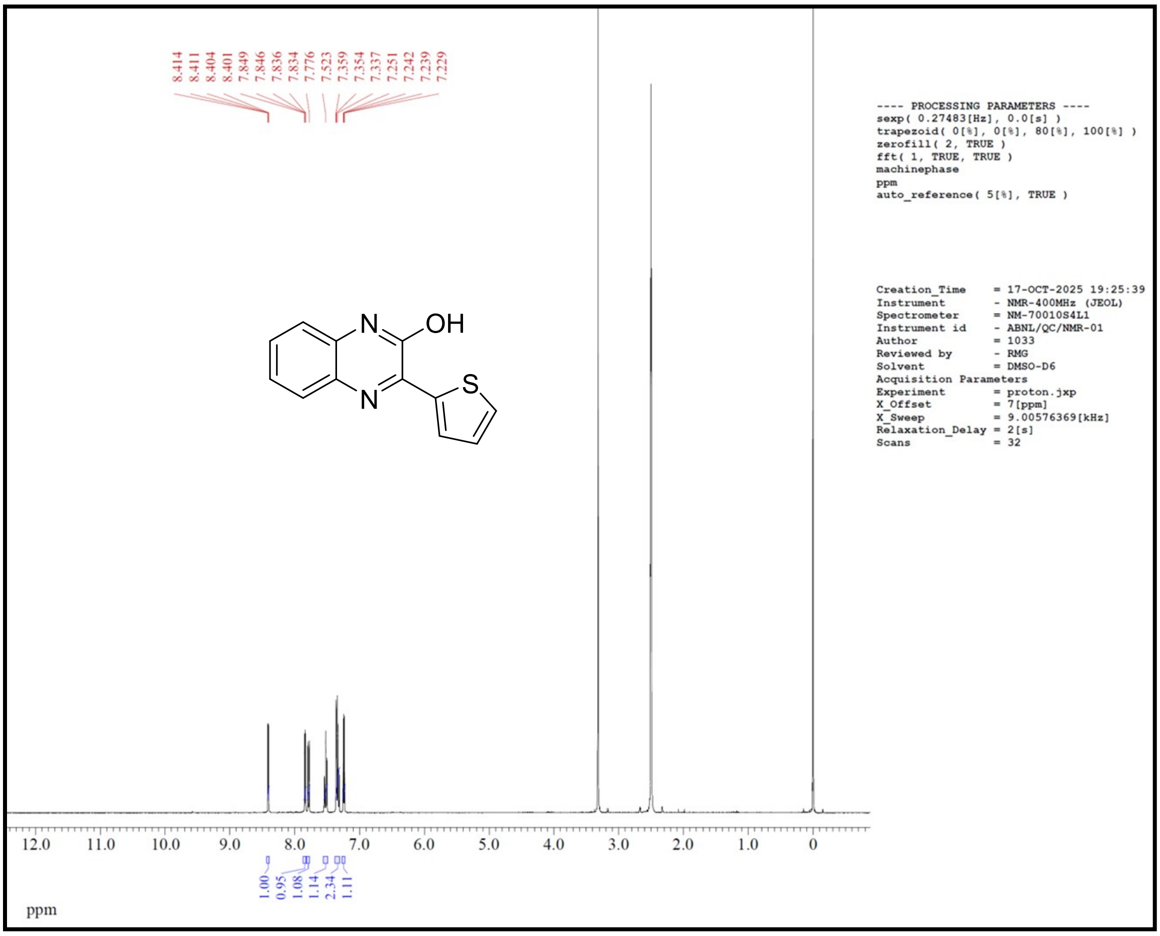

Figure S23. ^{1}H-NMR spectrum of 3-(thiophen-2-yl)quinoxalin-2-ol (**3ac**).

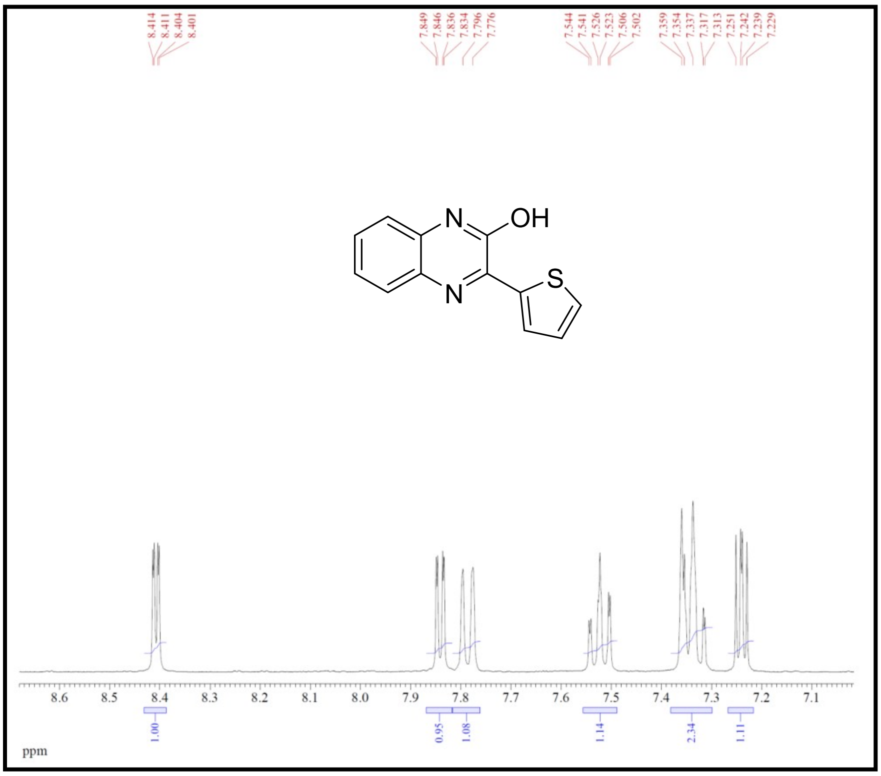

Figure S24. Expanded ^{1}H-NMR spectrum of 3-(thiophen-2-yl)quinoxalin-2-ol (**3ac**).

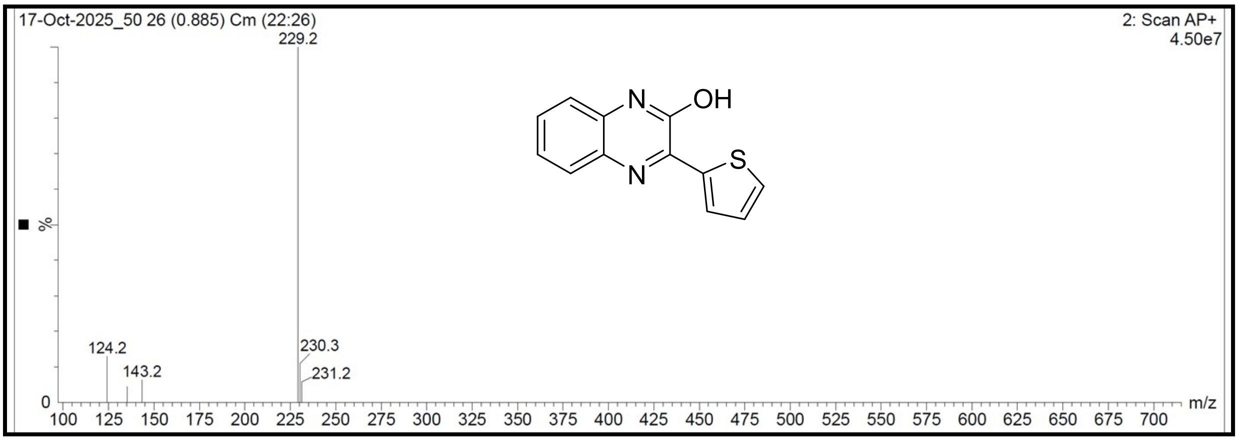

Figure S25. Mass spectrum of 3-(thiophen-2-yl)quinoxalin-2-ol (**3ac**).

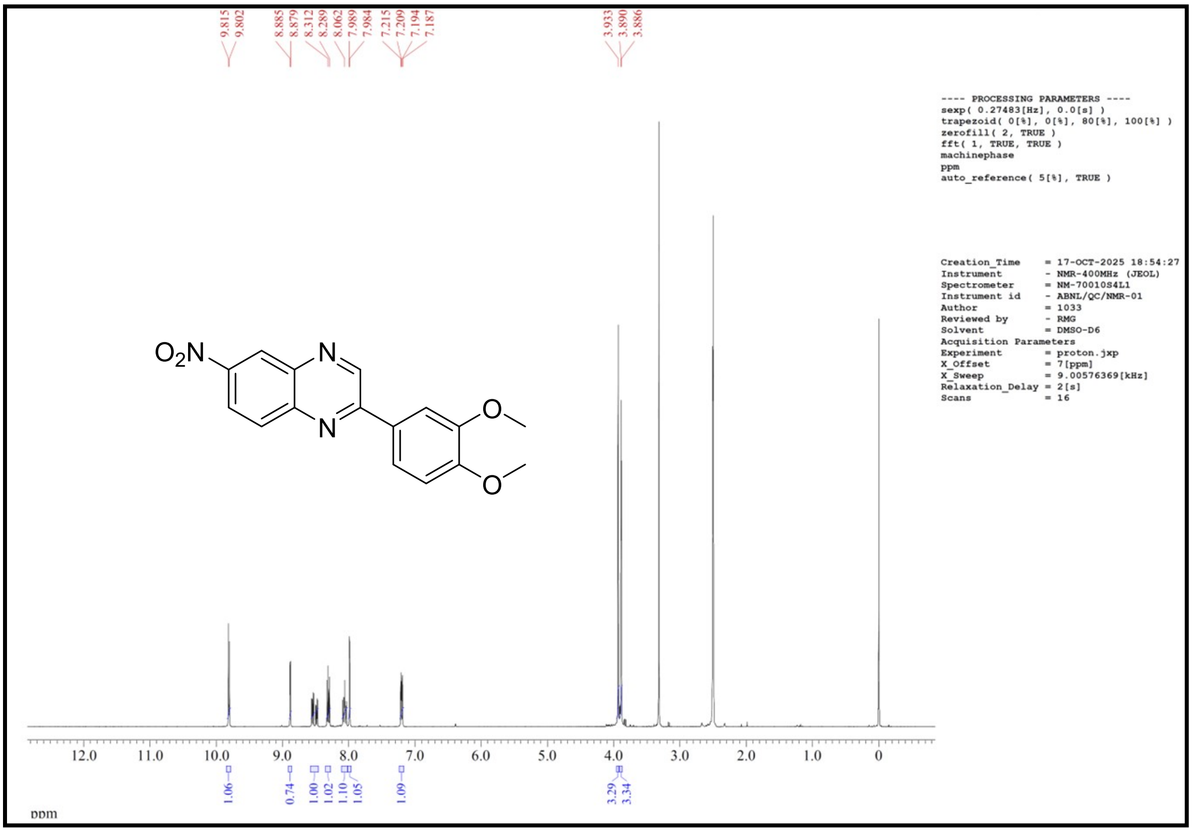

Figure S26. ^{1}H-NMR spectrum of 2-(3,4-dimethoxyphenyl)-6-nitroquinoxaline (**3ad).**

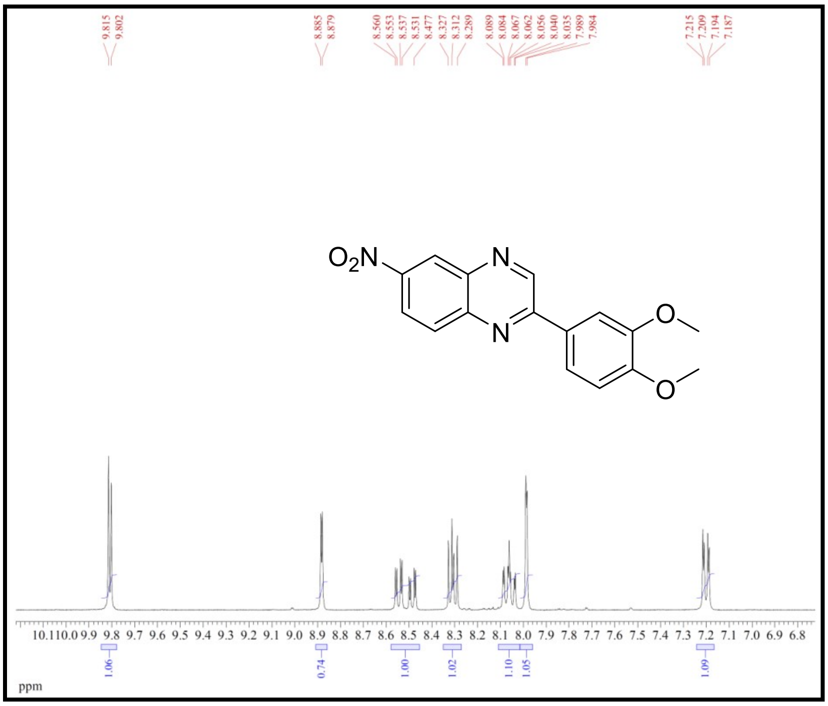

Figure S27. Expanded ^{1}H-NMR spectrum of 2-(3,4-dimethoxyphenyl)-6-nitroquinoxaline (**3ad).**

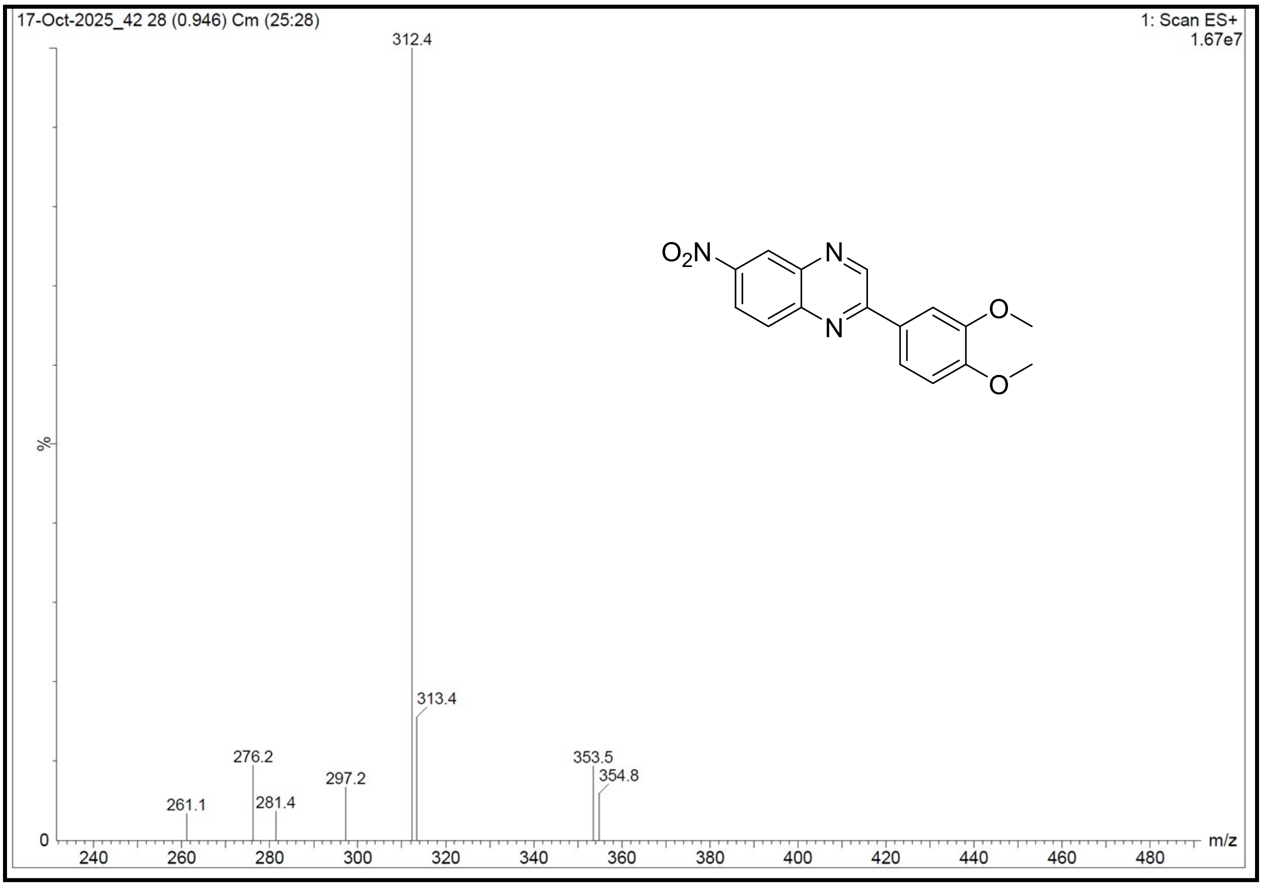

Figure S28. Mass spectrum of 2-(3,4-dimethoxyphenyl)-6-nitroquinoxaline (**3ad).**

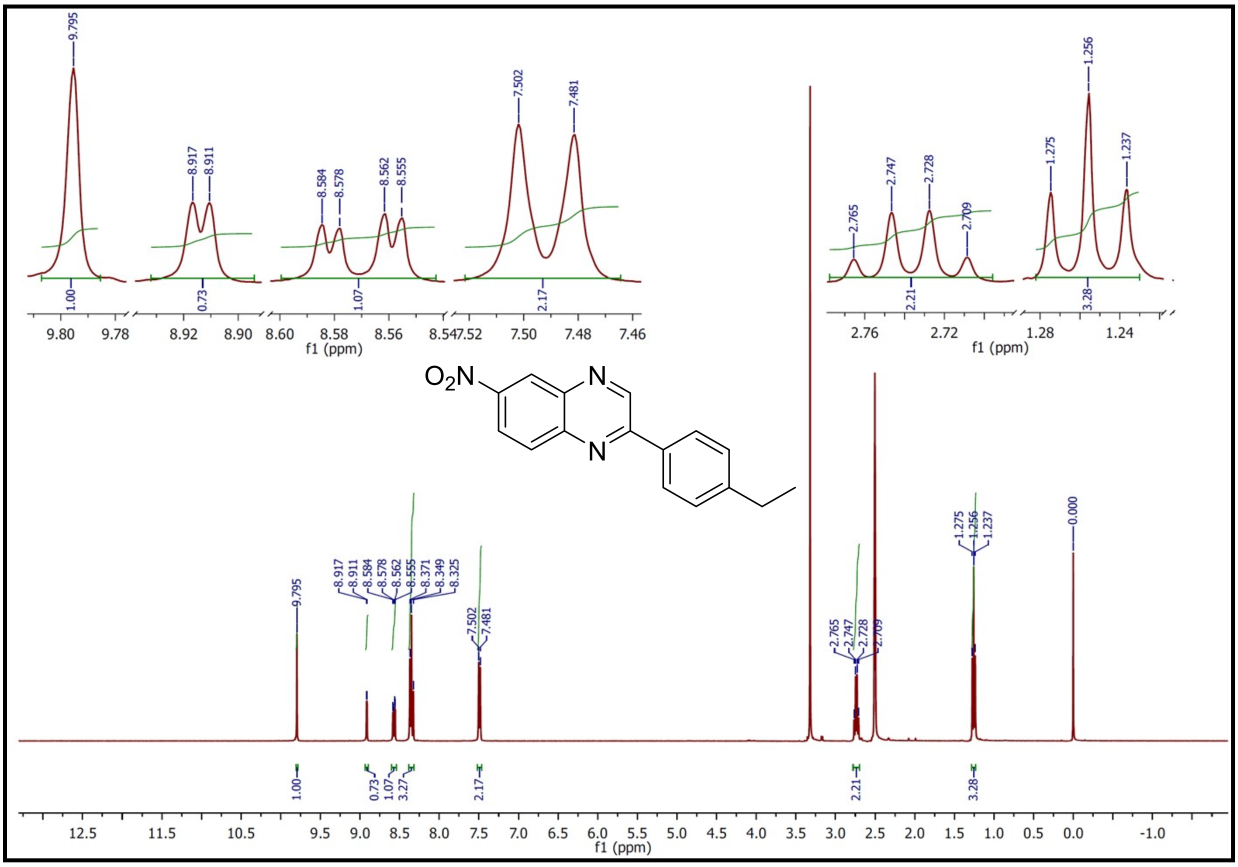

Figure S29. ^{1}H-NMR spectrum of 2-(4-ethylphenyl)-6-nitroquinoxaline (**3ae).**

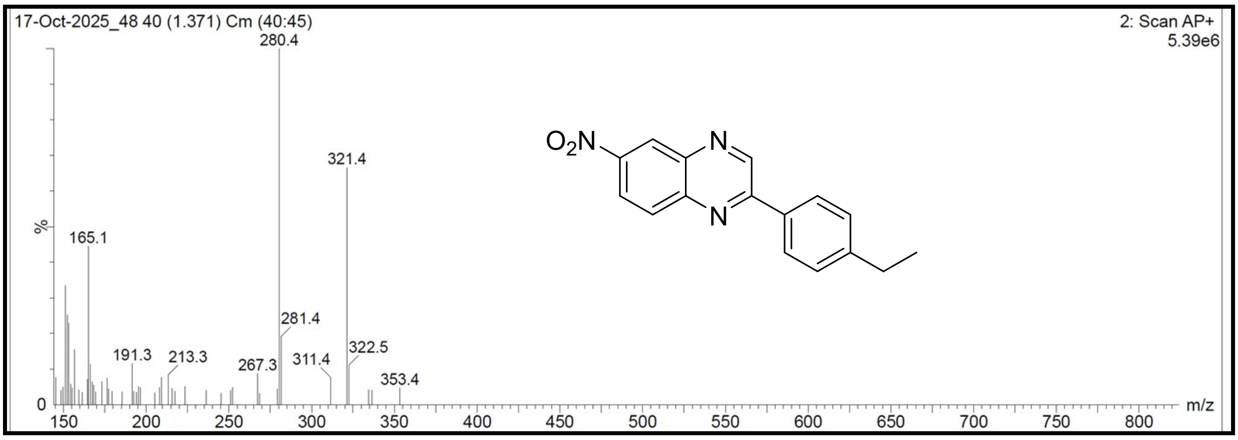

Figure S30. Mass spectrum of 2-(4-ethylphenyl)-6-nitroquinoxaline (**3ae).**

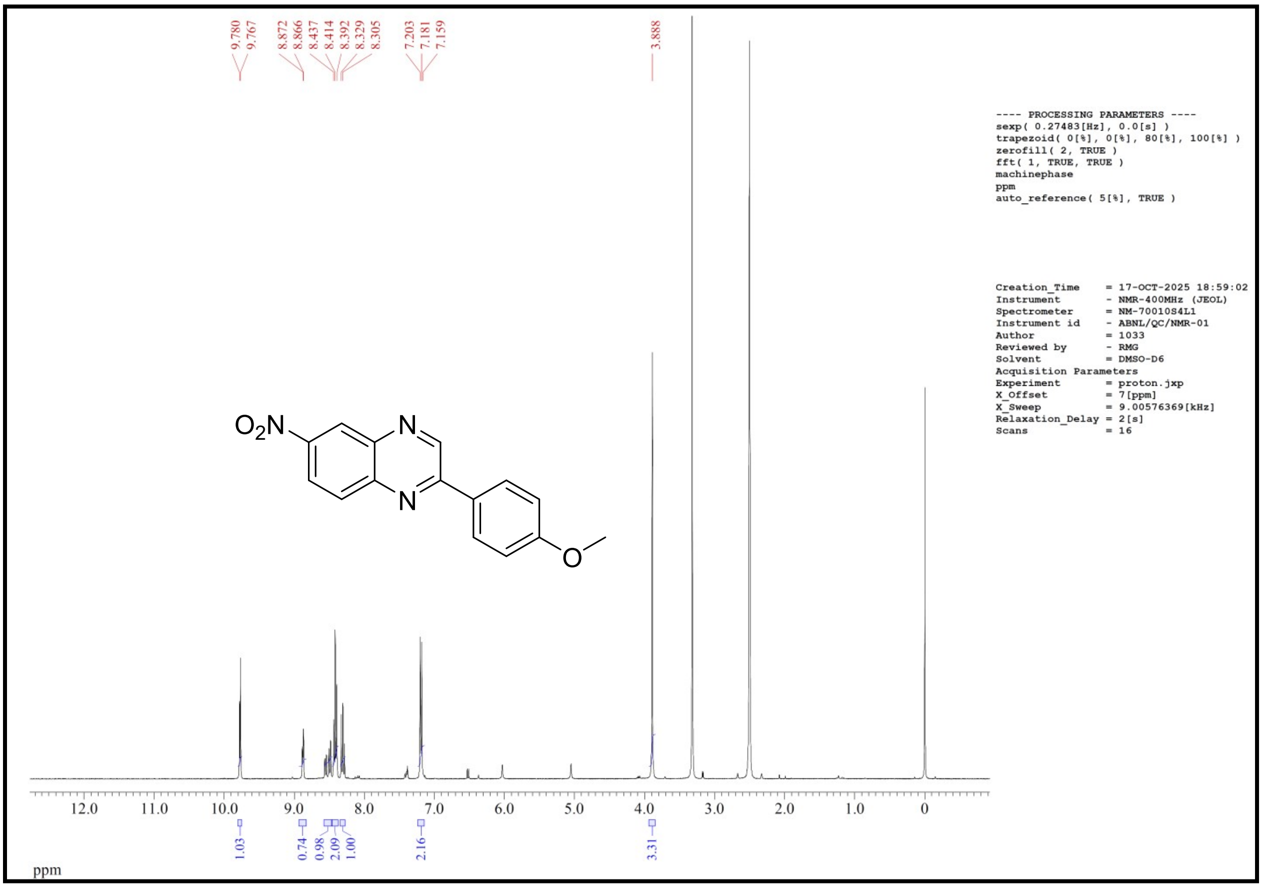

Figure S31. ^{1}H-NMR spectrum of 3-(4-methoxyphenyl)-7-nitro-1,2-dihydroquinoxaline (**3ag**).

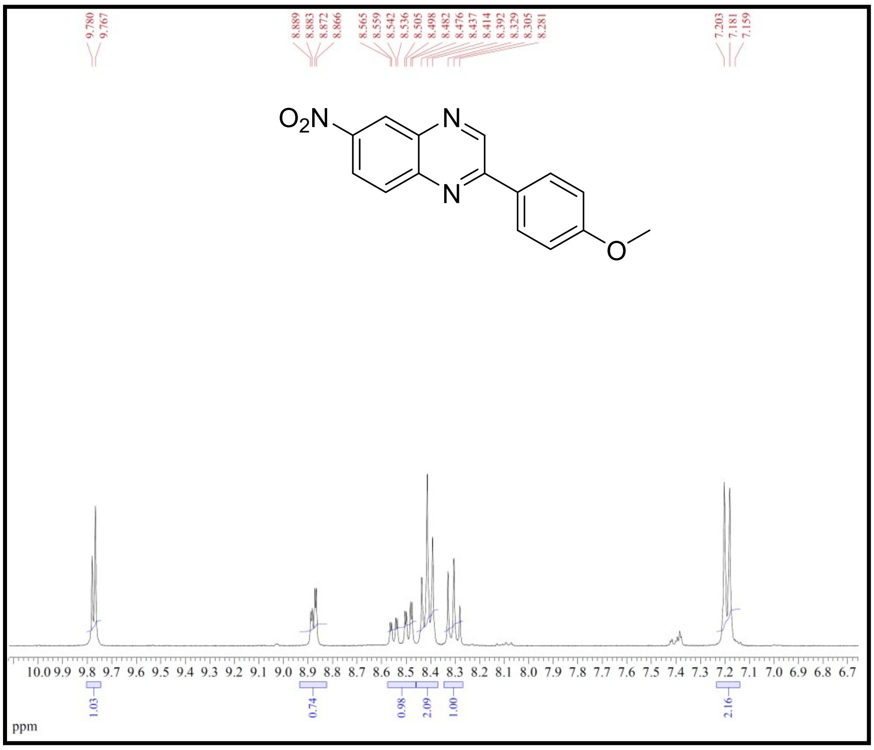

Figure S32. Expanded ^{1}H-NMR spectrum of 3-(4-methoxyphenyl)-7-nitro-1,2-dihydroquinoxaline (**3ag**).

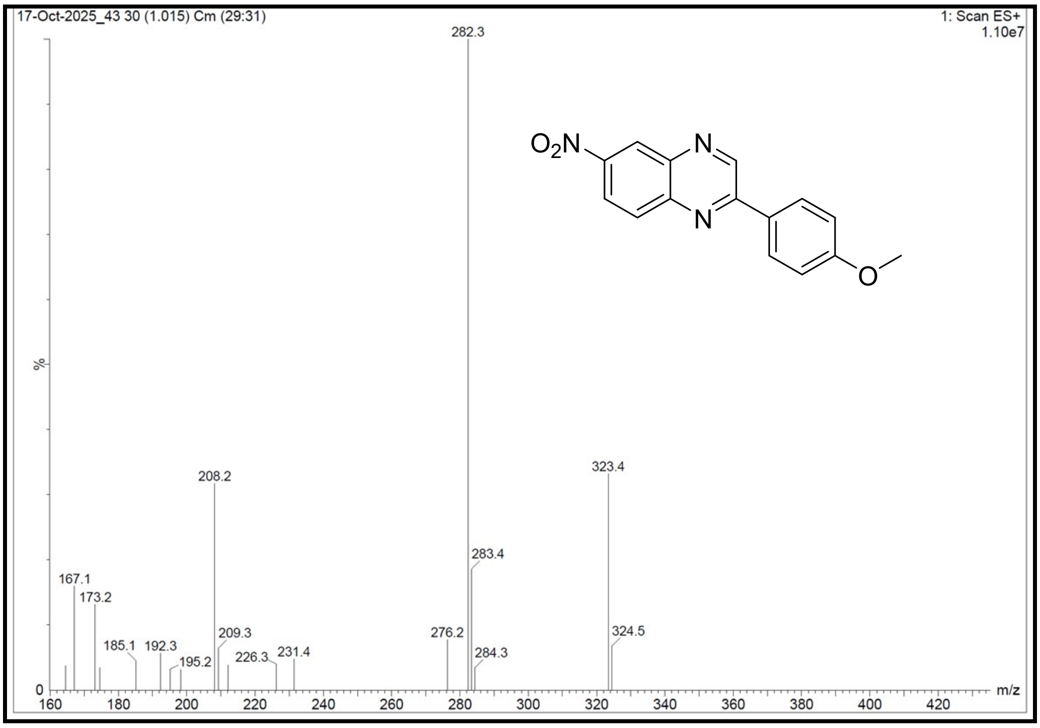

Figure S33. Mass spectrum of 3-(4-methoxyphenyl)-7-nitro-1,2-dihydroquinoxaline (**3ag**).

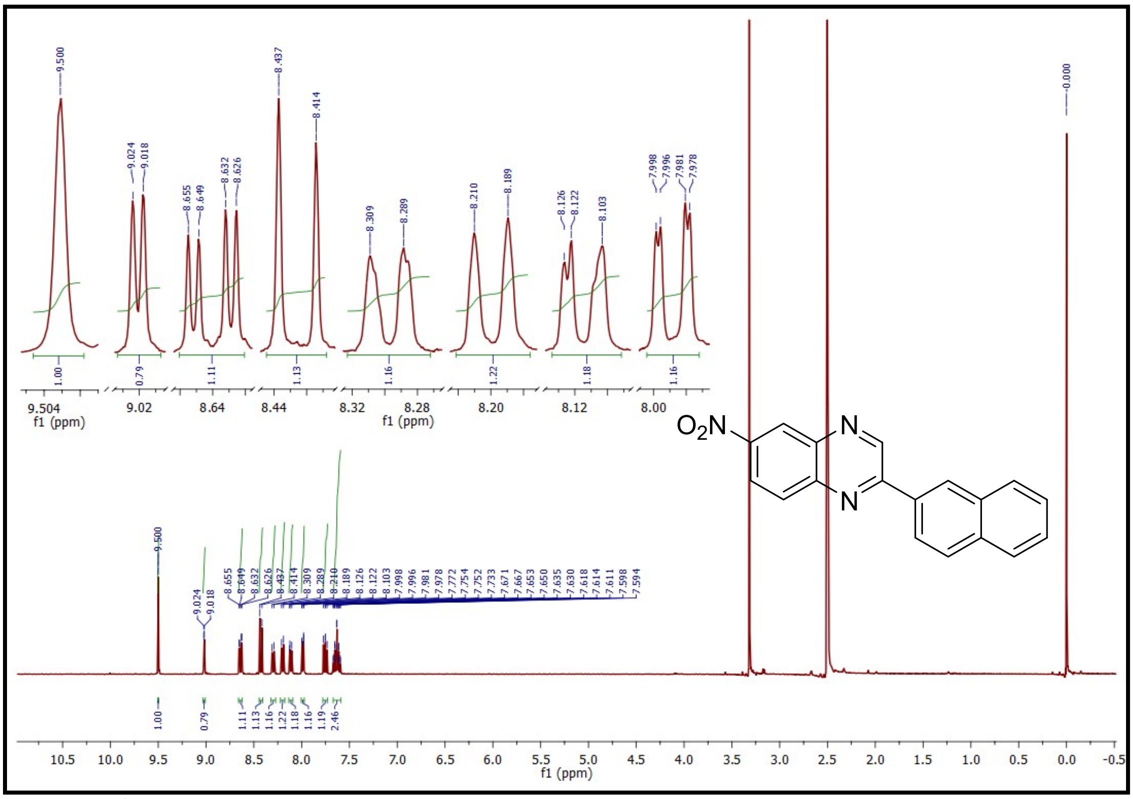

Figure S34. ^{1}H-NMR spectrum of 2-(naphthalen-2-yl)-6-nitroquinoxaline (**3ai**).

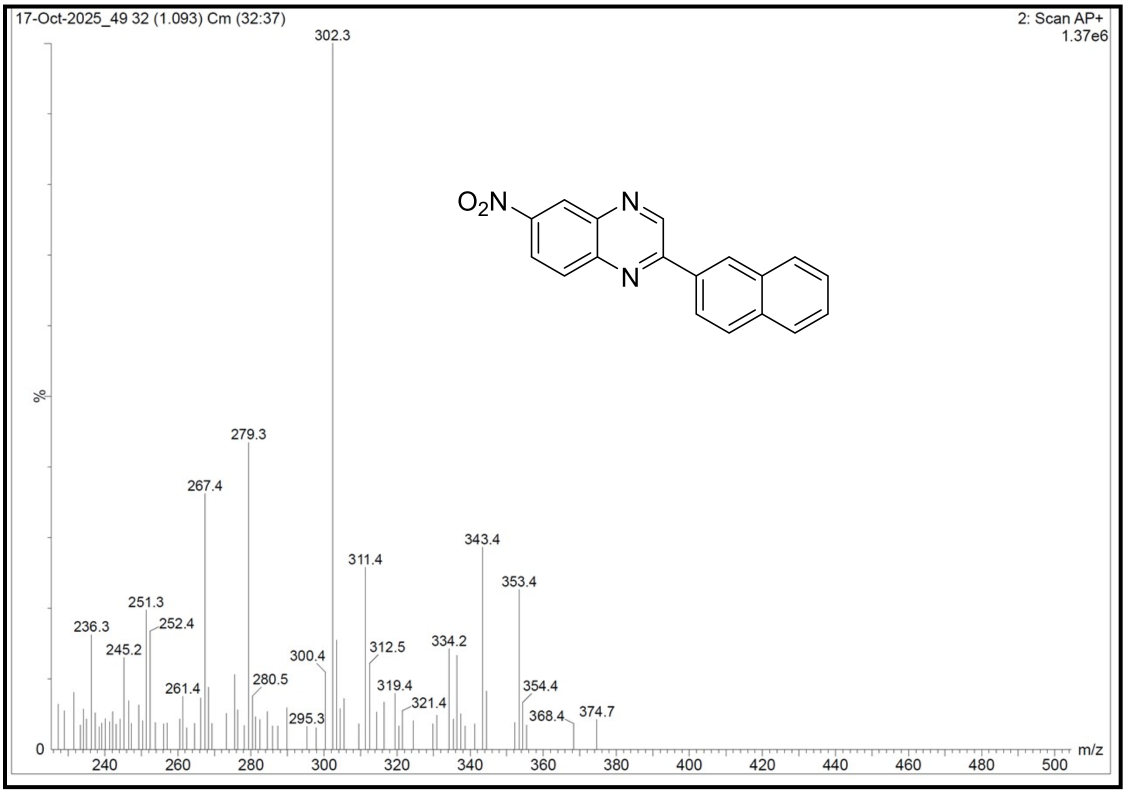

Figure S35. Mass spectrum of 2-(naphthalen-2-yl)-6-nitroquinoxaline (**3ai**).

SI-4: Mass spectral Evidence for the formation of intermediates **I** and **III** in the synthesis of 2-(4-bromophenyl)quinoxaline (**3d**) *via* the reaction between *o*-phenylenediamine (**1a**) and 4-bromophenacyl bromide (**2d**) under a nitrogen atmosphere at 55–60°C.

Plausible Mechanistic Pathway:

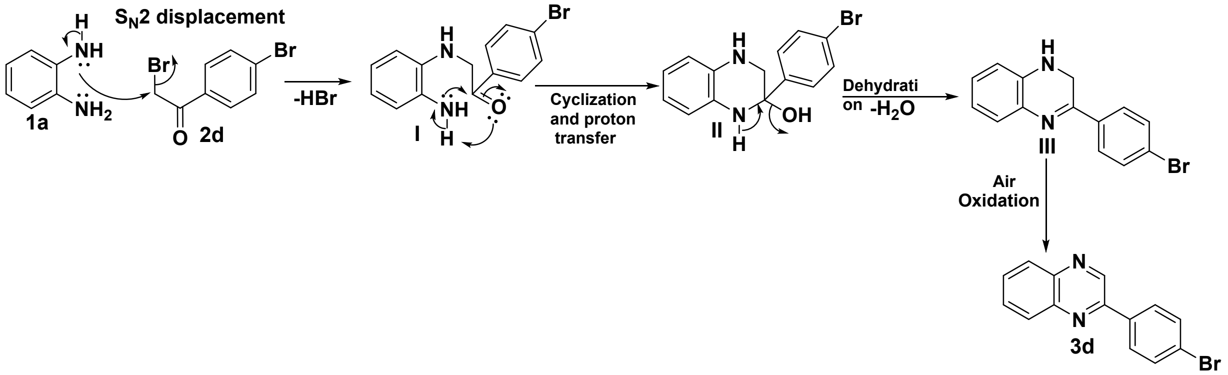

To support the mechanistic pathway, a control experiment was conducted using *o*-phenylenediamine (**1a**) and 4-bromophenacyl bromide (**2d**) under a nitrogen atmosphere at 55–60°C. The reaction was carefully monitored to detect the formation of the proposed intermediates, **I** and **III**. The samples of the crude reaction mixture were collected at very short time intervals (25–30 sec, sample 1; 40–45 sec, sample 2) and immediately analyzed by

mass spectrometry. The corresponding mass spectra for samples 1 and 2 are provided in the Supporting Information (Figures S36 and S37).

The mass analysis of sample 1 showed a prominent peak at *m/z* 305 ($[M+H]^+$), corresponding to intermediate **I**, along with a minor peak at *m/z* 289.3 ($[M+H+2]^+$), indicating the formation of intermediate **III**. In sample 2, the relative intensity of the peak at *m/z* 289.1($[M+H+2]^+$) increased significantly, while the peak at *m/z* 305.1($[M+H]^+$) decreased, inferring the progression of the reaction from intermediate **I** to **III**. Additionally, a low-intensity peak at *m/z* 287.1 ($[M+H+2]^+$) was attributable to the final product (**3d**). These observations provide experimental support for the formation of key intermediates **I** & **III** and are consistent with the proposed reaction pathway, including the rapid oxidative aromatization step that occurs likely upon exposure to ambient air.

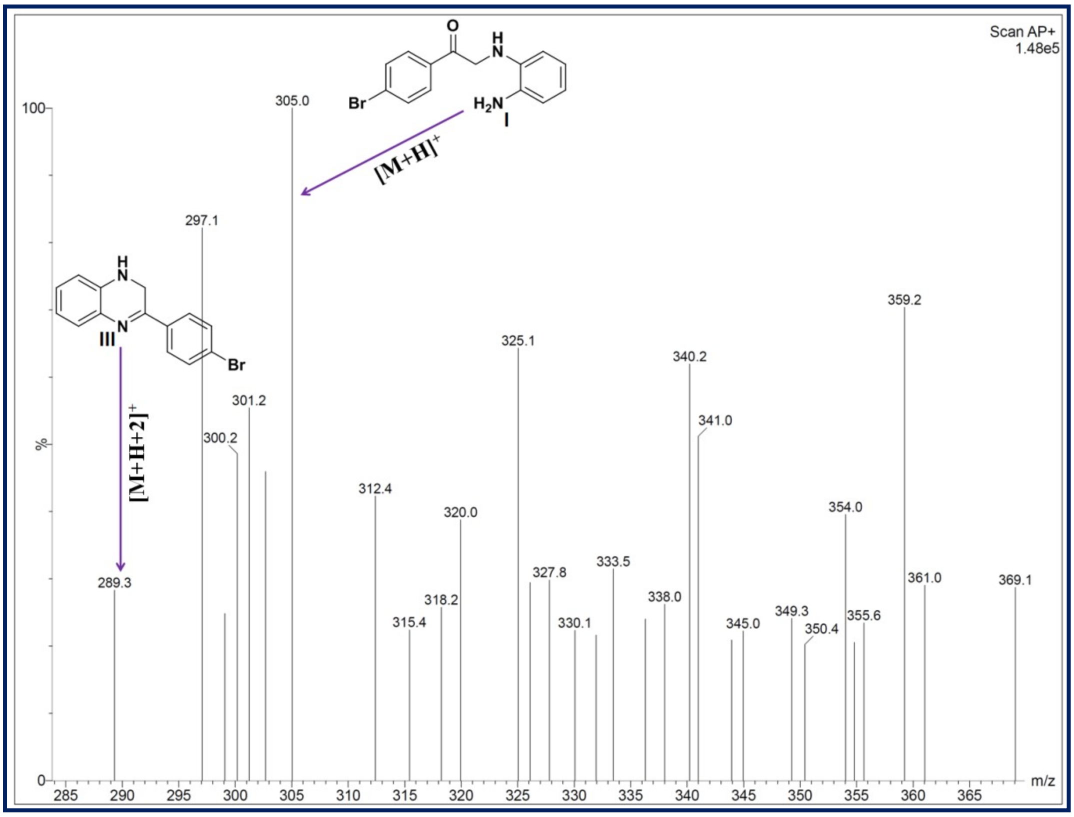

Figure S36. Mass spectrum of sample 1.

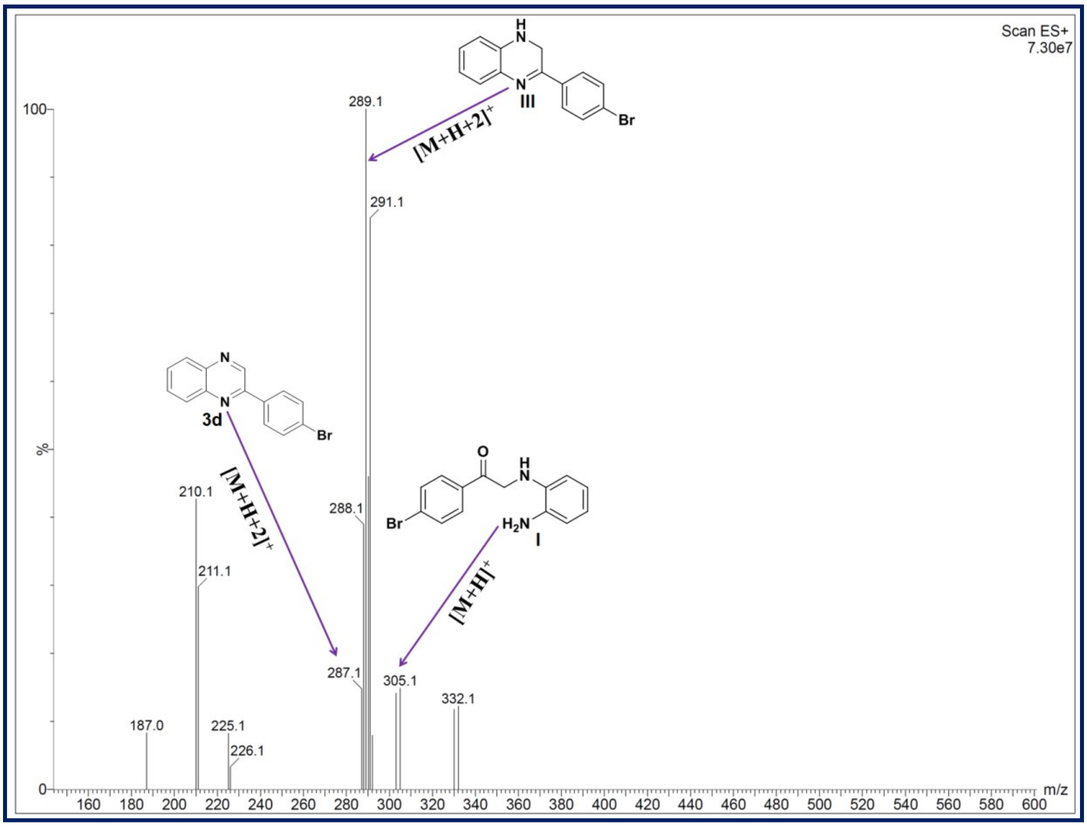

Figure S37. Mass spectrum of sample 2.
